# Transformative Masked Ortho‐Quinone Chemistry: From Direct Guaiacol Activation to Lignin‐Based Adhesives

**DOI:** 10.1002/anie.5604332

**Published:** 2026-09-01

**Authors:** Dominik P. Hoch, Keven Walter, Oliver Sipura, Jana Falkenhagen, Xinyang Peng, Leon Gerndt, Hamy Nguyen, Hans G. Börner

**Affiliations:** ^1^ Humboldt‐Universität zu Berlin, Department of Chemistry Berlin Germany; ^2^ Bundesanstalt für Materialforschung und ‐prüfung, BAM Department of Materials Chemistry Berlin Germany

**Keywords:** bioinspired two‐component resin, green chemistry, mussel inspired adhesives, sustainable bio phenol materials, transforming chemistry

## Abstract

Guaiacol moieties (1‐hydroxy‐2‐methoxy‐benzenes) are among the most abundant structural motifs in renewable biophenolic feedstocks, representing a potential industrial‐scale stream from softwood lignin through biorefinery valorization. Here, the transformative chemistry of masked ortho‐quinones (MoQs) is introduced, yielding reactive guaiacol derivatives to enable thiol‐guaiacol connectivity (TGC) polymers. Phenyliodine(III)diacetate (PIDA) oxidizes guaiacols to chemically pure MoQs within minutes at room temperature, generating activated triguaiacol monomers that undergo thiol‐MoQ Michael‐type polyaddition with multithiols. This bulk polymerization leads to TGC‐adhesives, reaching professional performance lap‐shear strengths up to 16.9 ± 0.9 MPa. The robust strategy is extended to directly activate technical lignin, generating curable multi‐MoQ prepolymers which bond aluminum with lap‐shear strengths of 9.1 ± 0.7 MPa, positioning MoQs as a sustainable platform chemistry to bridge from lignin to high‐performance materials.

## Graphical abstract

Renewable guaiacols are rapidly oxidized by phenyliodine(III)diacetate to masked ortho‐quinones, giving a new class of monomers that undergo selective Michael‐type polyaddition with multithiols to from thiol‐guaiacol connectivity‐bearing polymers. The activation applies to discrete monomers and directly to lignin, enabling two‐component adhesives that bridge sustainable aromatic feedstocks to high‐performance materials.

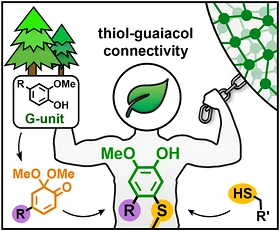

## 1 Introduction

A central challenge in polymer science is decoupling polymer materials from fossil feedstocks, lowering their climate burden and long‐term environmental impact [1, 2, 3]. Commodity polymer materials benefit from established recycling pathways and emerging upcycling strategies, [4, 5, 6] but polymeric adhesives remain a unique challenge, as thin bondlines and minor fractions complicate recovery [7]. Yet adhesives offer great potential for realizing a circular economy, [8, 9] enabling streamlined assembly, composite integration, and advanced secondary functions such as conductivity [10], damping [11], seawater resistance [12], or debonding on demand [13].

Bioinspired design has regained tremendous momentum as a capable route to purpose adapted, sustainable adhesives [14, 15, 16, 17]. Marine mussel adhesive proteins, rich in catechols from 3,4‐dihydroxyphenylalanine (Dopa) residues [18], have inspired diverse sustainable bonding concepts, ranging from the renaissance of protein‐based glues, reversible biomedical adhesives from α‐lipoic acid [19], to lignin inspired structural adhesives for electrochemically debonding on command [20], to reversible wet paper reinforcing adhesives by in‐sheet polymerization [21].

Recently, thiol‐quinone Michael‐type polyaddition emerged, yielding thiol‐catechol connectivities (TCCs) as potent bonding motifs by reacting multi‐quinones with multi‐thiols to form linear, branched, or resin‐type TCC‐polymers [22, 23, 24, 25, 26]. The quinones are derived from enzymatic processing of tyrosine‐bearing peptides [25, 27], 2‐iodoxybenzoic acid (IBX) oxidation of commodity bisphenols [28], chemical [29, 30], or electrochemical [31] oxidation of Dopa‐peptides [30] or through demethylation‐oxidation of lignin guaiacol (G, 1‐hydroxy‐2‐methoxy‐benzene) units [32].

Despite advances in sourcing TCC‐precursors from highly available lignin streams [33, 34], the feedstock shows variability due to origin and processing [35]. Instead, biorefinery‐derived biophenols offer a promising alternative [36, 37], as softwood lignin contains ∼90 mol% G‐units [37], representing an abundant class of bioaromatics with projected multimillion‐ton availability in a global biorefinery scenario [38]. Guaiacols are already used to synthesize renewable phenolic monomers [39] for isocyanate free polyurethane [40], epoxy [41, 42], and benzoxazine [43, 44] materials. However, these reactions usually consume phenolic hydroxyl groups, eliminating key interaction sites [45, 46, 47], whereas TCC‐polymers retain catechol hydroxyl groups to preserve potent bulk and interfacial interactions [32]. Established oxidation methods to generate *ortho‐*quinones have some limitations as tyrosinase processing showed substrate specificities [48, 49], sodium periodate requires aqueous conditions, and IBX is considered explosive [50]. An alternative strategy, first described for small organic molecules, converts guaiacols into masked *ortho‐*quinones (MoQs) using phenyliodine(III)diacetate (PIDA), an easily handled, non‐explosive oxidant [51, 52, 53, 54]. MoQs are established reactive intermediates that undergo Michael‐type additions [55, 56] with thiols to yield thiol‐substituted guaiacols [57].

Here, we establish a polymerization platform by selectively reacting multi‐MoQs with multi‐thiols to yield thiol‐guaiacol connectivity (TGC) polymers (Figure 1), unlocking a direct pathway from sustainable guaiacol feedstocks to high‐performance adhesive materials.

**FIGURE 1:**
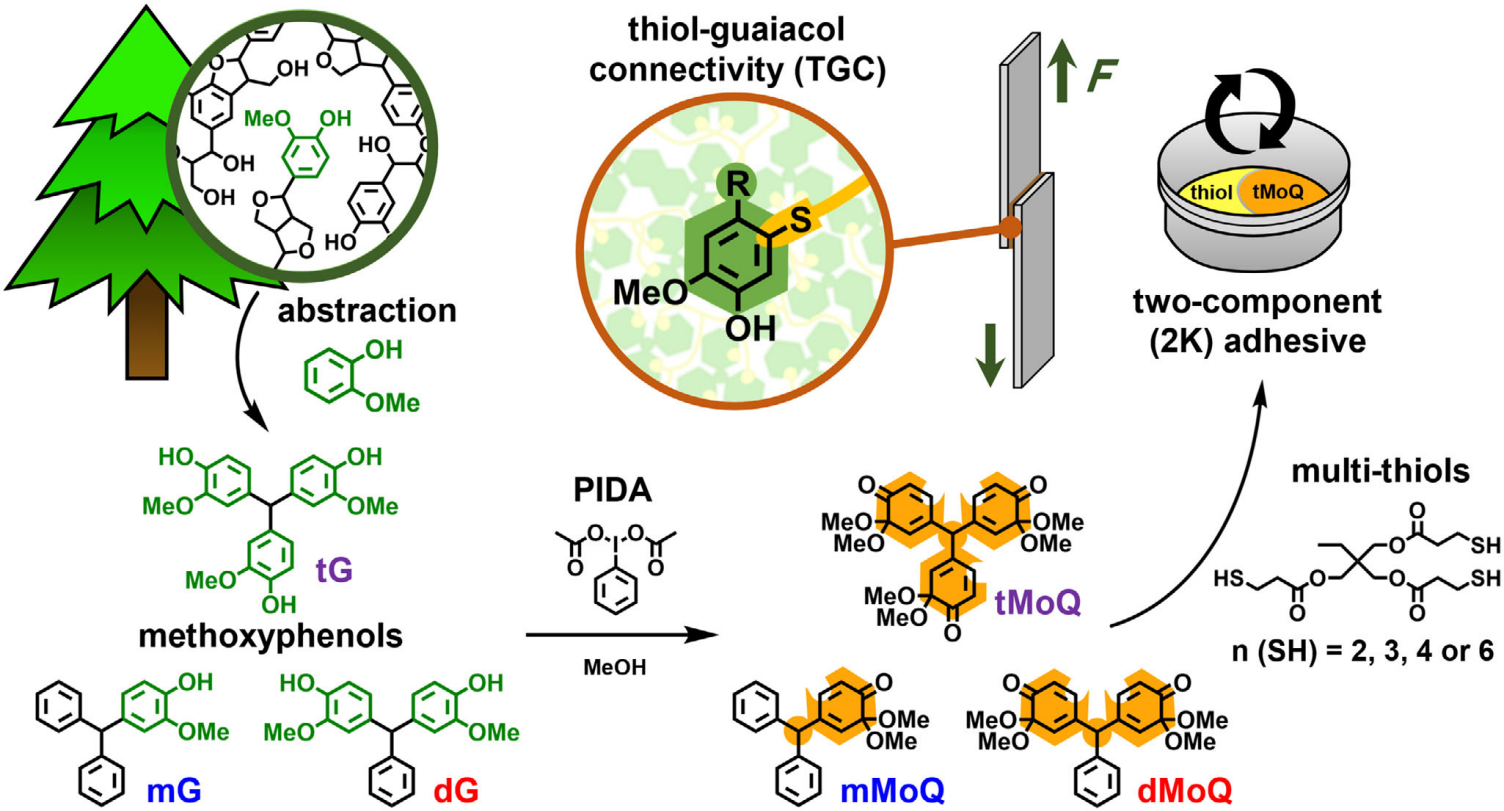
Design concept of lignin‐based TGC chemistry. Synthesis of mono‐, di‐ and trimeric guaiacols (m/d/tG) inspired by the lignin G‐motif, where activation with PIDA yields masked *ortho*‐quinones (m/d/tMoQ), that react with multi‐thiols to form TGC‐based two‐component (2K) adhesives.

## 2 Results and Discussion

A set of mono‐, di‐, and triguaiacol monomer precursors (mG, dG, tG) were synthesized by Lewis acid catalyzed electrophilic substitution of guaiacol with benzhydrol, benzaldehyde, or vanillin, respectively (Figure 1, Section S3) [58]. Subsequent oxidation of the G‐units with PIDA in methanol at room temperature (r.t.) yielded the corresponding masked *ortho‐*quinones (mMoQ, dMoQ, tMoQ), as confirmed by ^1^H‐NMR, IR and UV/vis spectroscopy (Section S5.1). Notably, all MoQs could be isolated as pure, stable powders. For instance, tMoQ remained shelf‐stable for more than five months under ambient, dark storage in air (Section S5.5), unlike related compounds that are often unstable in bulk, making isolation difficult [56]. The procedure promised scalability as tMoQ was readily prepared on a 20 g lab‐scale in 81% isolated yield and 98% purity, with acetic acid and potentially recyclable iodobenzene [59] as the only byproducts.

The TGC‐formation was proven by reacting mMoQ with butanethiol in solution at r.t. (Figure 2a). The reaction proceeded most rapidly in methanol and achieved full conversion after ∼33 h, which is markedly slower than thiol‐quinone Michael‐type additions [22]. Interestingly, dMoQ and tMoQ also showed rapid initial thiol addition, but conversion plateaued at 83% (1.7/2) and 75% (2.3/3), respectively. NMR and UHPLC confirmed intact, unreacted MoQ groups and ruled out competing side reactions (S.I. Section S5.2). The origin for the unusual conversion plateaus was investigated in more detail (Section S5.2). Steric shielding combined with conformational statistics initially appeared to provide a plausible explanation for the observed plateauing behavior. However, computed [60, 61] rotational barriers proved low enough to be overcome at room temperature, ruling out this effect as the sole determining factor. Further investigation suggested that the incomplete conversion might instead arise due to a progressive reduction of Michael acceptor activation along the substitution cascade, making subsequent thiol addition steps less favorable/reversible. MoQ units quantitatively exchange both methoxy groups by deuterated MeOH‐d_4_, as confirmed by ^1^H‐NMR and ESI‐MS, which indirectly suggested cationic species as the most reactive Michael acceptor sites. This cationic species becomes notably less reactive after the first thiol addition in dMoQ and the second in tMoQ. Interestingly, calculations show a more pronounced reduction for tMoQ than for dMoQ, which reflects the observed conversion plateaus. Despite high but non‐quantitative conversion, TGC‐formation proceeded highly regioselectively, yielding exclusively the 6’‐addition product, as confirmed in the mMoQ model by 2D‐NMR and single‐crystal x‐ray analysis (Section S5.3 and S3.5). This contrasts with TCC‐formation, which typically gives mixtures of 5’‐, 2’‐, and 6’‐substituted isomers [22].

**FIGURE 2:**
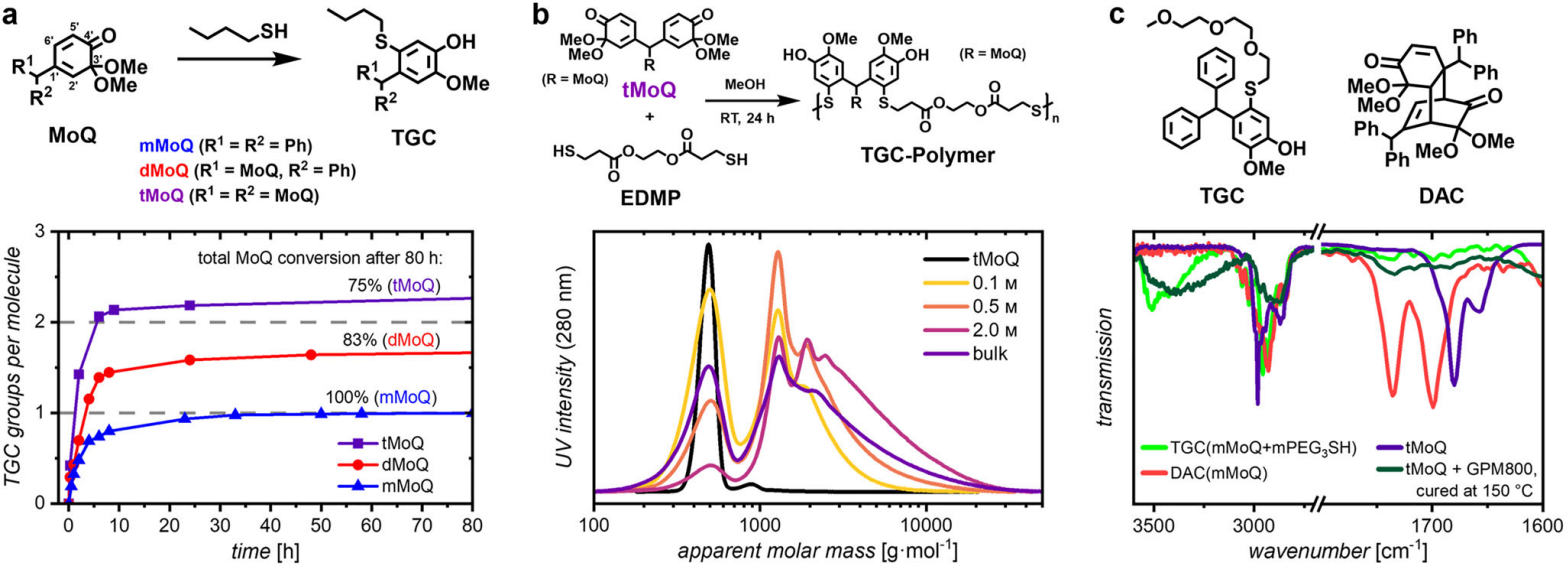
Reactivity studies of MoQ compounds. TGC‐formation of masked ortho‐quinones mMoQ, dMoQ, and tMoQ with butanethiol with MoQ/T = 1/1 in MeOH at a concentration of 0.1 m (top) and corresponding UHPLC conversion profiles (bottom) (a). Pseudo‐linear polymerization of tMoQ with EDMP at MoQ/T = 3/2 (top) and GPC analysis of products formed at r.t. after 24 h in methanol or in bulk (bottom) (b). Chemical structures of TGC and DAC model compounds (top) and IR spectra of TGC and DAC model compounds, tMoQ and a tMoQ/GPM800 adhesive with MoQ/T = 1/0.6, cured at 150°C for 16 h (bottom) (c).

Consistent with the model, dMoQ only oligomerizes with dithiols at r.t., whereas tMoQ allowed pseudo‐linear TGC‐polymerization with two preferentially reacting MoQ sites (Figure 2b). tMoQ polymerizes with ethylene glycol di(mercaptopropionate) (EDMP) in methanol or in bulk at 3/2 MoQ/thiol ratios, reaching up to 73% conversion after 24 h (^1^H‐NMR, Section S5.4). Gel‐permeation chromatography (GPC) revealed broad molecular‐weight distributions with low‐molecular‐weight species (*∼*1300 g·mol^−1^) and high‐molecular‐weight fractions that intensified with increasing monomer concentration, reaching *M*_max_≈ 10‐30 kg·mol^−1^.

To improve MoQ‐reactivity, TGC‐formation was studied at elevated temperatures (Section S5.5). Thermogravimetric analysis (TGA) under argon confirmed stability of tMoQ up to 190°C, while storage under air showed changes in ^1^H‐NMR spectra only above 110°C, where tMoQ preserved stability for ≤8 h. GPC analysis of heated tMoQ showed peak broadening and the formation of higher molecular weight fractions, suggesting partial crosslinking, potentially through Diels–Alder (DA) reactions [56]. To validate this mechanism, mMoQ was heated to 90°C for 6 days, giving an isolatable DA‐dimer as confirmed by 2D‐NMR (S.I. Section S5.6). IR spectra revealed a marker carbonyl band for Diels–Alder connectivities (DACs) at 1737 cm^−1^, distinct from the conjugated MoQ carbonyl at 1678 cm^−1^ (Figure 2c). Importantly, the newly formed Michael‐system in the DA‐dimer showed no reactivity toward thiols.

DAC‐formation was quantified by reacting mMoQ with triethylene glycol monothiol (mPEG_3_SH) in bulk for 16 h at 30–120°C (Section S5.7). UHPLC showed that increasing temperatures enhanced MoQ conversion and concomitantly raised DAC‐formation from 3% at 30°C to 28% at 120°C. Similar trends were observed when reacting tMoQ with mPEG_3_SH (Section S5.8). Notably, IR confirmed that under network‐forming conditions, tMoQ reacted with the tetrathiol pentaerythritol‐tetrakis(propylene‐2‐mercaptoethan‐1‐ol) (GPM800, S.I. Section S5.10) with neglectable DAC‐formation even at 150°C under MoQ/T = 1/0.6. This suppression likely arises from constrained MoQ mobility during network formation, preventing alignment required for DA reactions.

Higher reaction temperatures increased MoQ conversion through the TGC‐pathway, which dominated over DA crosslinking. On this basis, tMoQ was evaluated in 2K adhesive settings, using different multithiols including the dithiol EDMP, the trithiol 1,1,1‐tris(hydroxymethyl)ethane tris(3‐mercaptopropionate) (TTMP), the tetrathiol pentaerythritol‐tetrakis(3‐mercaptopropionate) (PTMP), and the hexathiol dipentaerythritol‐hexakis(3‐mercaptopropionate) (DHMP) (Section S5.11).

Differential scanning calorimetry (DSC) analysis of 1/1 MoQ/T mixtures showed onset temperatures of 78°C for EDMP, 83°C for PTMP, 95°C for TTMP, and 100°C for DHMP, correlating with thiol concentration and viscosity. Higher thiol contents led to earlier onsets (EDMP: 8.4 mmol·g^−1^, PTMP: 8.2 mmol·g^−1^), whereas lower contents shifted onsets higher (TTMP: 7.5 mmol·g^−1^, DHMP: 7.7 mmol·g^−1^). The high onset of DHMP might additionally be attributed to high viscosity of 2350 mPa·s compared to ≤450 mPa·s for the other thiols. Despite the high DSC onset temperatures, isothermal rheology revealed substantially earlier curing, which is a known discrepancy as crosslinking changes moduli before exothermic reactions become detectable [62]. At 60°C, all mixtures except EDMP underwent sol‐gel transitions within hours (Figure 3a), and gelation accelerated with increasing crosslinker functionality in agreement with the Flory–Stockmayer model [63, 64]. Remarkably, tMoQ/DHMP gelled at 20°C, still offering practical pot life of *t*_sg_ = 28 h. This decreased at higher temperatures to 16 h at 30°C, 11.4 h at 40°C, and 6.4 h at 50°C (Section S5.12). A practical advantage over conventional 2K adhesives may arise from the methanol liberated during TGC‐formation, which transiently plasticizes the formulation and assists both wetting and network formation before evaporation.

**FIGURE 3:**
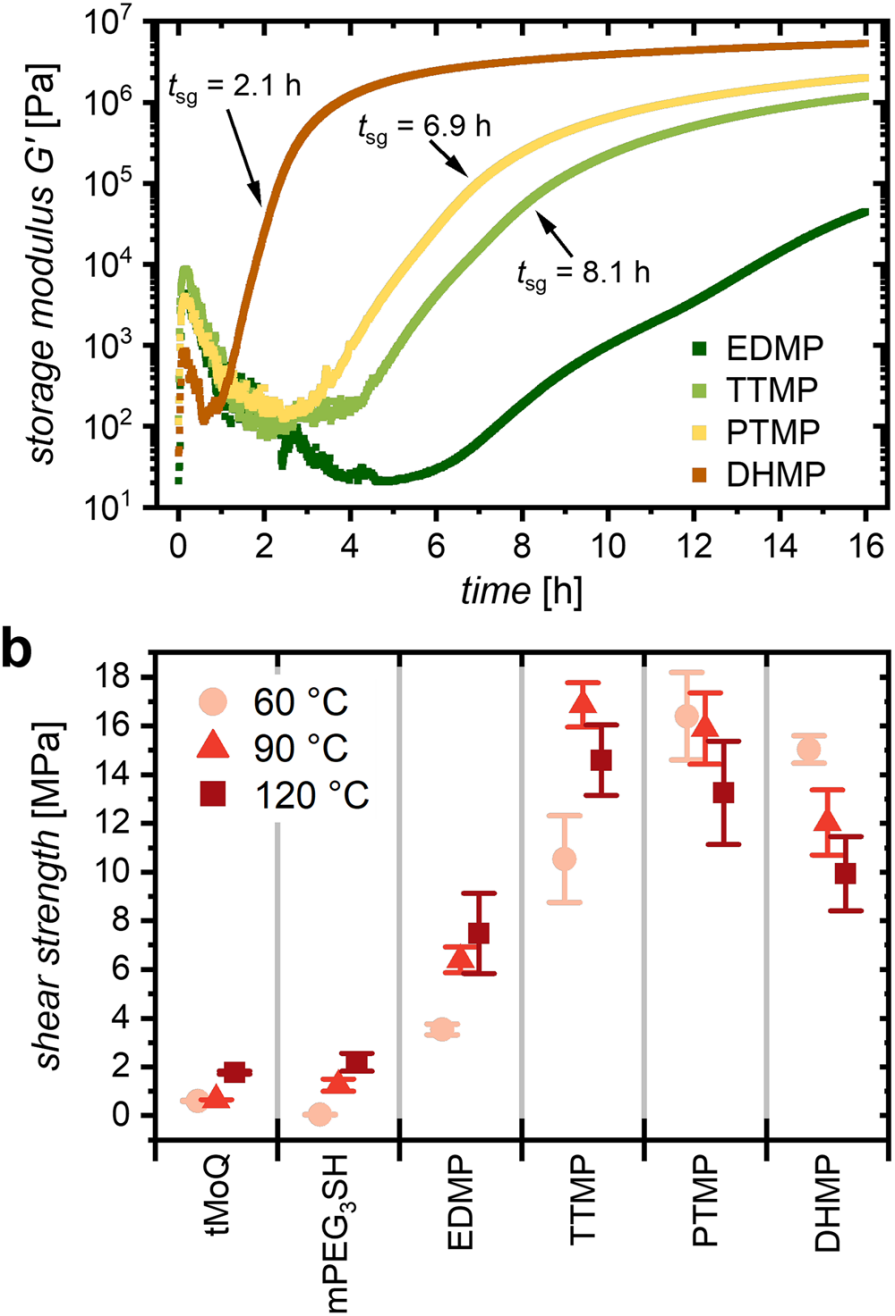
Performance of TGC‐adhesives consisting of tMoQ and different multithiols. Isothermal rheology of 2K formulations at 60°C with MoQ/T  =  1/1, *γ * =  0.1% and *ω * =  10 rad·s^−1^. *t*_sg_ denotes sol‐gel transition (a). Lap‐shear strengths of aluminum joints with MoQ/T = 1/1 mixtures, cured for 16 h. Error bars represent standard deviation (n = 3) and statistical significance tests are provided in Section S5.13 (b).

To access structure–property relationships, lap‐shear tests were performed on aluminum joints bonded with four different 2K adhesives (MoQ/T = 1/1) that were cured at 60°C–120°C (Figure 3b and Section S5.13). Time‐dependent curing of tMoQ/PTMP at 60°C showed emerging adhesive strength after 8 h and a plateau after 12 h, which was consistent with the rheological sol‐gel transition after 6.9 h. Accordingly, 16 h was set as a standardized full‐cure condition for comparing all formulations. Lower‐temperature curing was feasible, for example, for tMoQ/DHMP near r.t. at 30°C but require ∼6 d to reach comparable lap‐shear strength to 16 h curing at 60°C (Section S5.13). Further acceleration of the curing step might be possible through catalysis, as reported for Michael‐type thiol‐x additions [65] or by using aromatic thiols, which demonstrated significantly faster reactions in thiol‐quinone additions compared to aliphatic thiols [24]. In the present study, however, the 2K formulations were restricted to a defined base set to ensure comparability across structure–property relationships.

At 60°C, EDMP formed weak adhesives (3.5 MPa), whereas higher‐valency crosslinkers (TTMP, PTMP, and DHMP) reached strengths of 10.5, 16.4, and 15.0 MPa, respectively, which is in the range of strong commercial bisphenol A based 2K‐epoxy adhesives (16.0 MPa, Figure S40). Glass transition temperatures (*T*_g_) of the TGC‐adhesives remained moderate (72°C–83°C), except for EDMP and DHMP (20°C and 27°C), suggesting inefficient network formation probably due to low functionality of EDMP and high viscosity of DHMP. Predominantly cohesive failure modes mirrored these findings, suggesting strong interfacial adhesion (Section S5.13). Curing at 90°C instead, performances of EDMP and TTMP improved (6.4 and 16.9 MPa), while PTMP and DHMP declined slightly (15.9 and 12.0 MPa) despite elevated *T*_g_ values (109 and 96°C), reflecting restricted energy dissipation in the networks. Failure modes evolved accordingly, with EDMP shifting to adhesive fracture and the other adhesives transitioning to mixed failures, suggesting improved cohesion. At 120°C these tendencies intensified, with EDMP improving modestly (7.5 MPa), but TTMP, PTMP, and DHMP decreasing (14.6, 13.3, and 9.9 MPa) as *T*_g_ increased up to 151°C (tMoQ/DHMP). Failure shifted to adhesive modes, consistent with well‐crosslinked networks with reduced capability of adapting to interfacial stresses.

Together, the data map revealed a coherent structure‐temperature‐performance landscape. Higher functionality increases network density and *T*_g_ but risks brittleness, optimal adhesion arises from moderately crosslinked networks with sufficient mobility for interfacial wetting and stress dissipation. Peak performance was observed with PTMP at 60°C (*σ*  =  16.4 ± 1.8 MPa) and TTMP at 90°C (*σ* =  16.9 ± 0.9 MPa), while EDMP steadily increased and DHMP steadily declined at higher curing temperatures. Remarkably, TGC‐adhesives also exhibited rather broad substrate compatibilities (Figure S43). Steel joints reached lap‐shear strengths comparable to aluminum, whereas polymeric substrate bonds achieved 3.0–4.5 MPa, with polystyrene and polyethylene terephthalate giving the highest values. Strong bonding was also observed for softwood and hardwood, where substrate failure preceded bondline fracture. Only polypropylene showed poor bonding with TGC‐adhesives, consistent with the low surface energy and limited wettability.

Building on the PIDA activation of guaiacol multimers, the strategy was extended to directly activate technical softwood kraft lignin (Figure 4a and Section S5.15). The ^31^P‐NMR hydroxy‐pattern analysis confirmed a G‐unit‐rich structure with ∼3.6 mmol phenolic OH‐groups per gram of lignin. Treatment with PIDA in γ‐valerolactone/methanol at r.t. for 15 min generated the characteristic MoQ band at 1678 cm^−1^, which completely disappeared upon reaction with 3‐(trifluoromethyl)‐benzyl mercaptan (S.I. Section S5.15). Quantitative ^19^F‐NMR confirmed presence of fluorinated entities and ^31^P‐NMR proved absence of free thiols, supporting TGC‐formation (Section S5.15). Increasing PIDA loading up to 4 mmol·g^−1^ resulted in MoQ‐contents of up to 1.2 mmol·g^−1^ (Figure 4b), a twofold improvement over reported multi‐step routes [32]. Non‐oxidized lignin controls showed only minor thiol incorporation of ∼0.14 mmol·g^−1^ (∼10% background), suggesting low concentration of intrinsic electrophilic sites. ^31^P‐NMR of the TGC‐lignins revealed only a slight decrease in aliphatic OH‐groups from 2.08 to 1.69 mmol·g^−1^ with increasing degree of oxidation, which was consistent with mass increase upon thiol addition and supported predominant oxidation of phenolic sites. In contrast, phenolic OH‐groups showed a drastic progressive decrease from 3.61 to 1.41 mmol·g^−1^, which can be explained by incomplete MoQ conversion due to limited accessibility. This interpretation was confirmed by IR spectroscopy exhibiting residual MoQ carbonyl bands in the spectra of TGC‐lignin (Section S5.15). GPC analysis revealed a high‐molecular‐weight shoulder that broadened with increasing PIDA loading (Section S5.15). Shoulder formation only occurred during precipitation of the MoQ‐lignin and could most likely be explained by DA‐crosslinking. Combining GPC and the quantitative ^19^F‐NMR data revealed a continuous increase from 3.6 to 27 TGC‐groups per lignin molecule with increasing PIDA activation based on the network‐relevant *M*_w_. Overall, the one‐step oxidation converted up to ∼33% of phenolic OH‐groups into reactive MoQ sites, establishing a scalable chemistry to lignin‐based TGC materials.

**FIGURE 4:**
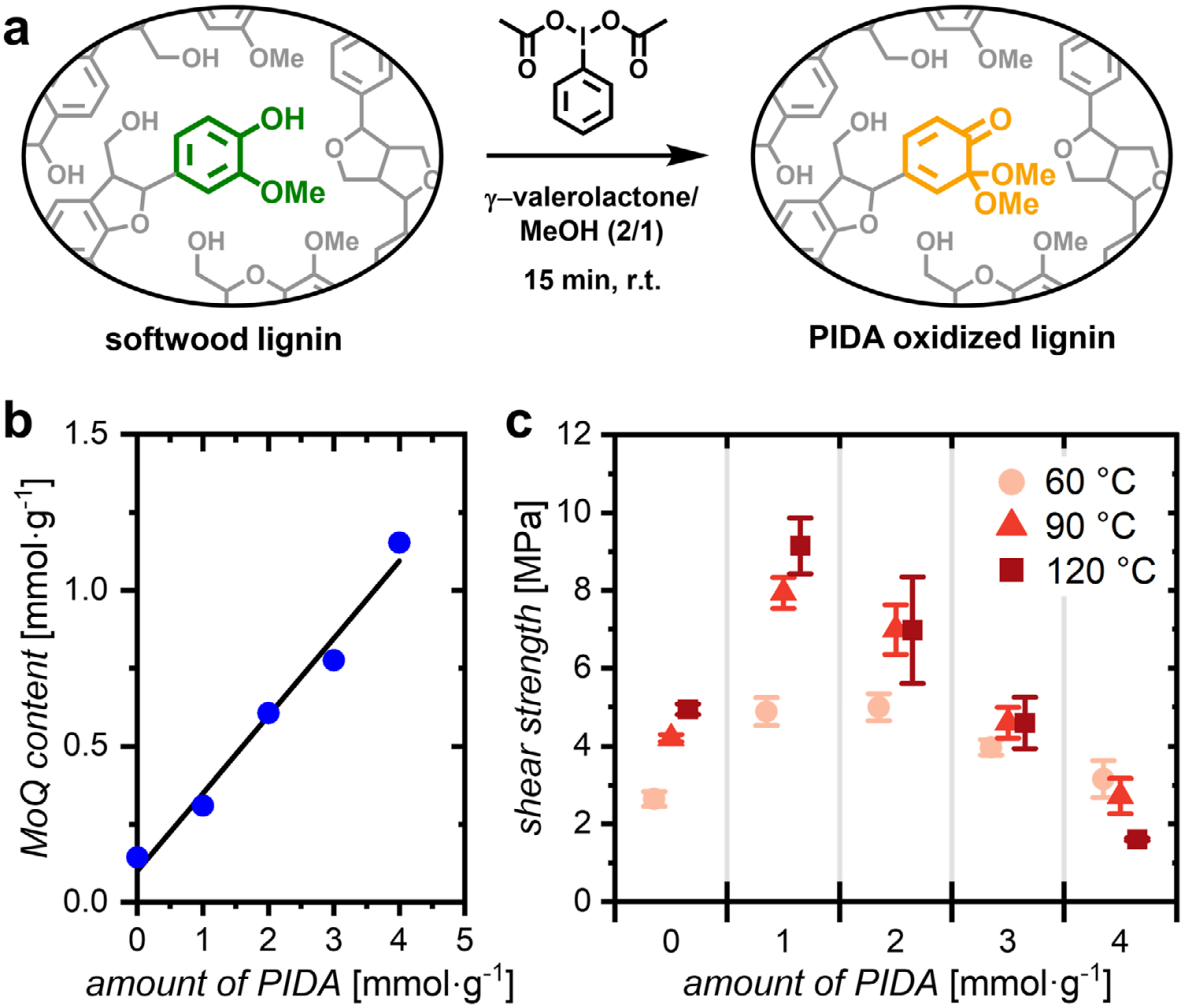
Lignin‐based 2K adhesives. Schematic representation of G‐unit oxidation in softwood lignin using PIDA (a). MoQ content of oxidized lignin quantified by ^19^F‐NMR after TGC‐formation (b). Lap‐shear strengths of aluminum joints bonded with lignin TGC‐adhesives with lignin/ETTMP_1300_ 50/50 wt.%, cured for 16 h. Error bars represent standard deviation (*n* = 3) and statistical significance tests are provided in Section S5.17 (c).

For 2K adhesive preparation, PIDA‐oxidized lignin (OxL) was blended at 50/50 percentage by weight (wt.%) with the liquid trithiol ETTMP_1300_ (ethoxylated TTMP, *M*_n_ =  1300 g·mol^−1^) [32]. Unlike tMoQ‐based adhesives, the lignin‐based formulation already developed substantial shear strength after curing for 2 h at 120°C, followed by a slower increase (Section S5.17). This behavior is consistent with rapid network formation from multivalent MoQ‐lignin and high molecular weight trithiols, followed by slower final curing as the mobility decreased and steric constraints increased progressively.

Curing yielded tough, rubbery resins with *T*_g_ values from ‐16°C to 20°C and substantial lap‐shear strengths of up to 9.1 MPa on aluminum (Figure 4c). Higher PIDA loadings increased formulation viscosity and network density, shifting failure toward adhesive modes, likely caused by reduced surface wettability (Section S5.16). This mirrors the trend found in tMoQ‐based adhesives. DSC showed comparable curing profiles across oxidation levels, with onsets at 35°C–40°C and exothermic peaks at 65°C–95°C. Peak temperatures shifted upward and total heat flow increased with PIDA loading, plateauing at ∼4 mmol·g^−1^, consistent with saturation of phenolic sites. Leaching tests with and without tributyl phosphine were identical (Section S5.17), evidencing a neglectable disulfide contribution and supporting that TGC crosslinking dominates network formation.

Interestingly, even non‐oxidized lignin reached shear strengths of 2.7, 4.2, and 5.0 MPa after curing at 60, 90, and 120°C, respectively. However, oxidation with 1 mmol·g^−1^ PIDA approximately doubled performance, yielding adhesive strength of 4.9, 7.9 and 9.1 MPa under the same curing conditions. Further increase of PIDA oxidation decreased the performance.3 This best‐performing OxL1/ETTMP_1300_ adhesive exhibited excellent moisture durability, retaining at least 80% of the dry‐reference performance under both 100% relative humidity as well as full‐immersion conditions (Section S5.17).

## 3 Conclusion

In conclusion, phenyliodine(III)diacetate (PIDA), a commodity oxidant, cleanly converts (multi)guaiacols into stable masked *ortho*‐quinone (MoQ) monomers, offering an efficient route to renewable reactive building blocks. The triguaiacol MoQ (tMoQ) undergoes Michael‐type polyaddition with multithiols in solution and forms resins under 2K bulk conditions, where competing Diels–Alder pathways are effectively suppressed. The resulting thiol‐guaiacol connectivity (TGC) polymers define hybrid materials, bridging mussel‐inspired chemistry with lignin‐derived aromatic feedstocks. Adhesion tests reveal pronounced tunability, with peak performance at intermediate network rigidity, reaching lap‐shear strengths up to 16.9 ± 0.9 MPa with TTMP at 90°C. This strategy enabled direct PIDA oxidation of lignin to produce 2K adhesives containing up to 50 wt.% lignin with shear strength of 9.1 MPa and positions MoQs as a sustainable platform for high‐performance materials.

## Author Contributions

**Dominik P. Hoch**: writing – original draft, investigation, validation, writing – review and editing, data curation.**Keven Walter**: writing – original draft, investigation, validation, writing – review and editing, data curation. **Oliver Sipura**: investigation, formal analysis. **J. Falkenhagen**: investigation, formal analysis. **X. Peng**: investigation, formal analysis. **H. Nguyen**: investigation, formal analysis, data curation. **L. Gerndt**: investigation, formal analysis, data curation, methodology. **Hans G. Börner**: conceptualization, funding acquisition, writing – review and editing, project administration, supervision, resources, formal analysis, methodology, writing – original draft.

## Conflicts of Interest

The authors declare no conflicts of interest.

## Supporting information




**Supporting File**: anie74395‐sup‐0001‐SuppMat.pdf.
Materials and instrumentations, synthesis and characterization of compounds, and materials are provided. The authors have cited additional references within the Supporting Information [66, 67, 68, 69, 70, 71, 72, 73, 74, 75, 76, 77, 78, 79, 80].

## Data Availability

The data that supports the findings of this study are available in the Supporting Information of this article.
