# Supplementary material for: Transformative Masked Ortho‐Quinone Chemistry: From Direct Guaiacol Activation to Lignin‐Based Adhesives
Source: Angew Chem Int Ed Engl. 2026 Sep 1;65(43):e5604332. doi: 10.1002/anie.5604332 (PMC13676410; doi:10.1002/anie.5604332)
Supplement: Supplementary file 1 [file ANIE-65-e5604332-s001.pdf]

Supporting information

Transformative masked *ortho*-quinone chemistry: From direct guaiacol activation to lignin-based adhesives.

Dominik P. Hoch‡, Keven Walter‡, Oliver Sipura, J. Falkenhagen, Xinyang Peng, L. Gerndt, H. Nguyen, Hans G. Börner

*D. P. Hoch+, K. Walter+, O. Sipura, X. Peng, L. Gerndt, H. Nguyen, Prof. H. G. Börner**

Humboldt-Universität zu Berlin, Department of Chemistry, Brook-Taylor Straße 2, 12489 Berlin, Germany.

J. Falkenhagen

Bundesanstalt für Materialforschung und -prüfung, BAM, Department of Materials Chemistry, Richard-Willstätter-Straße 11, 12489 Berlin, Germany

Contents

S1 Materials ... 2
S1.1 Chemicals ... 2
S1.2 Solvents ... 3
S2 Instrumentation ... 4
S3 Synthesis ... 7
S3.1 trimeric masked *ortho*-quinone (tMoQ) ... 7
S3.2 dimeric masked *ortho*-quinone (dMoQ) ... 8
S3.3 mono masked *ortho*-quinone (mMoQ) ... 9
S3.4 $mPEG_3SH$ ... 11
S3.5 TGC model compounds ... 12
S3.6 DAC model compound ... 14
S3.7 Oxidized lignin ... 15
S4 NMR-spectral characterization of novel compounds ... 16
S5 Experiments ... 24
S5.1 Analysis of PIDA mediated oxidation of tG to tMoQ ... 24
S5.2 Reactivity of tMoQ in solution ... 25
S5.3 Regioselectivity of TGC formation ... 34
S5.4 TGC polymers ... 36
S5.5 tMoQ thermal stability ... 38
S5.6 mMoQ *Diels-Alder* dimerization ... 41
S5.7 *Diels-Alder* vs TGC in bulk (mMoQ) ... 43
S5.8 Bulk reactions of tMoQ ... 45
S5.9 Preparation of 2K mixtures ... 46
S5.10 DAC formation during 2K adhesive curing ... 46
S5.11 Characterization of thiol crosslinkers for 2K application ... 47
S5.12 Rheology measurements ... 48
S5.13 Analysis of cured 2K adhesives ... 51
S5.14 Analysis of lignin ... 57
S5.15 PIDA oxidized lignin ... 58
S5.16 Lignin based 2K mixtures ... 64
S5.17 Lignin based 2K adhesives ... 67
S6 References ... 73

S1 Materials

S1.1 Chemicals

Benzhydrol (>99%, Tokyo Chemical Industry Co., LTD., Tokyo, Japan), benzaldehyde (≥99%, Merck KGaA, Darmstadt, Germany), 1-butanethiol (99%, Sigma Aldrich, St. Louis, MO, USA), 2-Chloro-4,4,5,5-tetramethyl-1,3,2-dioxaphospholane (TMDP, 95%, Sigma Aldrich, St. Louis, MO, USA), chromium(III) acetylacetonate (97%, Sigma Aldrich, St. Louis, MO, USA) ethanethiol (>98%, Tokyo Chemical Industry Co., LTD., Tokyo, Japan), ethoxylated 1,1,1-tris(hydroxymethyl)ethane tris(3-mercaptopropionate) ($ETTMP_{1300}$, THIOCURE® 333, Bruno Bock Chemische Fabrik GmbH & Co. KG, Marschacht, Germany), ethylene glycol di(mercaptopropionate) (THIOCURE® GDMP, EDMP, Bruno Bock Chemische Fabrik GmbH & Co. KG, Marschacht, Germany), dipentaerythritol-hexakis(3-mercaptopropionate) (THIOCURE®360, DHMP, Bruno Bock Chemische Fabrik GmbH & Co. KG, Marschacht, Germany), formic acid (FA, 98+%, Thermo Fisher Scientific, Darmstadt, Germany), guaiacol (98%, Tokyo Chemical Industry Co., LTD., Tokyo, Japan), hydrochloric acid (37%, Grüssing GmbH, Filsum, Germany), lignin (UPM BioPiva™ 300, UPM Biochemicals, Leuna, Germany), lithium bromide (99%, abcr GmbH, Karlsruhe, Germany) magnesium sulfate hydrate (99%, Sigma Aldrich, St. Louis, MO, USA), pentaerythritol-tetrakis(3-mercaptopropionate) (PTMP, >95%, Sigma Aldrich, St. Louis, MO, USA), pentaerythritol-tetrakis(propylene-2-mercaptoethan-1-ol) (GABEPRO™ GPM-800, Huntsman International LLC, Salt Lake City, UT, USA), phenyliodine(III) diacetate (PIDA, 98%, abcr GmbH, Karlsruhe, Germany), *p*-toluene sulfonic acid monohydrate (>98%, Sigma Aldrich, St. Louis, MO, USA), *p*-toluenesulfonyl chloride (≥98%, Sigma Aldrich, St. Louis, MO, USA), sodium bicarbonate (99%, Carl Roth GmbH + Co. KG, Karlsruhe, Germany), pyridinium trifluoroacetate (98%, Sigma Aldrich, St. Louis, MO, USA), sodium hydrosulfide (>70%, Carl Roth GmbH + Co. KG, Karlsruhe, Germany), triethylamine (99.5%, Sigma Aldrich, St. Louis, MO, USA), tributyl phosphine (99%, Sigma Aldrich, St. Louis, MO, USA), triethylene glycol monomethyl ether (>98%, Tokyo Chemical Industry Co., LTD., Tokyo, Japan), 3-(trifluoromethyl)-benzyl mercaptan (90%, Thermo Fisher Scientific, Darmstadt, Germany), triphenylphosphine oxide (98%, Sigma Aldrich, St. Louis, MO, USA), 1,1,1-tris(hydroxymethyl)ethane tris(3-mercaptopropionate) (TTMP, >95%, Sigma Aldrich, St. Louis, MO, USA), vanillin (>98%, Tokyo Chemical Industry Co., LTD., Tokyo, Japan), zinc chloride (>98%, Alfa Aesar, Haverhill, Massachusetts, USA).

Substrates for lap shear testing were purchased from Rocholl GmbH (Eschelbronn, Germany). These included aluminium, steel, polystyrene (PS) with dimension of 20 mm x 80 mm x 1.5 mm, polyamide 6 (PA6), polyamide 6.6 (PA6.6) with dimensions of 20 mm x 80 mm x 2.0 mm, polypropylene (PP), polytetrafluoroethylene (teflon), polyethylene terephthalate (PET) with dimensions of 25 mm x 80 mm x 3.0 mm as well as oak and pine wood with dimensions of 20 mm x 80 mm x 5.0 mm.

A 2K epoxy adhesive (UHU 2-K-Epoxidkleber), poly(cyanoacrylate) adhesive (Pattex Sekundenkleber Mini Trio) and a siloxane-based adhesive (Pattex Repair Extreme) were used for comparison purposes.

S1.2 Solvents

Acetonitrile (LC-MS grade, VWR International GmbH, Radnor, PA, USA), dimethylsulfoxide (DMSO, ROTIPURAN ≥99.8%, Carl Roth GmbH + Co. KG, Karlsruhe, Germany), *N*-methyl-2-pyrrolidone (NMP, peptide-grade, IRIS Biotech GmbH Marktredwitz, Germany), pyridine (SeccoSolv®, Merck KGaA, Darmstadt, Germany), tetrahydrofurane (THF, HPLC-grade, VWR International GmbH, Radnor, PA, USA) and γ-valerolactone (≥99%, BioRenewable, Sigma Aldrich, St. Louis, MO, USA) were used as received.

Acetone, dichloromethane, diethyl ether, chloroform, cyclohexane, ethyl acetate, methanol and toluene (technical grade, Stockmeier Chemie GmbH, Bielefeld, Germany) were distilled prior to use.

For NMR spectroscopy, DMSO-d_6 (D >99.8%) and $CDCl_3$ (D >99.8%) from Deutero GmbH (Kastellaun, Germany) were used.

S2 Instrumentation

Nuclear magnetic resonance spectroscopy (NMR) was carried out using an AVANCE III 500 MHz spectrometer by *Bruker BioSpin GmbH* (Rheinstetten, Germany). The chemical shifts are reported relative to tetramethylsilane in parts per million (ppm).

Ultra-high-performance liquid chromatography with electron spray ionization mass spectrometry (UHPLC-ESI-MS) was carried out using an ACQUITY UHPLC H-Class system with a photodiode array (PDA) detector and a QDa mass detector with electrospray ionization by *Waters GmbH* (Eschborn, Germany). ACQUITY BEH C18 VanGuard precolumn (110 Å, 1.7 µm, 5 x 21 mm ID) and BEH C18 column (110 Å, 1.7 µm, 5 x 21 mm ID) by *Waters GmbH* (Eschborn, Germany) were used for separation at a column temperature of 40 °C. The mobile phase was made up of mixtures of solvent A (milli-Q water + 0.1% FA) and solvent B (acetonitrile + 0.1% FA) with a flow rate of 0.5 mL/min.

Single crystal X-ray (SC XRD) structure determinations were performed on a BRUKER D8 VENTURE diffractometer at 100 K by using Mo Kα radiation (λ = 0.71073 Å).Data collections were performed The structure was solved with the XT[1] solution program using Intrinsic Phasing methods and by using Olex2 1.5[2] as the graphical interface. The model was refined with XL using full matrix least squares minimization on F2.

Gel permeation chromatography (GPC):

THF-GPC analysis was carried out using an EcoSEC HLC8320 GPC system with UV and RI detectors by *Tosoh Bioscience GmbH* (Griesheim, Germany). Tetrahydrofurane (HiPerSolv CHROMANORM for HPLC) by *VWR® Chemicals* (Dresden, Germany) was used as the eluent and SDV columns (1000 A, 5 µm, 100000 A, 5 µm, 1000000 A, 5 µm) by *PSS* (Mainz, Germany) were used for separation. The calibration was done using polystyrene calibration standards between 1480 g/mol and 3040000 g/mol by *PSS* (Mainz, Germany) and *Varian Inc.* (Palo Alto, California, USA). A sample concentration of 1 mg/mL and an injection volume of 40 yL were used alongside BHT by *Sigma Aldrich* (Taufkirchen, Germany) as an internal standard.

DMSO-GPC analysis was performed using a GPC system equipped with WinGPC UniChrom 8.2 software by *PSS* (Mainz, Germany). The system comprised an isocratic pump (HPLC Compact Pump 3350, Bischoff), a thermostated column compartment operated at 60 °C, a refractive index detector (Shodex RI-501) and a photodiode array detector (SPD-M20A, Shimadzu) operated at 280 nm. DMSO containing 0.075 M $NaNO_3$ was used as the eluent at a flow rate of 0.5 mL/min. Separation was achieved using a precolumn (ABOA DMSO-Phil-P-250, 8 × 50 mm, 10^2 to 7 × 10^4 g/mol, AppliChrom), followed by two DMSO-Phil-P-250 columns (8 × 300 mm, 10^2 to 7 × 10^4 g/mol, AppliChrom) and a DMSO-Phil-P-Multipore column (15 µm, 8 × 300 mm, 10^2 to 10^6 g/mol, AppliChrom). Calibration was performed using ten pullulan standards by *PSS* (Mainz, Germany). Samples (2 mg/mL) were filtered through 0.2 µm syringe filters prior to injection.

Fourier transform infrared spectroscopy (FTIR) was performed using a Brucker Vertex 70v FT-IR spectrometer by *Brucker Optik GmbH* (Ettlingen, Germany).

UV/vis spectroscopy was performed using a UV-2501PC spectrometer by *Shimadzu Deutschland GmbH* (Duisburg, Germany).

Flash column chromatography was carried out automatically using Combi*Flash*® Rf+ device with UV detection by *Teledyne Isco Inc.* (Nebraska, USA). RediSep-Rf columns (silica, 40 - 60 µm particle size) by *Axel Semrau GmbH & Co. KG* (Sprockhövel, Germany) were used for separation.

Lap shear tests were performed on a Texture Analyser TA.XT *plus*100*C* with a 100 kg force cell by *Stable Micro Systems* (Godalming, United Kingdom) with a velocity of 0.05 mm/s using *Exponent Connect* software.

For 2K adhesive sample preparation a DAC 150 SP SpeedMixer® by *Hauschild GmbH & Co. KG* (Hamm, Germany) with a maximum of 3500 revolutions per minute and a maximal loading of 150 g was used.

Rheology measurements were carried out on a MCR 302e (Anton Paar GmbH, Graz, Austria) rheometer with a plate/plate (PP) measuring geometries with disposable-plate measuring system and gap width of 0.5 mm.

Thermal gravimetric analysis (TGA) measurements were carried out on a Thermogravimetric Analyzer Pyris 1 (Perkin Elmer, Waltham, USA). Approximatively 10 mg of each sample were weighed in a ceramic pan and heated from 30 °C up to 800 °C at a heating rate of 20 $°C \cdot min^{-1}$, under argon atmosphere with a flow rate of 20 $mL \cdot min^{-1}$.

Differential scanning calorimetry (DSC) analysis was performed on a DSC 3 (Mettler-Toledo GmbH, Gießen, Germany) connected to a cooler TC100 (Peter Huber Kältemaschinenbau SE, Offenburg, Germany). All measurements were performed under an argon atmosphere with a heating rate of 10 $°C \cdot min^{-1}$.

DFT Calculations. All calculations in this work were performed utilizing the ORCA program package version 6.[3] For the investigation of the reaction pathway, initial transition state geometries were optimized at the DFT level employing the BP86 functional2[4] augmented with the D4 dispersion[5] correction and the def2-SVPD[6] basis set, where the addition of diffuse functions is warranted by the nature of the investigated proton transfer reactions. The AutoAux[7] procedure was invoked to fit auxiliary basis sets, and the RIJCOSX[8] approximation was employed to accelerate calculations. Solvent effects were accounted for using the implicit conductor-like polarizable continuum model CPCM[9] with methanol as solvent. The keyword TightSCF was applied to ensure tight convergence, and harmonic frequency calculations were performed at the same level of theory to confirm all optimized transition state structures as first-order saddle points.

For improved accuracy, transition state optimizations were subsequently performed utilizing the B3LYP functional[6] augmented with the D4 dispersion correction, the def2-TZVPD[6] basis set for all atoms, and the def2/J[10] auxiliary basis set, employing the RIJCOSX approximation and CPCM for methanol. Harmonic frequency calculations were performed at the same level of theory as the corresponding

geometry optimization to confirm all optimized transition state structures as first-order saddle points and to derive thermochemical corrections at 298.15 K and 1 atm.

Intrinsic reaction coordinate (IRC)[11] calculations were carried out at the same level of theory to confirm the connectivity of each transition state to the corresponding reactant and product structures. The resulting endpoints were fully re-optimized applying the TightOpt and TightSCF convergence criteria, and subsequent frequency calculations confirmed all stationary points as true local minima with no significant imaginary frequencies.

Final single-point energies were obtained at the DLPNO-CCSD(T)[12] level applying the TightSCF keyword, the AutoAux procedure, the RIJCOSX approximation, and CPCM for methanol. To extrapolate to the complete basis set (CBS) limit, calculations with the cc-pVDZ[13] and cc-pVTZ13[13] basis sets were performed.

S3 Synthesis

S3.1 trimeric masked *ortho*-quinone (tMoQ)

tMoQ was prepared in a two-step synthesis starting with the condensation of vanillin and guaiacol to obtain tG followed by a PIDA-mediated oxidation. tG was synthesized according to Liu *et. al*.[14]

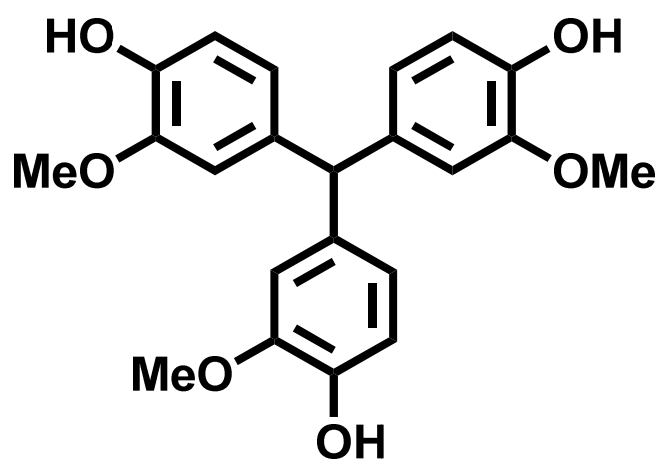

Vanillin (50.0 g, 329 mmol, 1 eq) was suspended in guaiacol (204 g, 1640 mmol, 5 eq) and zinc chloride (4.48 g, 32.9 mmol, 0.1 eq) and *para*-toluene sulfonic acid monohydrate (6.25 g, 32.9 mmol, 0.1 eq) were added. The mixture was stirred at 50 °C for 5 days before it was precipitated into chloroform (2000 mL). The precipitate was collected via filtration and washed with an aqueous methanol solution (5 × 100 mL, 10%). The crude product was recrystallized from chloroform and dried under reduced pressure at 110 °C, yielding tG as a pink powder (47.9 g, 125 mmol, 38%).

^{1}H NMR (DMSO-d_6, 500 MHz): δ [ppm] = 8.80 (s, 3H), 6.81 – 6.62 (m, 6H), 6.48 (dd, J=8.2, 1.8, 3H), 5.27 (s, 1H), 3.66 (s, 9H).

^{13}C NMR (DMSO-d_6, 126 MHz): δ [ppm] = 147.2, 144.7, 135.7, 121.2, 115.1, 113.3, 79.2, 55.6, 54.7.

UHPLC-ESI-MS t_R = 2.31 min (λ = 280 nm, gradient 10-90% B with A: H2O + 0.1% FA and B: ACN + 0.1% FA); found (m/z): 259.36 [M+H-guaiacol]$^+$, calculated (m/z): 259.10 [M+H-guaiacol]$^+$.

FTIR (ATR): $\tilde{\nu}$ [cm^{-1}] = 3387 (s), 2977 (s), 2859 (m), 1614 (w), 1503 (s), 1452 (m), 1427 (m), 1370 (w) 1264 (m), 1240 (m), 1200 (s), 1148 (m), 1120 (m), 1034 (m), 749 (m), 639 (w), 551 (w).

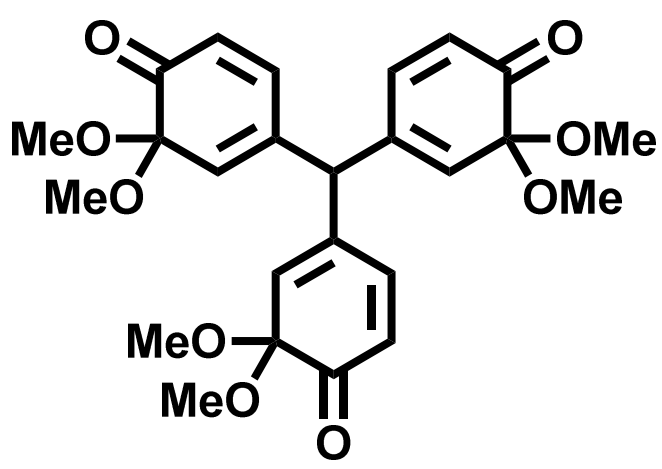

tG (20.0 g, 52.3 mmol, 1 eq) was dissolved in MeOH (400 mL) and PIDA (53.1 g, 164.9 mmol, 3.15 eq) was added. After stirring at room temperature for 15 min the MeOH was removed under reduced pressure. The resulting red oil was diluted with 400 mL ethyl acetate and washed with a $NaHCO_3$ solution (400 mL, 13.1 g, 156 mmol, 3 eq) and brine (400 mL). After drying the organic phase over $MgSO_4$ the solvent was removed under reduced pressure and the obtained oil was precipitated into 1000 mL cold cyclohexane. The yellow solid was collected via filtration, washed with cyclohexane (3 × 100 mL) and dried under vacuum to obtain tMoQ (19.9 g, 42.2 mmol, 81%).

^{1}H NMR ($CDCl_3$, 500 MHz) δ [ppm] = 6.68 (dd, *J* = 10.0, 2.3 Hz, 3H), 6.14 (t, *J* = 1.5 Hz, 3H), 6.10 (d, *J* = 9.9, 3H), 3.92 (s, 1H), 3.35 (s, 18H).

^{13}C NMR ($CDCl_3$, 126 MHz) δ [ppm] = 194.2, 140.2, 134.6, 134.2, 126.8, 91.4, 54.1, 50.2.

UHPLC-ESI-MS t_R = 2.55 min (λ = 280 nm, gradient 10-90% B with A: H2O + 0.1% FA and B: ACN + 0.1% FA); found (m/z): 471.26 $[M-H]^-$, calculated (m/z): 471.17 $[M-H]^-$.

FTIR (ATR): $\tilde{\nu}$ [cm^{-1}] = 2942 (m), 2828 (w), 1681 (s), 1651 (m), 1467 (w), 1399 (w), 1255 (w), 1130 (w), 1094 (m), 1060 (m), 1023 (s), 953 (m), 819 (m).

S3.2 dimeric masked *ortho*-quinone (dMoQ)

dMoQ was prepared in a two-step synthesis starting with the condensation of benzaldehyde and guaiacol to obtain dG followed by a PIDA-mediated oxidation.

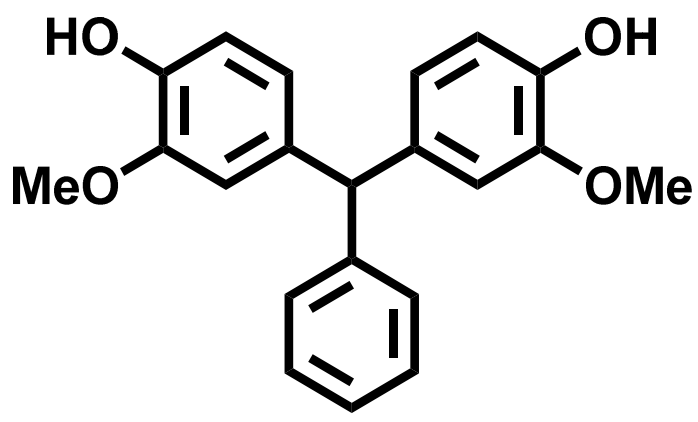

Benzaldehyde (15.0 g, 141 mmol, 1 eq) was suspended in guaiacol (87.7 g, 707 mmol, 5 eq) before zinc chloride (1.93 g, 14.1 mmol, 0.1 eq) and *para*-toluene sulfonic acid monohydrate (2.69 g, 14.1 mmol, 0.1 eq.) were added. The mixture was stirred at 50 °C for 5 d before it was precipitated into water (1500 mL). The resulting oil was dried under reduced pressure, dissolved in MeOH/acetone/H_2O (250 mL, 3/1/1) and allowed to crystallize for 3 days. The obtained solids were collected via filtration, washed with an aqueous methanol solution (2x 100 mL, 50%) and dried under reduced pressure to yield mG (15.6 g, 46.3 mmol, 33%) as a white solid.

^{1}H NMR (DMSO-d_6, 500 MHz) δ [ppm] = 7.33 – 7.28 (m, 2H), 7.25 – 7.20 (m, 1H), 7.16 – 7.12 (m, 2H), 6.85 (d, *J* = 8.1 Hz, 2H), 6.66 (d, *J* = 2.0 Hz, 2H), 6.59 (dd, *J* = 8.1, 2.1, 2H), 5.44 (s, 1H), 3.78 (s, 6H).

^{13}C NMR (DMSO-d_6, 126 MHz): δ [ppm] = 146.5, 144.6, 144.1, 136.3, 129.4, 128.4, 126.3, 122.3, 114.2, 112.1, 56.2, 56.0.

UHPLC-ESI-MS t_R = 2.94 min (λ = 280 nm, gradient 10-90% B with A: H2O + 0.1% FA and B: ACN + 0.1% FA); found (m/z): 213.33 $[M+H\text{-guaiacol}]^+$, calculated (m/z): 213.26 $[M+H\text{-guaiacol}]^+$.

FTIR (ATR): $\tilde{\nu}$ [cm^{-1}] = 3346 (s), 2985 (s), 2876 (m), 1604 (m), 1512 (s), 1458 (m), 1423 (m), 1357 (w), 1269 (s), 1197 (m), 1151 (m), 1113 (m), 1030 (m), 811 (w), 689 (m), 656 (w).

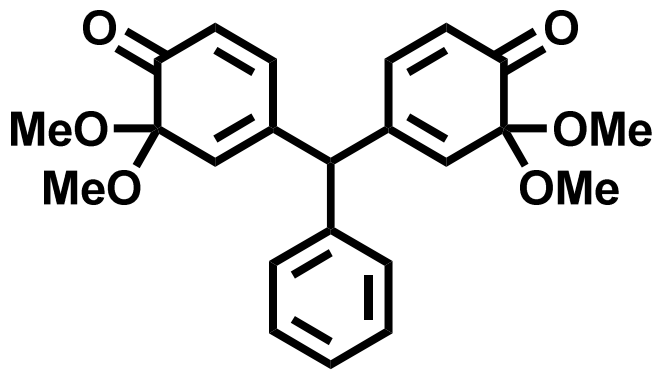

dG (1.50 g, 4.46 mmol, 1 eq) was dissolved in MeOH (30 mL, 50 mg/mL) and PIDA (3.02 g, 9.36 mmol, 2.1 eq) was added. After stirring at room temperature for 15 min 100 mL of MQ water was added and the product was extracted using DCM. After drying over $MgSO_4$ the solvent was removed under reduced pressure. The resulting oil was purified via column chromatography (silica, 0 to 50% EtOAc in cyclohexane) and allowed to solidify, before being dried under reduced pressure to obtain dMoQ (1.57 g, 3.96 mmol, 89%) as a yellow powder.

^{1}H NMR (DMSO-d_6, 500 MHz) δ [ppm] = 7.46 – 7.41 (m, 2H), 7.37 – 7.32 (m, 1H), 7.25 – 7.21 (m, 2H), 6.87 (dd, *J* = 10.1, 2.3 Hz, 2H), 6.06 (d, *J* = 9.9 Hz, 2H), 5.87 (t, *J* = 1.6 Hz, 2H), 4.86 (s, 1H), 3.21 (s, 6H), 3.20 (s, 6H).

^{13}C NMR (DMSO-d_6, 126 MHz): δ [ppm] = 194.8, 141.7, 137.3, 136.7, 133.5, 129.4, 128.2, 126.1, 91.6, 76.9, 55.2, 50.2.

UHPLC-ESI-MS t_R = 3.15 min (λ = 280 nm, gradient 10-90% B with A: H2O + 0.1% FA and B: ACN + 0.1% FA); found (m/z): 365.42 $[M+H-MeOH]^+$, calculated (m/z): 365.14 $[M+H-MeOH]^+$.

FTIR (ATR): $\tilde{\nu}$ [cm^{-1}] = 2982 (s), 2866 (m), 1738 (w), 1679 (s), 1652 (m), 1449 (w), 1401 (w), 1246 (w), 1120 (m), 1076 (s), 1028 (s), 961 (m), 821 (w), 702 (m).

S3.3 mono masked *ortho*-quinone (mMoQ)

mMoQ was prepared in a two-step synthesis starting with the condensation of benzhydrol and guaiacol to obtain mG followed by a PIDA-mediated oxidation.

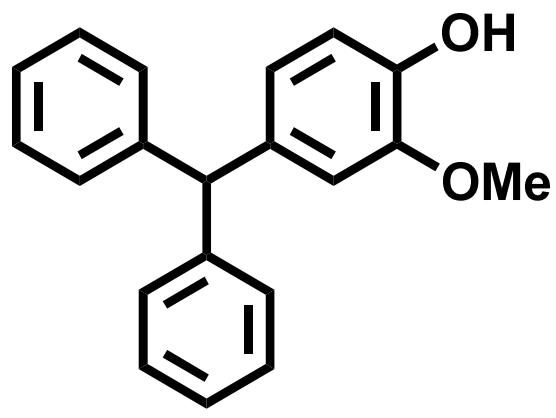

Benzhydrol (25.4 g, 138 mmol, 1 eq) was suspended in guaiacol (39.7 g, 276 mmol, 2 eq) before zinc chloride (0.94 g, 6.9 mmol, 0.05 eq.) and *para*-toluene sulfonic acid monohydrate (1.31 g, 6.9 mmol, 0.05 eq.) were added. The mixture was stirred at 50 °C for 5 d before it was precipitated into water (500 mL). The resulting oil was dried under reduced pressure, dissolved in MeOH/H_2O (120 mL, 5/1) and allowed to crystallize for 3 days. The obtained solids were collected via filtration, washed with an aqueous methanol solution (2x 25 mL, 50%) and dried under reduced pressure to yield mG (8.3 g, 28.6 mmol, 35%) as a white solid.

¹H NMR (DMSO-d_6, 500 MHz) δ [ppm] = 8.88 (s, 1H), 7.37 – 7.24 (m, 4H), 7.20 (d, *J* = 7.3 Hz, 2H), 7.14 – 7.06 (m, 4H), 6.72 (d, *J* = 8.1 Hz, 1H), 6.70 (d, *J* = 2.0 Hz, 1H), 6.47 (dd, *J* = 8.2, 2.0 Hz, 1H), 5.49 (s, 1H), 3.64 (s, 3H).

¹³C NMR (DMSO-d_6, 126 MHz): δ [ppm] = 147.4, 145.0, 144.3, 134.6, 129.0, 128.2, 126.1, 121.4, 115.3, 113.4, 55.6, 55.4, 40.0, 39.9, 39.7, 39.5, 39.4, 39.2, 39.0.

UHPLC-ESI-MS t_R = 3.69 min (λ = 280 nm, gradient 10-90% B with A: H2O + 0.1% FA and B: ACN + 0.1% FA); found (m/z): 167.25 $[M+H\text{-guaiacol}]^+$, calculated (m/z): 167.09 $[M+H\text{-guaiacol}]^+$.

FTIR (ATR): $\tilde{\nu}$ [cm^{-1}] = 3514 (s), 2986 (m), 1600 (m), 1506 (s), 1450 (m), 1350 (w), 1273 (s), 1226 (s), 1136 (m), 1027 (s), 945 (w), 822 (w), 698 (s).

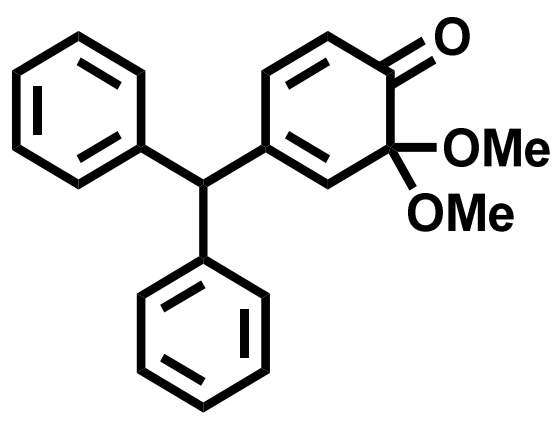

mG (4.00 g, 13.8 mmol, 1 eq) was dissolved in MeOH (80 mL, 50 mg/mL) and PIDA (4.66 g, 14.5 mmol, 1.05 eq) was added. After stirring at room temperature for 15 min 200 mL of MQ water was added and the product was extracted using DCM. After drying over $MgSO_4$ the solvent was removed under reduced pressure. The resulting oil was purified via column chromatography (silica, 0 to 20% EtOAc in cyclohexane) and allowed to solidify, before being dried under reduced pressure to obtain mMoQ (3.71 g, 11.6 mmol, 84%) as a yellow powder.

¹H NMR (DMSO-d_6, 500 MHz) δ [ppm] = 7.42 – 7.33 (m, 4H), 7.32 – 7.24 (m, 2H), 7.23 – 7.15 (m, 4H), 6.80 (dd, *J* = 10.1, 2.3 Hz, 1H), 6.00 (d, *J* = 9.9 Hz, 1H), 5.73 (d, *J* = 1.9 Hz, 1H), 5.23 (s, 1H), 3.18 (s, 6H).

¹³C NMR (DMSO-d_6, 126 MHz): δ [ppm] = 195.1, 143.0, 140.5, 140.2, 132.2, 129.0, 128.7, 126.9, 125.1, 91.2, 54.6, 49.6.

UHPLC-ESI-MS t_R = 3.72 min (λ = 280 nm, gradient 10-90% B with A: H2O + 0.1% FA and B: ACN + 0.1% FA); found (m/z): 289.30 $[M+H\text{-MeOH}]^+$, calculated (m/z): 289.12 $[M+H\text{-MeOH}]^+$.

FTIR (ATR): $\tilde{\nu}$ [cm^{-1}] = 2970 (m), 1687 (s), 1581 (w), 1495 (m), 1450 (m), 1400 (m), 1251 (m), 1081 (s), 1033 (s), 958 (m), 813 (m), 748 (m), 700 (s).

S3.4 mPEG$_3$SH

mPEG$_3$SH was prepared in a two-step synthesis starting with the tosylation of triethylene glycol monomethyl ether followed by nucleophilic substitution using sodium hydrosulfide.

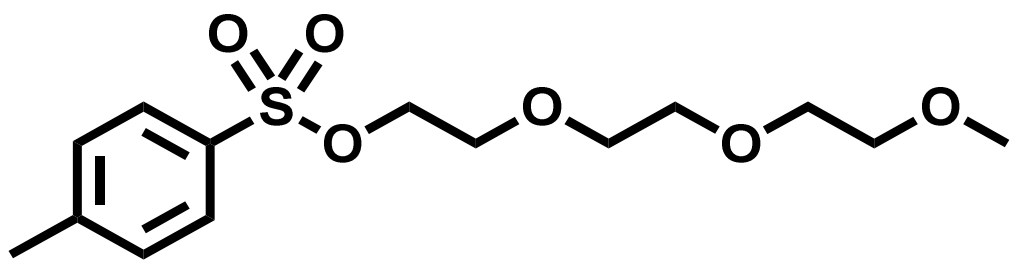

The synthesis was adapted from a previously described procedure.[15]

Triethylene glycol monomethyl ether (55.0 g, 335 mmol, 1 eq) and *p*-toluenesulfonyl chloride (70.3 g, 369 mmol, 1.1 eq) were dissolved in DCM (400 mL). Triethylamine (102 mL, 737 mmol, 2.2 eq) was added and the solution was stirred at RT for three hours, before the resulting suspension was neutralized with concentrated HCl (12M, 61.4 mL). The organic phase was washed with a saturated $NaHCO_3$ solution three times, dried over $MgSO_4$ and the solvent was removed under reduced pressure to obtain TsO-PEG$_3$-OMe (111 g, quantitative) as a colorless oil, which was used without further purification in the next reaction.

^{1}H NMR (DMSO-d$_6$, 500 MHz) δ [ppm] = 7.86 – 7.73 (m, 1H), 7.36 – 7.31 (m, 1H), 4.18 – 4.12 (m, 1H), 3.71 – 3.66 (m, 1H), 3.63 – 3.56 (m, 3H), 3.56 – 3.48 (m, 1H), 3.37 (s, 1H), 2.44 (s, 1H).

^{13}C NMR (DMSO-d$_6$, 126 MHz): δ [ppm] = 144.9, 133.1, 130.4, 129.9, 128.1, 127.2, 77.4, 77.2, 76.9, 72.0, 70.9, 70.7, 70.7, 69.4, 68.8, 59.2, 21.8.

UHPLC-ESI-MS t_R = 3.25 min (λ = 280 nm, gradient 10-90% B with A: H2O + 0.1% FA and B: ACN + 0.1% FA); The compound could not be ionized by ESI-MS.

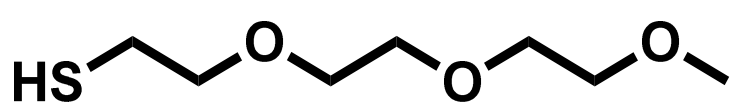

The synthesis was adapted from a previously described procedure.[16]

TsO-PEG$_3$-OMe (111 g, 335 mmol, 1 eq) was dissolved in MeOH (300 mL) and degassed with argon. Sodium hydrosulfide (93.9 g, 1.68 mol, 5 eq) was dissolved in water (300 mL) and degassed with argon. The MeOH solution was added to this aqueous solution over the course of 30 minutes under an argon atmosphere and the resulting mixture was stirred at RT overnight. The solution was acidified using concentrated HCl (12 M, 140 mL) and extracted with DCM three times. The combined organic phase was dried over $MgSO_4$ and the solvent removed under reduced pressure. The crude product was further purified by vacuum distillation to obtain mPEG$_3$SH (29.8 g, 165 mmol, 49%) as a colorless oil.

^{1}H NMR ($CDCl_3$, 500 MHz) δ [ppm] = 3.65 – 3.52 (m, 10H), 3.36 (s, 3H), 2.68 (dt, *J* = 8.2, 6.5 Hz, 2H), 1.57 (t, *J* = 8.2 Hz, 1H).

^{13}C NMR ($CDCl_3$, 126 MHz) δ [ppm] = 73.0, 72.0, 70.7, 70.7, 70.3, 59.2, 24.4.

FTIR (ATR): $\tilde{\nu}$ [cm^{-1}] = 3330 (w), 2873 (m), 1659 (w), 1469 (w), 1405 (w), 1351 (w), 1279 (w), 1195 (w), 1100 (s), 1023 (w), 841 (w), 617 (w).

S3.5 TGC model compounds

mMoQ butane thiol TGC-adduct

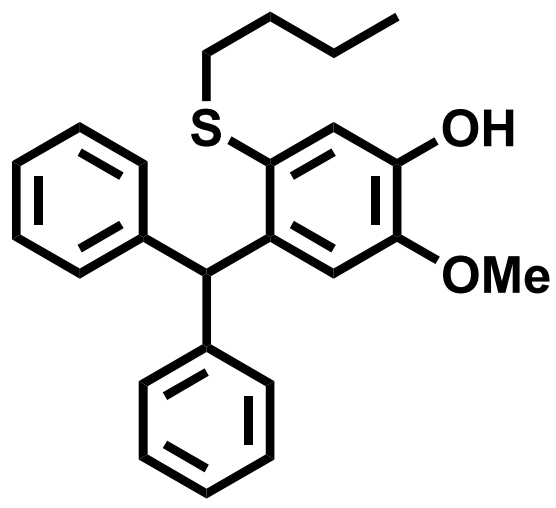

mMoQ (160 mg, 0.50 mmol, 1 eq) was dissolved in 5 mL MeOH and butane thiol (451 mg, 5.0 mmol, 10 eq) was added. After stirring for 16 hours the solution was evaporated using a constant flow of argon and the resulting yellow oil was dissolved in THF, precipitated into an aqueous methanol solution (3x 10 mL, 50%) and recovered by centrifugation. The product was dissolved in acetonitrile and freeze dried to remove any leftover solvent to obtain the TGC adduct (183 mg, 0.48 mmol, 97%) as a yellow oil.

^{1}H NMR (DMSO-d$_6$, 500 MHz) δ [ppm] = 9.19 (s, 1H), 7.31 – 7.26 (m, 4H), 7.22 – 7.17 (m, 2H), 7.07 – 7.03 (m, 4H), 6.93 (s, 1H), 6.41 (s, 1H), 6.07 (s, 1H), 3.51 (s, 3H), 2.60 (t, *J* = 7.2 Hz, 3H), 1.41 – 1.33 (m, 2H), 1.25 (dt, *J* = 8.0, 6.8 Hz, 2H), 0.77 (t, *J* = 7.3 Hz, 2H).

^{13}C NMR (DMSO-d$_6$, 126 MHz): δ [ppm] = 146.7, 145.4, 143.8, 135.8, 129.1, 128.3, 126.2, 125.9, 119.1, 114.0, 55.6, 52.7, 34.4, 30.8, 21.3, 13.5.

UHPLC-ESI-MS t_R = 3.37 min (λ = 280 nm, gradient 50-95% B with A: H2O + 0.1% FA and B: ACN + 0.1% FA); found (m/z): 379.42 $[M+H]^+$, calculated (m/z): 379.17 $[M+H]^+$.

FTIR (ATR): $\tilde{\nu}$ [cm^{-1}] = 3509 (w), 2955 (m), 2929 (m), 1602 (w), 1577 (w), 1495 (s) 1447 (m), 1411 (w), 1267 (m), 1198 (m), 1047 (w), 863 (w), 698 (m).

mMoQ ethane thiol TGC-adduct

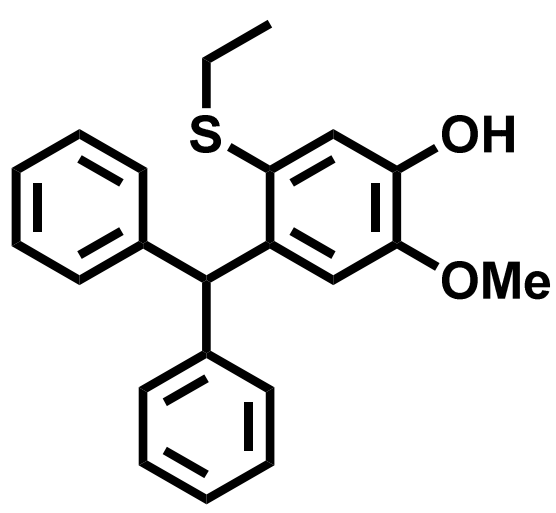

mMoQ (2000 mg, 6.24 mmol, 1 eq) was dissolved in 19 mL MeOH and ethane thiol (1940 mg, 31.2 mmol, 5 eq) was added. After stirring for 2 hours the solution was evaporated using a constant flow of argon and the resulting yellow oil was dissolved in THF, precipitated into an aqueous methanol solution (3x 50 mL, 10%) and recovered by centrifugation. The product was dissolved in acetonitrile and freeze dried to remove any leftover solvent to obtain the TGC adduct (2060 mg, 5.89 mmol, 94%) as a white solid. The product was further recrystallized from methanol.

^{1}H NMR (DMSO-d$_6$, 500 MHz) δ [ppm] = 9.17 (s, 1H), 7.31 – 7.27 (m, 4H), 7.23 – 7.17 (m, 2H), 7.08 – 7.02 (m, 4H), 6.92 (s, 1H), 6.41 (s, 1H), 6.05 (s, 1H), 2.65 (q, *J* = 7.3 Hz, 2H), 1.07 (t, *J* = 7.3 Hz, 3H).

^{13}C NMR (DMSO-d$_6$, 126 MHz): δ [ppm] = 146.6, 145.4, 143.8, 135.7, 129.1, 128.3, 126.2, 125.7, 118.9, 114.0, 55.6, 52.7, 28.6, 14.3.

UHPLC-ESI-MS t_R = 3.51 min (λ = 254 nm, gradient 50-95% B with A: H2O + 0.1% FA and B: ACN + 0.1% FA); found (m/z): 351.40 $[M+H]^+$, calculated (m/z): 351.13 $[M+H]^+$.

FTIR (ATR): $\tilde{\nu}$ [cm^{-1}] = 3511 (w), 2934 (w), 2928 (w), 1597 (w), 1579 (w), 1495 (s) 1432 (m), 1411 (w), 1271 (m), 1203 (m), 1048 (w), 849 (w), 696 (m).

Crystallographic data for the mMoQ ethane thiol TGC-adduct have been deposited with the Cambridge Crystallographic Data Centre under deposition number CCDC 2560822

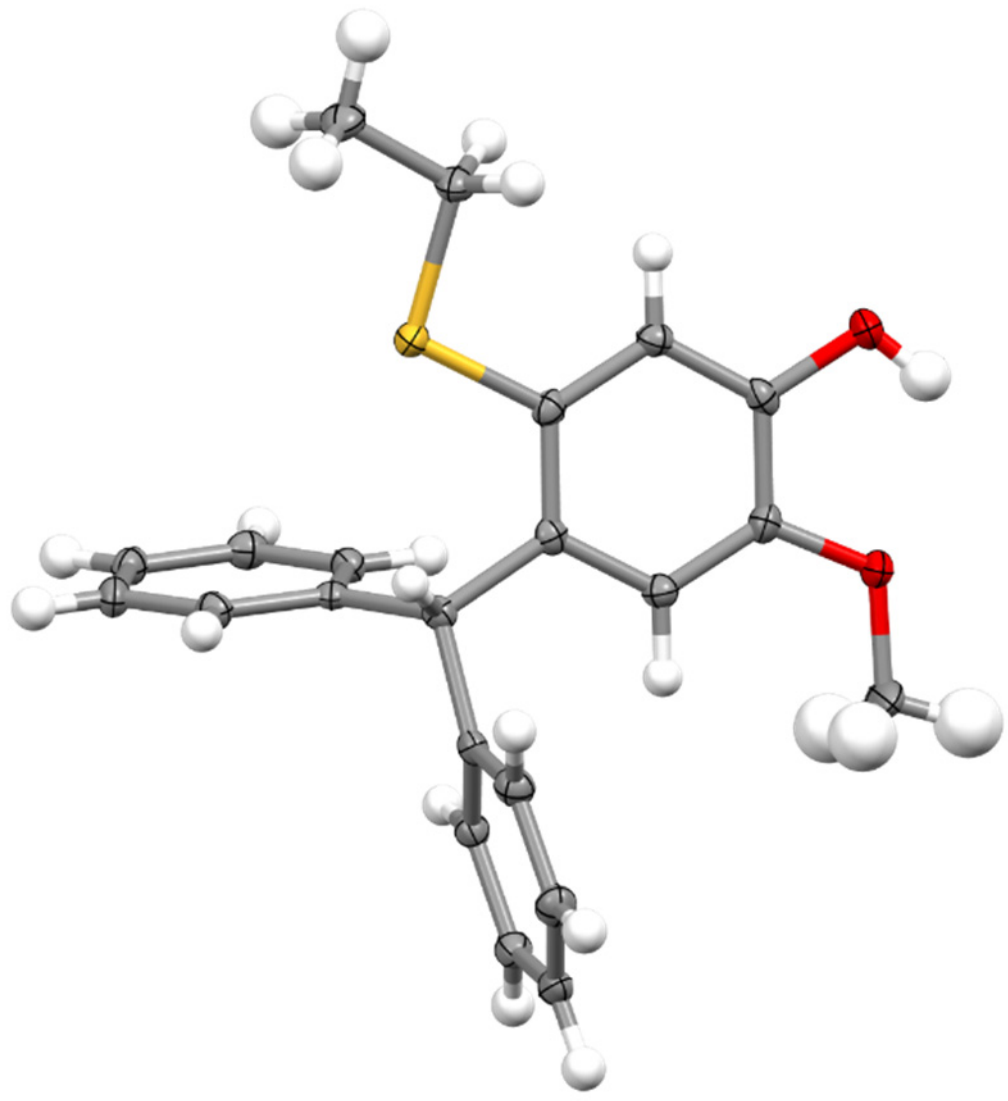

Figure S 1. Molecular structure of the mMoQ ethane thiol TGC-adduct determined by single-crystal X-ray diffraction. Carbon atoms are shown in grey, oxygen atoms in red, sulfur atoms in yellow and hydrogen atoms in white. Thermal ellipsoids are drawn at the 50% probability level.

Table S 1. Crystallographic data for the mMoQ ethane thiol TGC-adduct.

**CCDC number**	2560822
**Empirical formula**	$C_{22}H_{22}O_2S$
**Formula weight**	350.45
**Temperature [K]**	100.00
**Wavelength [Å]**	0.71073
**Crystal system/space group**	monoclinic, C2/c
**Crystal size [mm³]**	0.07 × 0.03 × 0.02
**a [Å]**	23.8559(11)
**b [Å]**	10.2273(5)
**c [Å]**	18.9136(13)
**α [°]**	90
**β [°]**	128.1200(10)
**γ [°]**	90
**Volume [Å]**	3630.4(4)
**Z**	8
**ρ_{calc} [g/cm³]**	1.282
**μ/mm⁻¹**	0.190
**F(000)**	1488.0
**Data/restraints/parameters**	3724/0/229
**Goodness-of-fit on F^2**	1.049
**Final R indexes [all data]**	R_1 = 0.0500, wR_2 = 0.0920

S3.6 DAC model compound

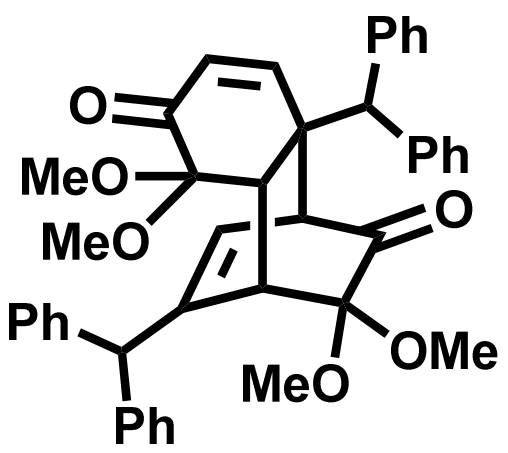

mMoQ (1.50 g, 4.7 mmol) was heated to 90 °C for 6 days. Column chromatography (silica, 0% to 15% EtOAc in cyclohexane) was performed to obtain the product as a yellow powder (0.78 g ,1.2 mmol, 52%).

^{1}H NMR (500 MHz, Chloroform-*d*): δ [ppm] = 7.51 – 7.19 (m, 20H), 6.90 (d, *J* = 10.5 Hz, 1H), 6.06 (d, *J* = 10.5 Hz, 1H), 5.33 (dt, *J* = 7.2, 2.1 Hz, 1H), 4.62 (d, *J* = 2.1 Hz, 1H), 4.24 (s, 1H), 3.52 (s, 3H), 3.27 (d, *J* = 2.3 Hz, 1H), 3.23 (s, 3H), 3.07 (d, *J* = 7.2 Hz, 1H), 3.06 (t, *J* = 2.4 Hz, 1H), 2.72 (s, 3H), 2.49 (s, 3H).

^{13}C NMR (126 MHz, Chloroform-*d*): δ [ppm] = 200.9, 192.6, 150.1, 148.8, 141.2, 140.6, 140.5, 139.3, 130.8, 130.7, 130.4, 129.5, 128.5, 128.3, 127.5, 127.2, 126.9, 126.8, 126.5, 125.7, 97.1, 94.3, 61.4, 57.5, 56.7, 53.0, 50.5, 49.1, 48.9, 48.5, 45.5, 44.4, 27.0.

FTIR (ATR): $\tilde{\nu}$ [cm^{-1}] = 3681 (w), 2964 (s), 1737 (s), 1688 (s), 1600 (w), 1495 (m), 1452 (m), 1394 (w), 1248 (w), 1145 (s), 1058 (s), 979 (m), 698 (s).

UHPLC-ESI-MS t_R = 3.48 min (λ = 254 nm, gradient 50-90% B with A: H2O + 0.1% FA and B: ACN + 0.1% FA); The compound could not be ionized by ESI-MS.

S3.7 Oxidized lignin

The technical softwood kraft lignin (UPM BioPiva™ 300, UPM Biochemicals, Leuna, Germany) was fractionated to remove insoluble material prior to PIDA oxidation.
The lignin (50.0 g, 35% moisture content) was added to acetone (500 mL), stirred for 1 h and filtered. The filtrate was concentrated under reduced pressure to obtain the acetone soluble lignin fraction (26.9 g, 83 wt% based on lignin dry mass).

^{31}P-NMR: 3.58 ±0.05 mmol/g phenolic OH and 2.06 ± 0.02 mmol/g aliphatic OH

GPC (DMSO + 0.075 M $NaNO_3$): M_n = 1700 g/mol, M_w = 6500 g/mol, $Đ$ = 3.9

FTIR (ATR): $\tilde{\nu}$ [cm^{-1}] = 3401 (m), 2981 (s), 2873 (m), 1704 (w), 1596 (m), 1512 (m), 1452 (m), 1423 (m), 1362 (w), 1266 (s), 1212 (s), 1131 (m), 1083 (m), 1026 (m).

The acetone soluble lignin (5.0 g) was dissolved in γ-valerolactone/MeOH (2/1, 50 mL) and stirred at room temperature in a water bath. The desired amount of PIDA (**Table S 2**) was added and the mixture was stirred for 15 min before precipitating in 500 mL of Et_2O. The solid was collected and washed with Et_2O (3 × 50 mL) and water (3 × 50 mL). Lyophilization afforded the PIDA oxidized lignin (67 wt% to 79 wt%) as a light brown solid.

Table S 2. Amount of PIDA (mmol/g, molar amount and weight) as well as yield (wt%) for the oxidation of acetone soluble lignin.

Amount of PIDA [mmol/g]	Amount of PIDA [mmol]	Amount of PIDA [g]	Yield
0	0	0	67 wt%
1	5	1.61	70 wt%
2	10	3.22	68 wt%
3	15	4.83	72 wt%
4	20	6.44	79 wt%

S4 NMR-spectral characterization of novel compounds

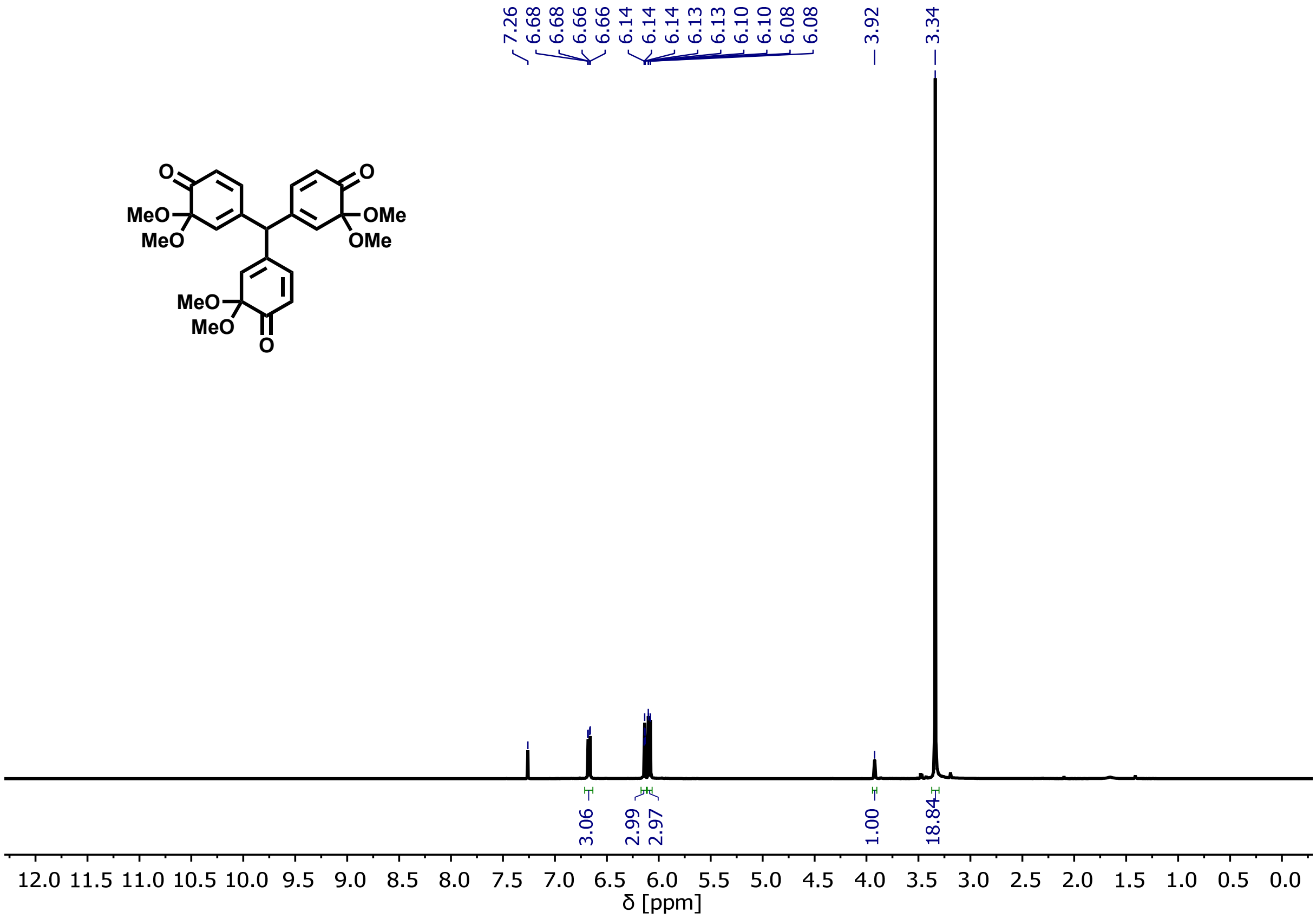

Figure S 2. ^{1}H-NMR spectrum of tMoQ in $CDCl_3$.

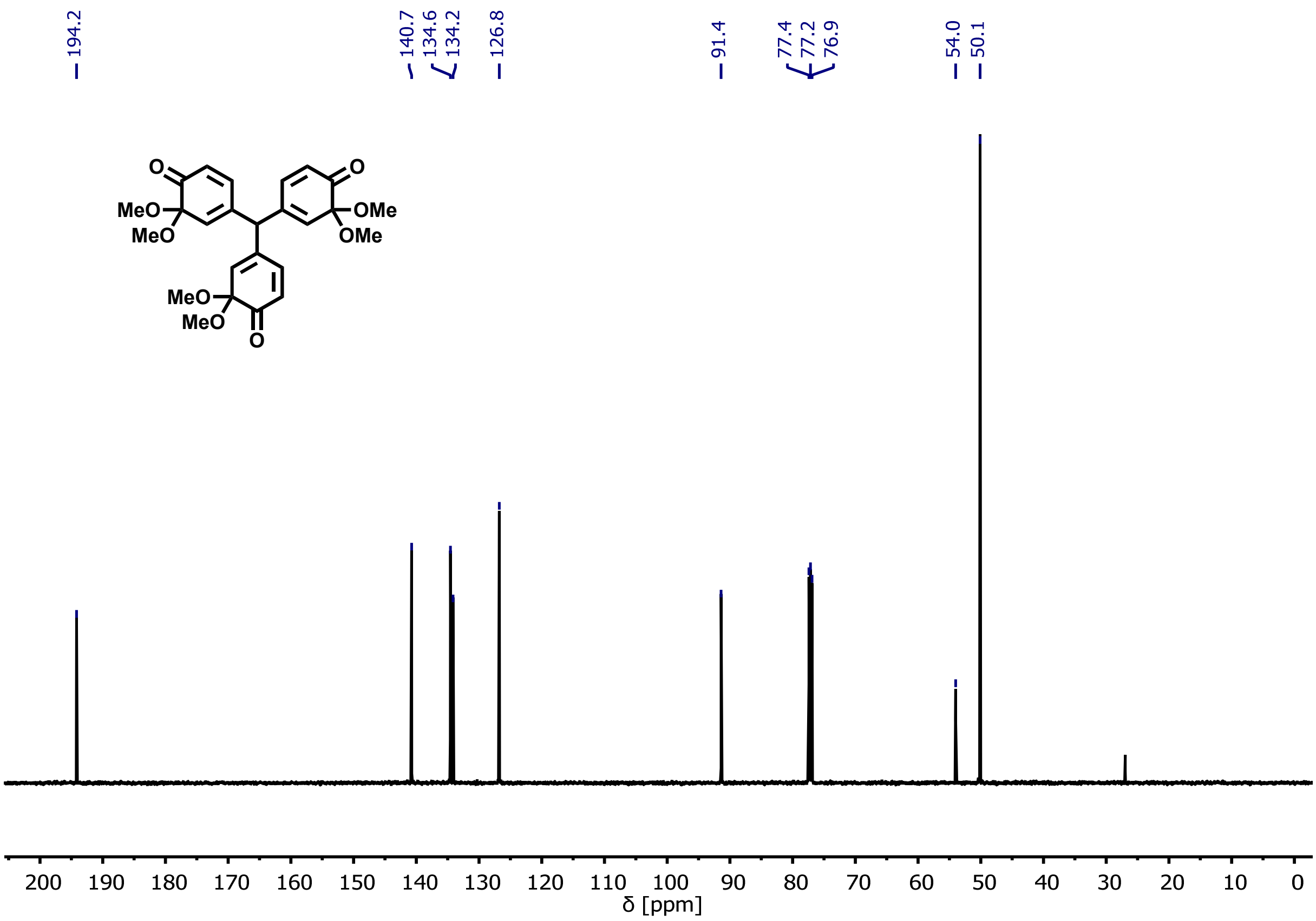

Figure S 3. ^{13}C-NMR spectrum of tMoQ in $CDCl_3$.

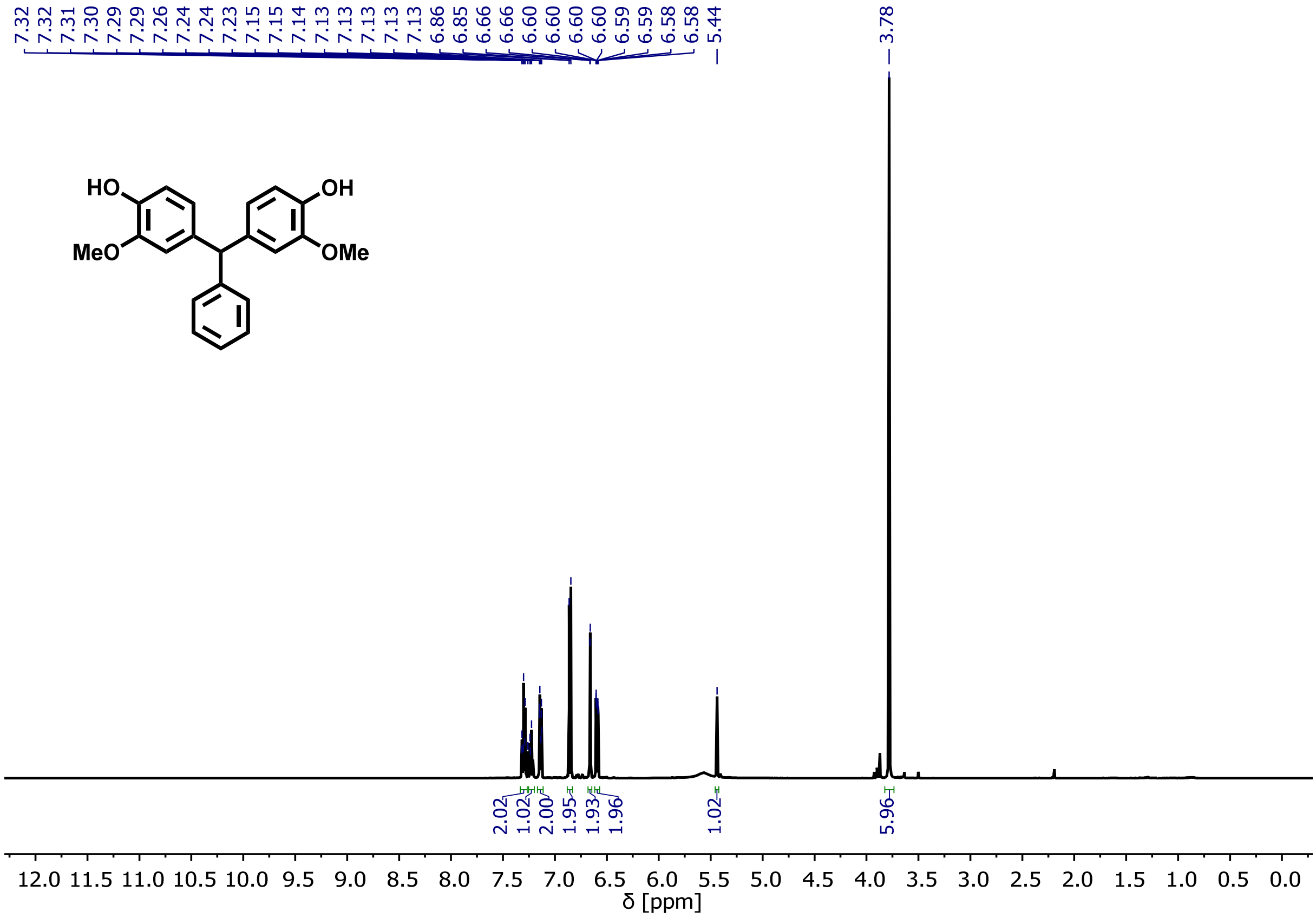

Figure S 4. ^{1}H-NMR spectrum of dG in $CDCl_3$.

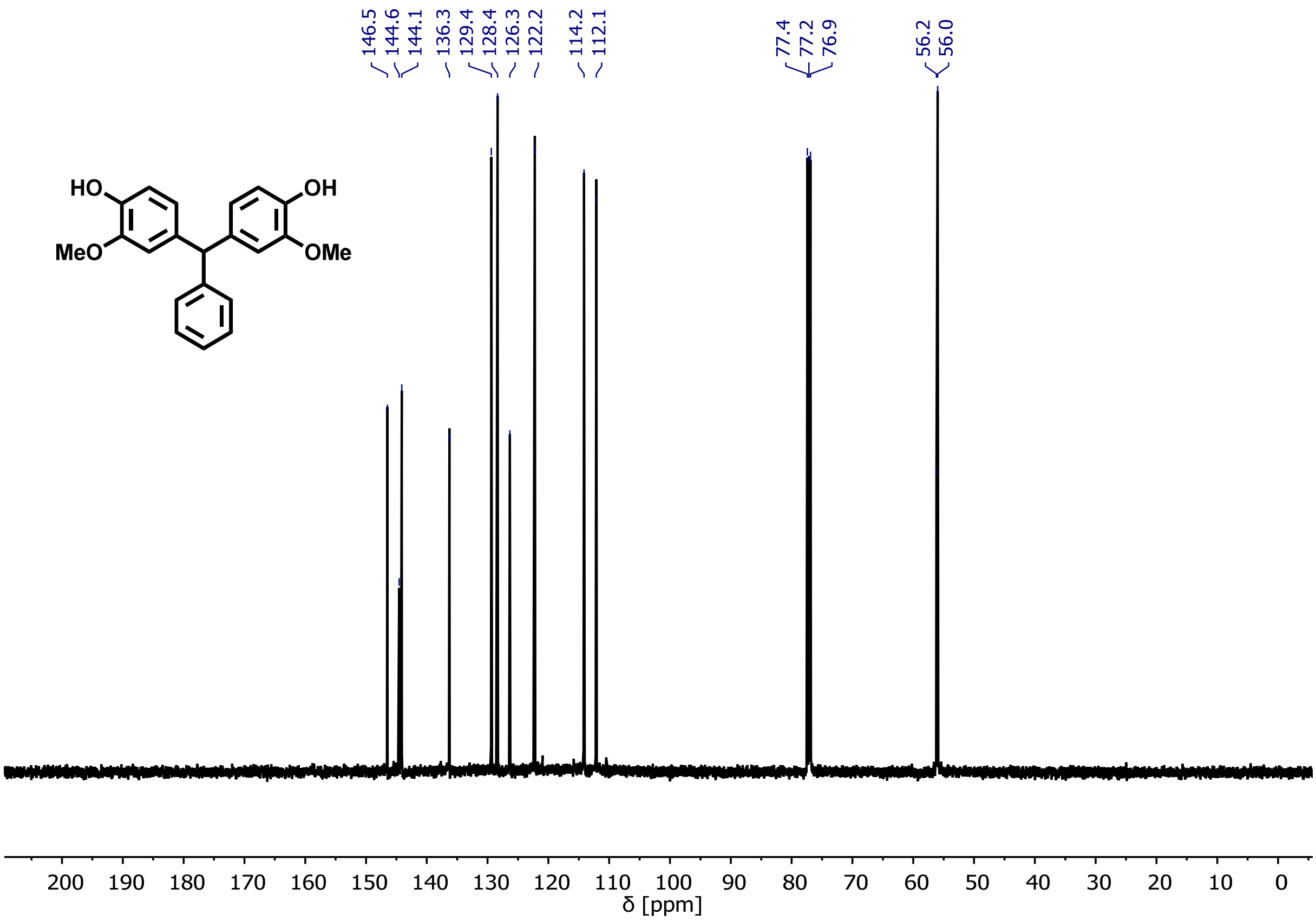

Figure S 5. ^{13}C-NMR spectrum of dG in $CDCl_3$.

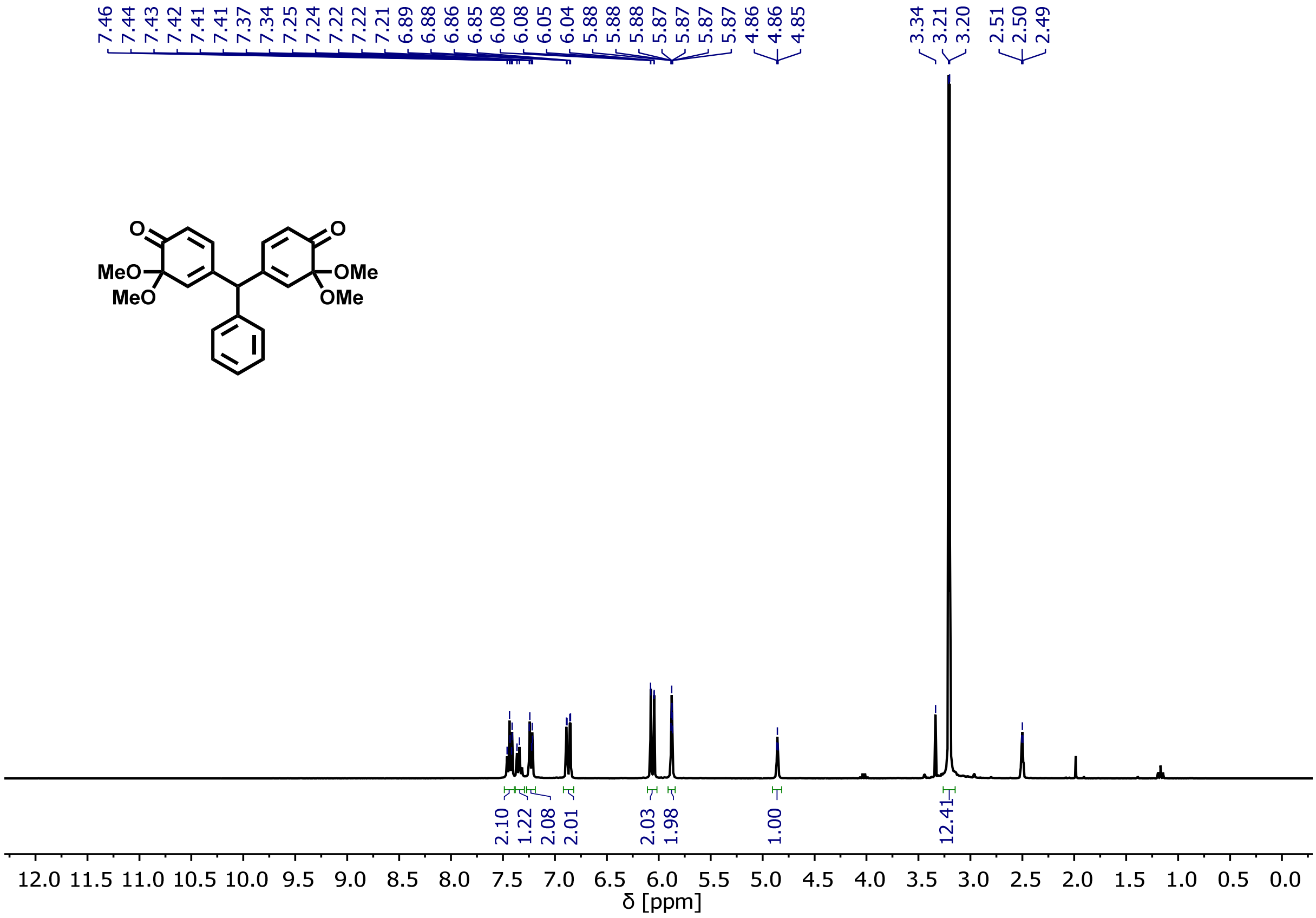

Figure S 6. ^{1}H-NMR spectrum of dMoQ in DMSO-d_6.

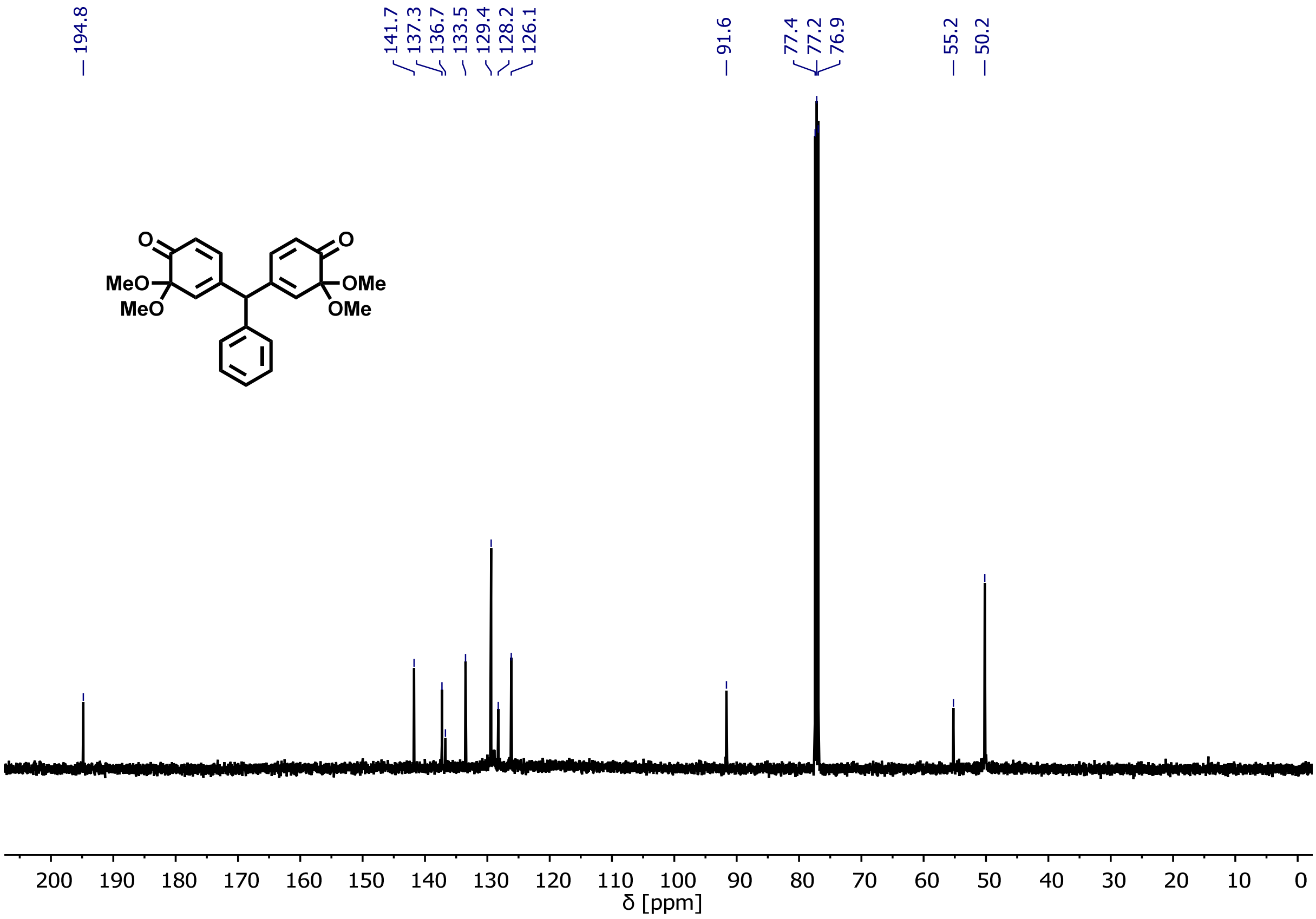

Figure S 7. ^{13}H-NMR spectrum of dMoQ in DMSO-d_6.

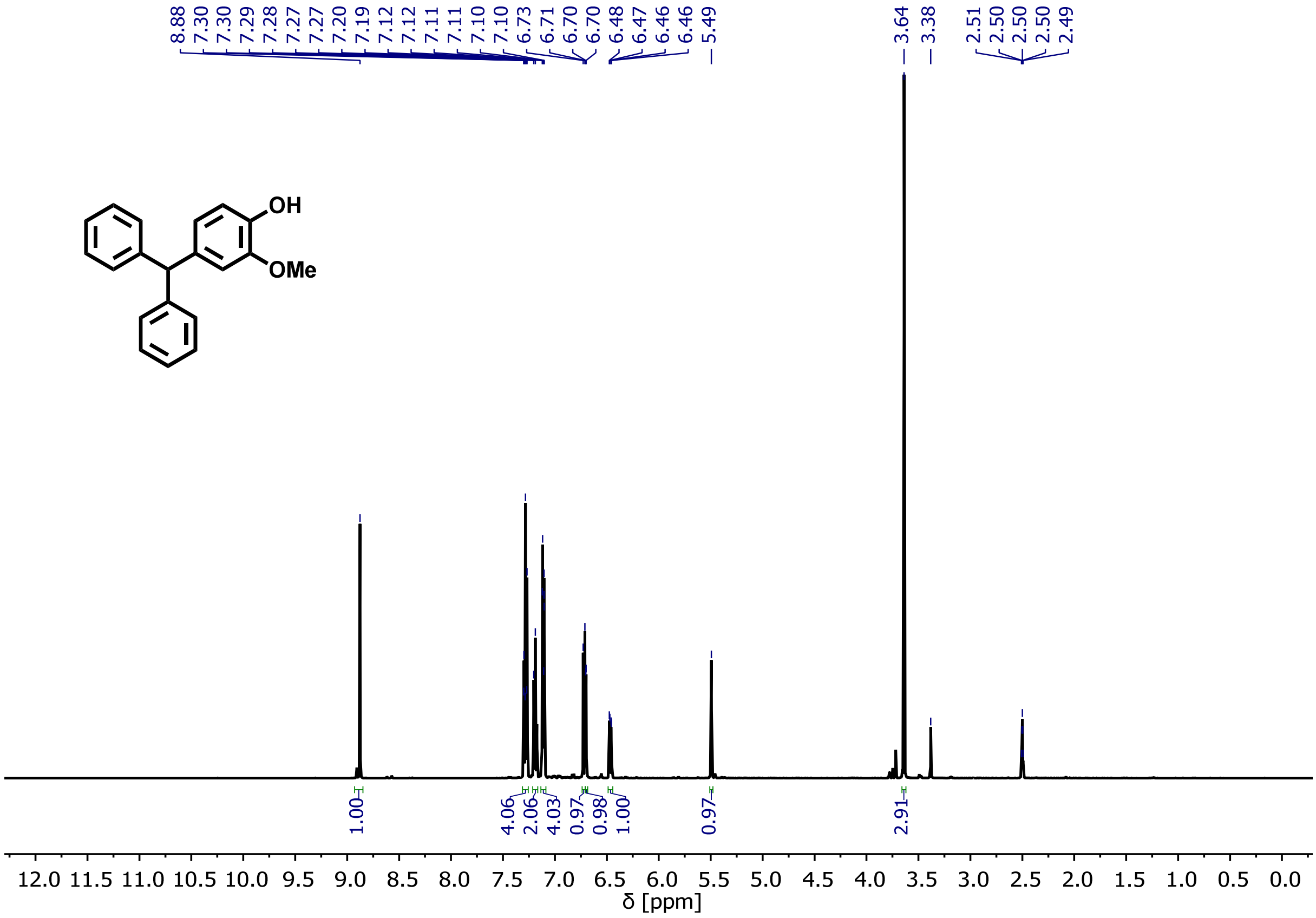

Figure S 8. ^{1}H-NMR spectrum of mG in DMSO-d$_6$.

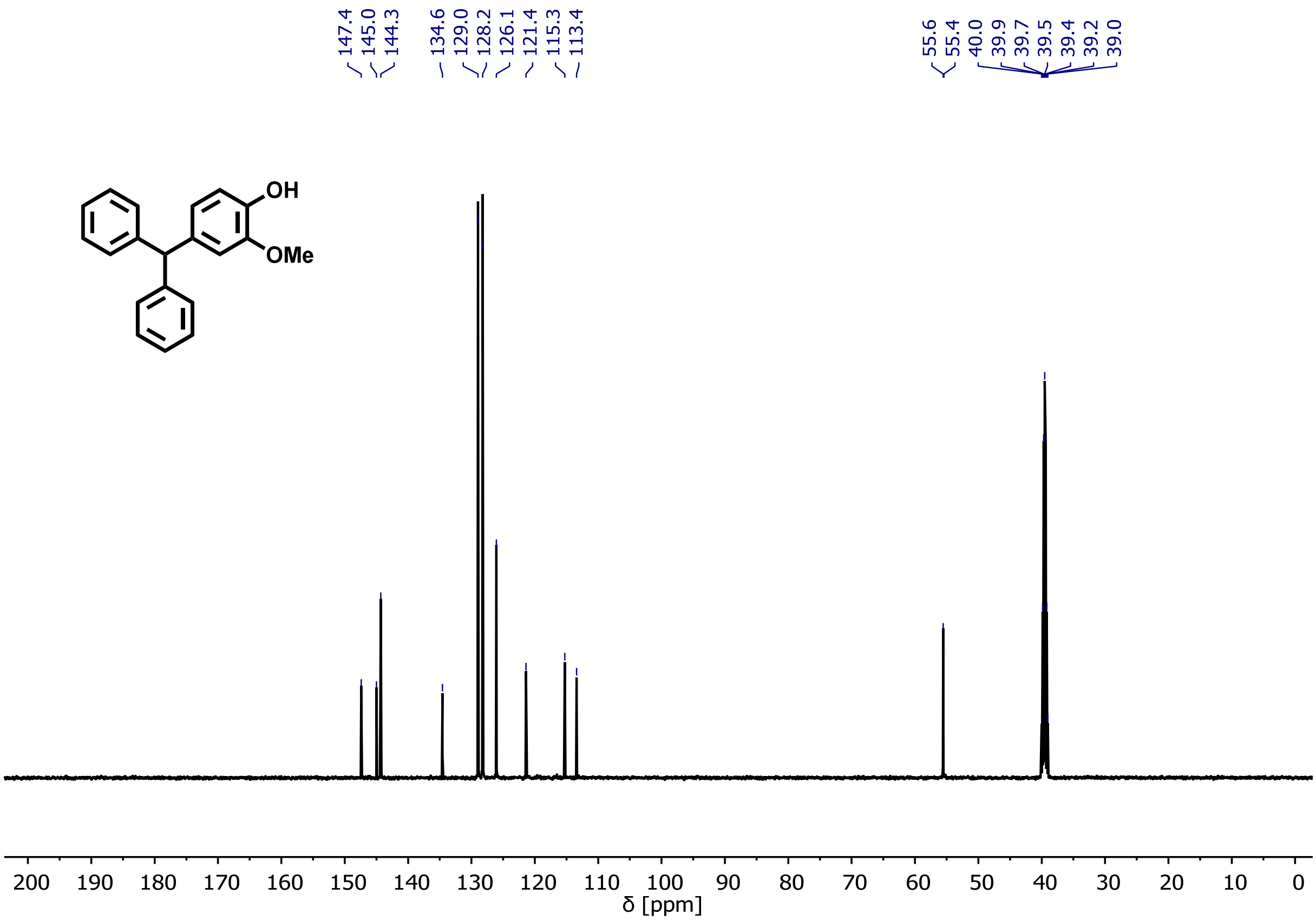

Figure S 9. ^{13}C-NMR spectrum of mG in DMSO-d$_6$.

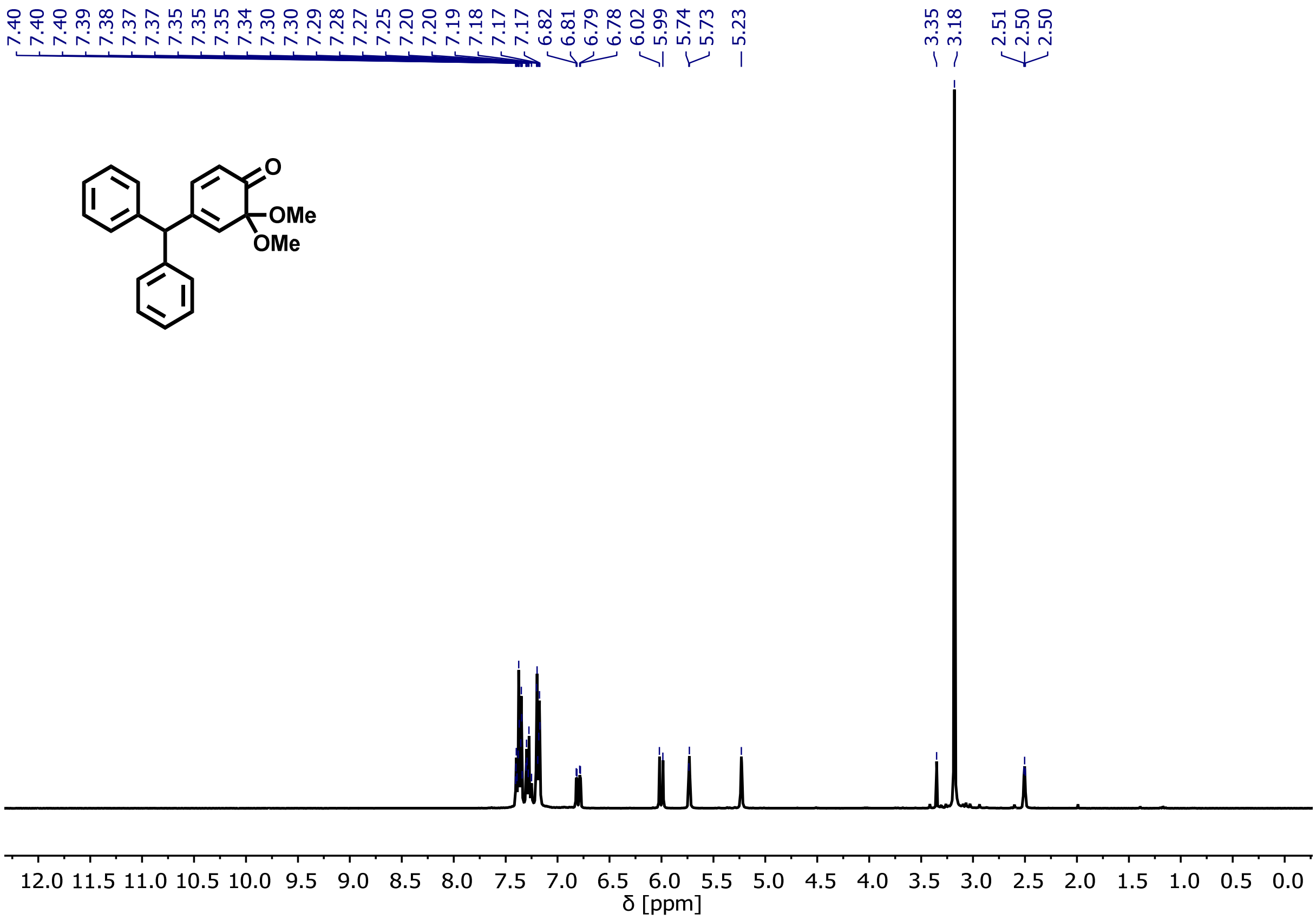

Figure S 10. ^{1}H-NMR spectrum of mMoQ in DMSO-d_6.

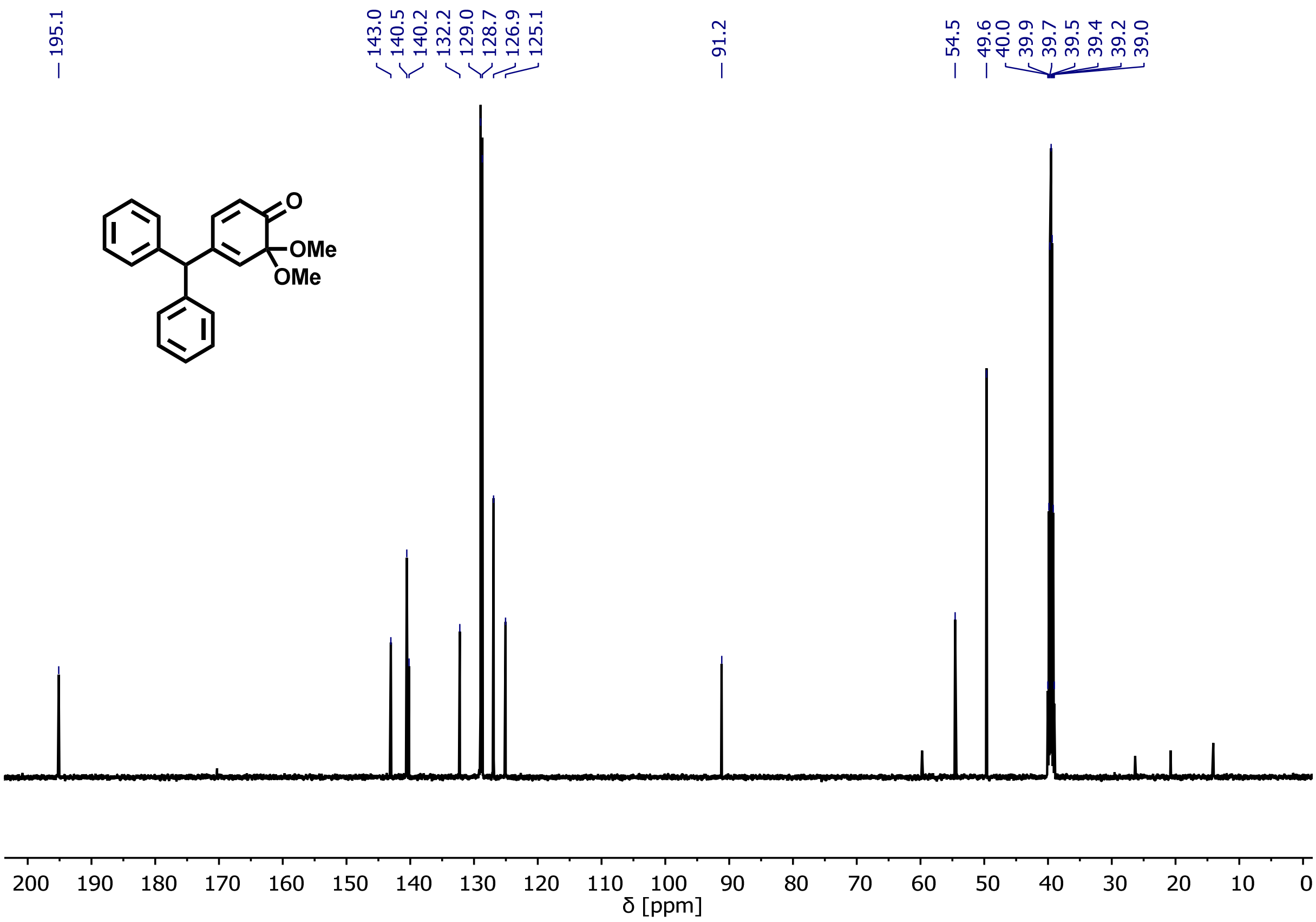

Figure S 11. ^{13}C-NMR spectrum of mMoQ in DMSO-d_6.

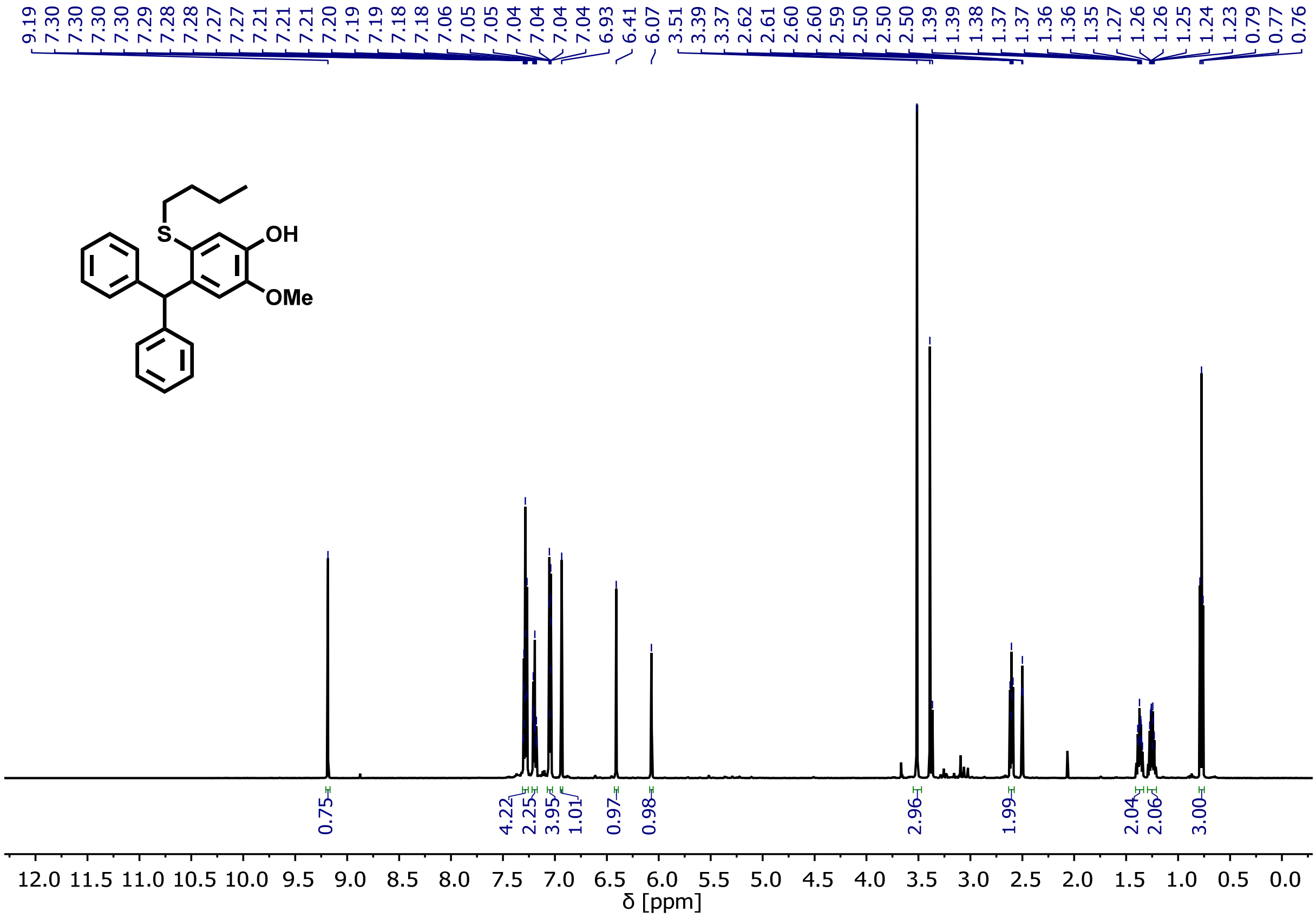

Figure S 12. ^{1}H-NMR spectrum of the mMoQ butanethiol TGC adduct in DMSO-d_6.

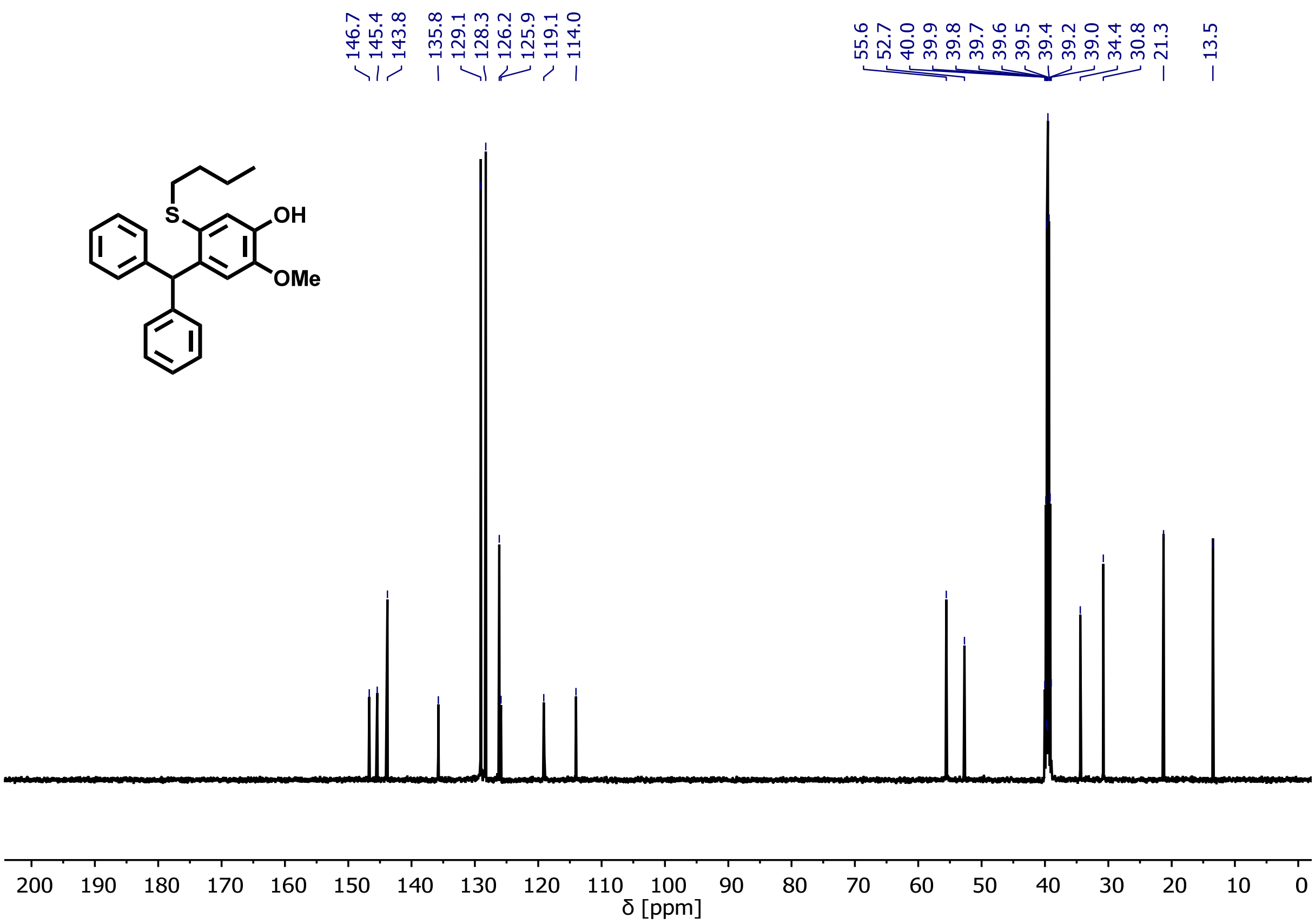

Figure S 13. ^{13}C-NMR spectrum of the mMoQ butanethiol TGC adduct in DMSO-d_6.

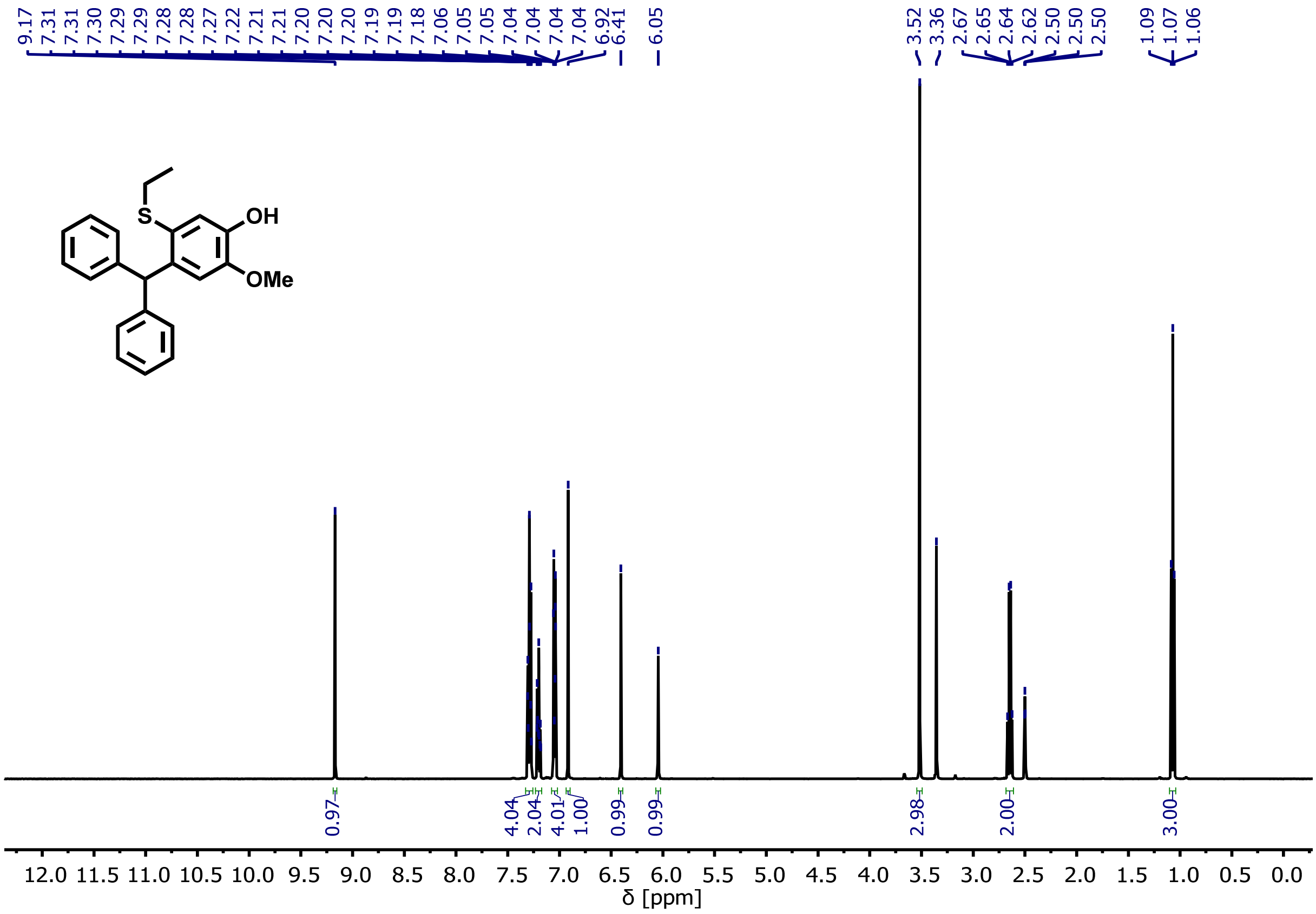

Figure S 14. ^{1}H-NMR spectrum of the mMoQ ethane thiol TGC adduct in DMSO-d_6.

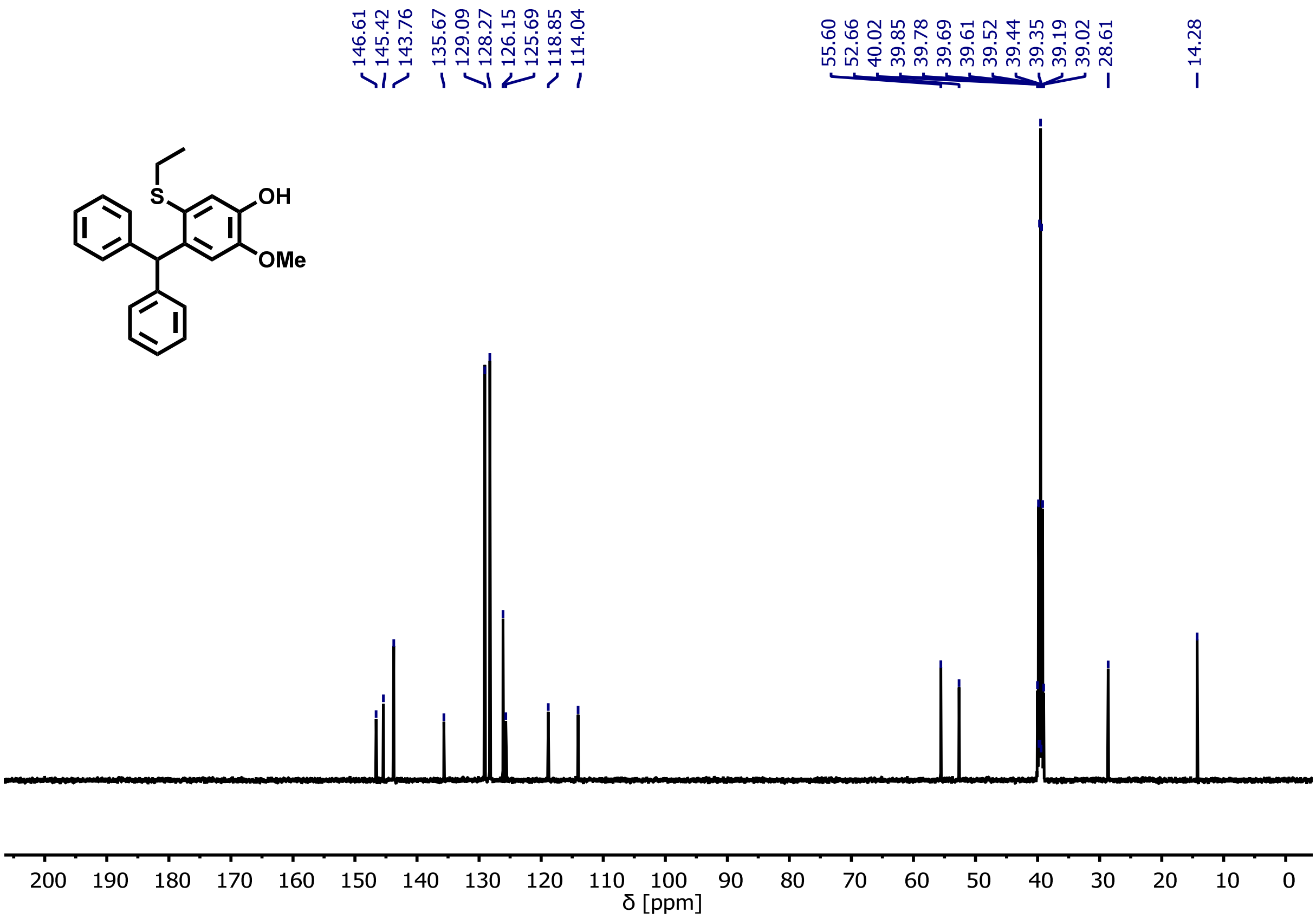

Figure S 15. ^{13}C-NMR spectrum of the mMoQ ethane thiol TGC adduct in DMSO-d_6.

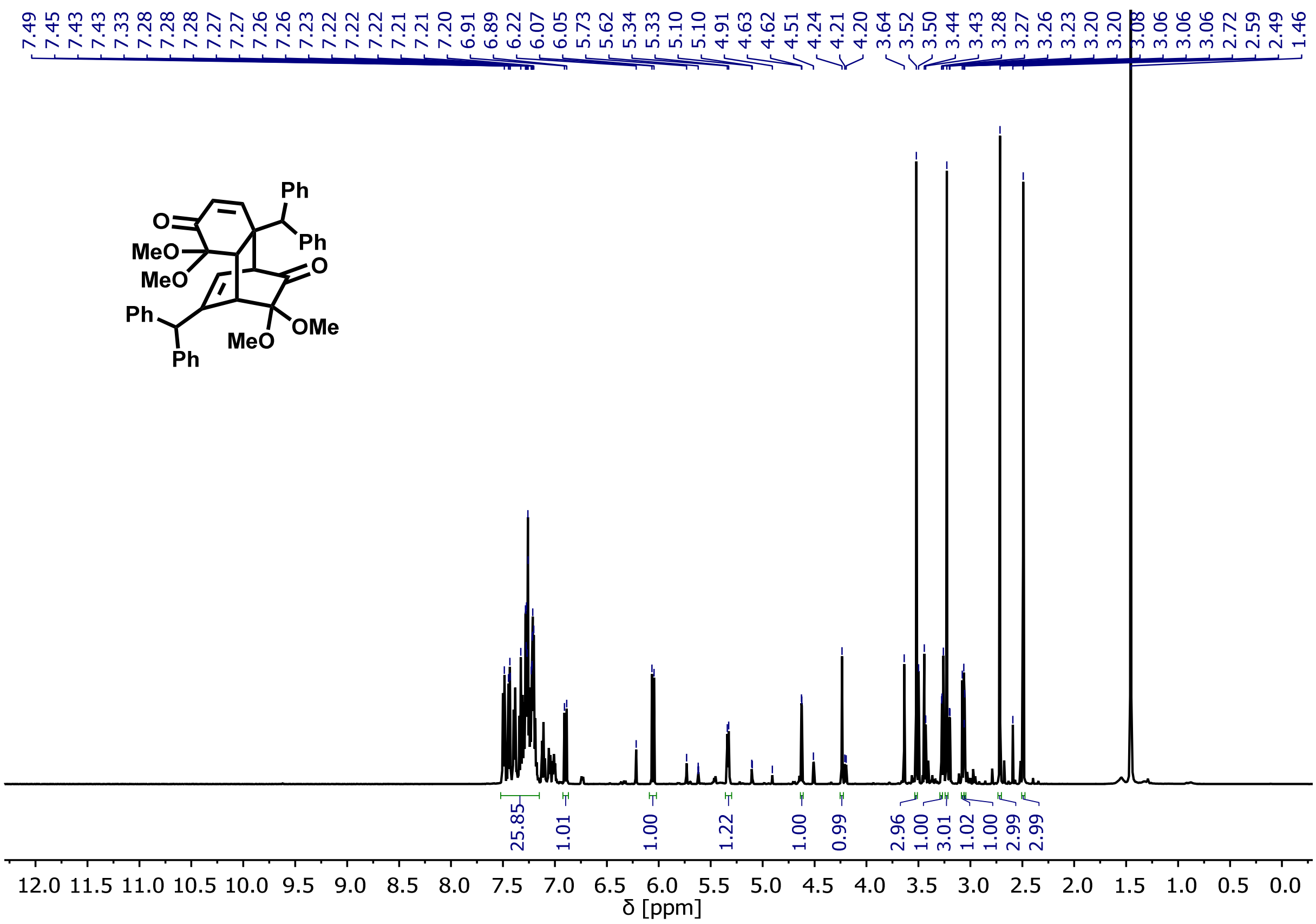

Figure S 16. ^{1}H-NMR spectrum of the mMoQ Diels-Alder dimer in $CDCl_3$.

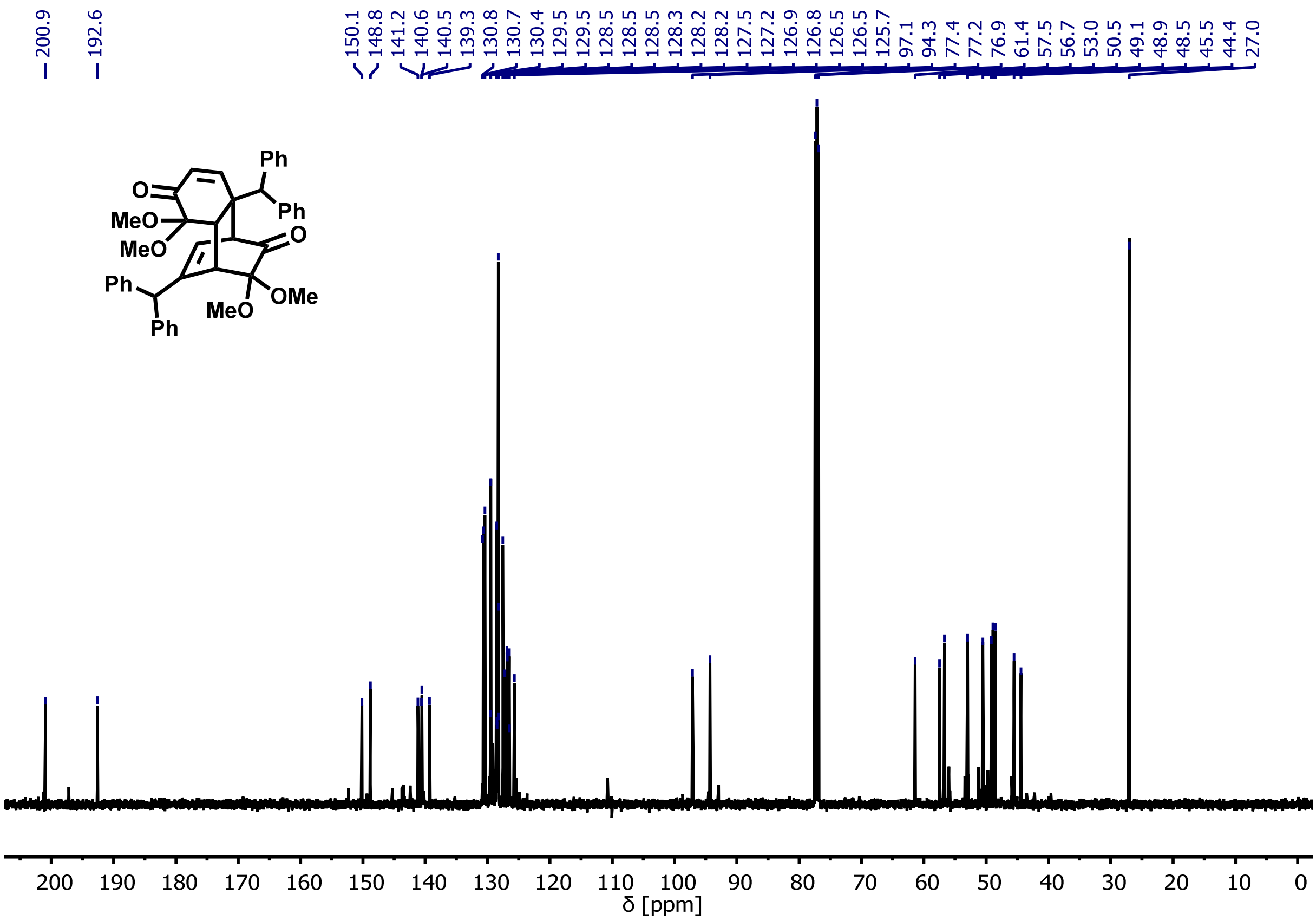

Figure S 17. ^{13}C-NMR spectrum of the mMoQ Diels-Alder dimer in $CDCl_3$.

S5 Experiments

S5.1 Analysis of PIDA mediated oxidation of tG to tMoQ

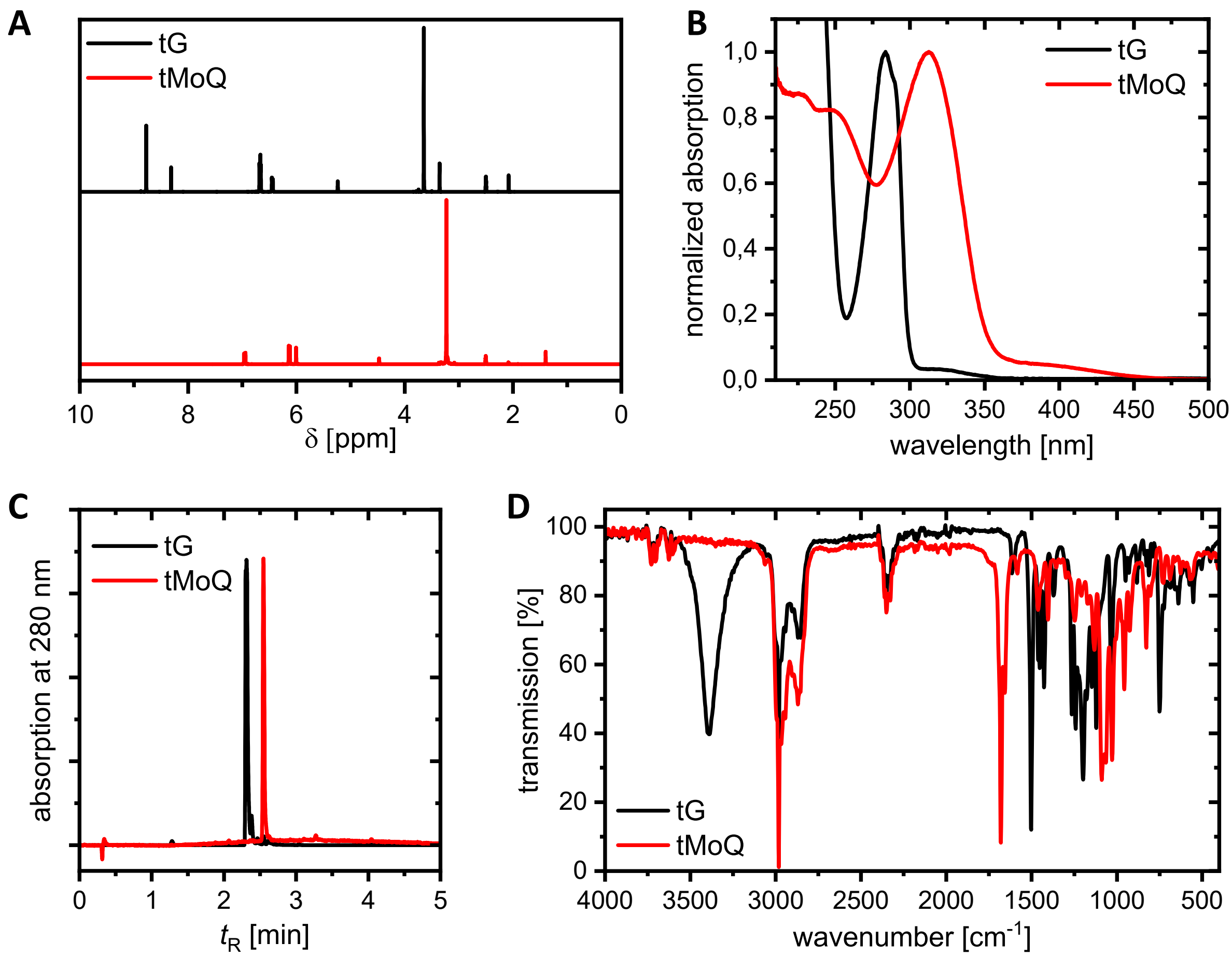

Figure S 18. Analysis of tG before and after oxidation to tMoQ using PIDA via ^{1}H-NMR in DMSO-d_6 (A), UV/vis spectroscopy in methanol (B), UHPLC-ESI-MS (λ = 280 nm, gradient 10-90% B with A: H2O + 0.1% FA and B: ACN + 0.1% FA) (C) and IR spectroscopy (D).

The oxidation of tG to tMoQ was confirmed by ^{1}H-NMR spectroscopy (**Figure S 18 A**) and by UV/vis spectroscopy (**Figure S 18 B**), where a shift of the absorption maximum from 284 nm to 313 nm upon oxidation can be observed. This transformation is also confirmed by UHPLC-ESI-MS analysis, showing a shift in retention time t_R (**Figure S 18 C**) and by IR spectroscopy, where a newly formed carbonyl vibrational band at 1680 cm^{-1} is visible, while the OH vibrational band at 3400 cm^{-1} disappears (**Figure S 18 D**). The purity of synthesized tMoQ was 98%, measured by integration of UHPLC signal.

S5.2 Reactivity of tMoQ in solution

The reactivity of tMoQ with butane thiol (MoQ/T = 1/1) was evaluated in a variety of different solvents at a concentration of 0.1 M (**Table S 3**). The reaction proceeded the fastest in methanol, where a conversion of 73% could be reached after 24 h. Solvent mixtures containing only a small amount of methanol also showed much faster reaction rates, as confirmed by the 2/1 mixture of NMP/MeOH. In general, a drastic decrease in reaction rate could be observed after a reaction time of 10 h. The unreacted MoQ functionalities were verified to still be present using ^{1}H-NMR as well as UHPLC analysis. This is illustrated by the reaction product of tMoQ with $mPEG_3SH$, as this selectively resulted in the twice substituted TGC adduct, making analysis straightforward. The presence of the unreacted MoQ group alongside the TGC groups could thus be confirmed by the presence of MoQ alkene protons in the ^{1}H-NMR spectrum (**Figure S 19**, blue) as well as the corresponding m/z ratio after ionization in the UHPLC-ESI-MS measurement (**Figure S 20**).

Table S 3. Conversion of MoQ groups for the reaction of tMoQ with butane thiol (MoQ/T = 1/1) at a concentration of 0.1 M determined by UHPLC analysis.

**Solvent**	**MoQ conversion after 24 h**
MeOH	73%
NMP/MeOH (2/1)	52%
DMSO	34%
NMP	31%
EtOAc	25%
Toluene	18%
Chloroform	18%
THF	17%
DCM	14%

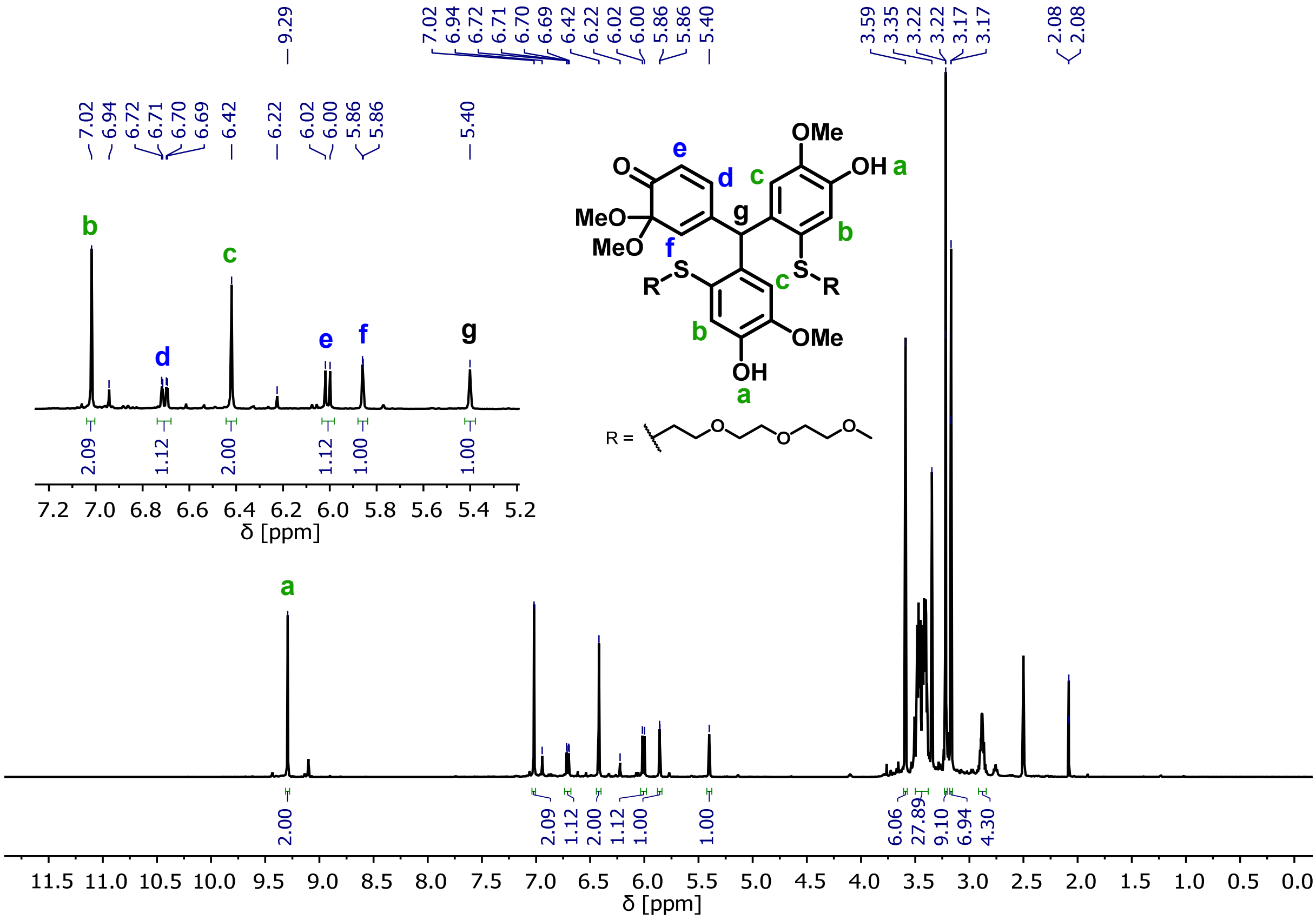

Figure S 19. ^{1}H-NMR spectrum of the reaction product of tMoQ with mPEG$_3$SH (MoQ/T = 1/1) after a reaction time of 6 h in MeOH (0.1 M) obtained by precipitation in MQ water, measured in DMSO-d$_6$.

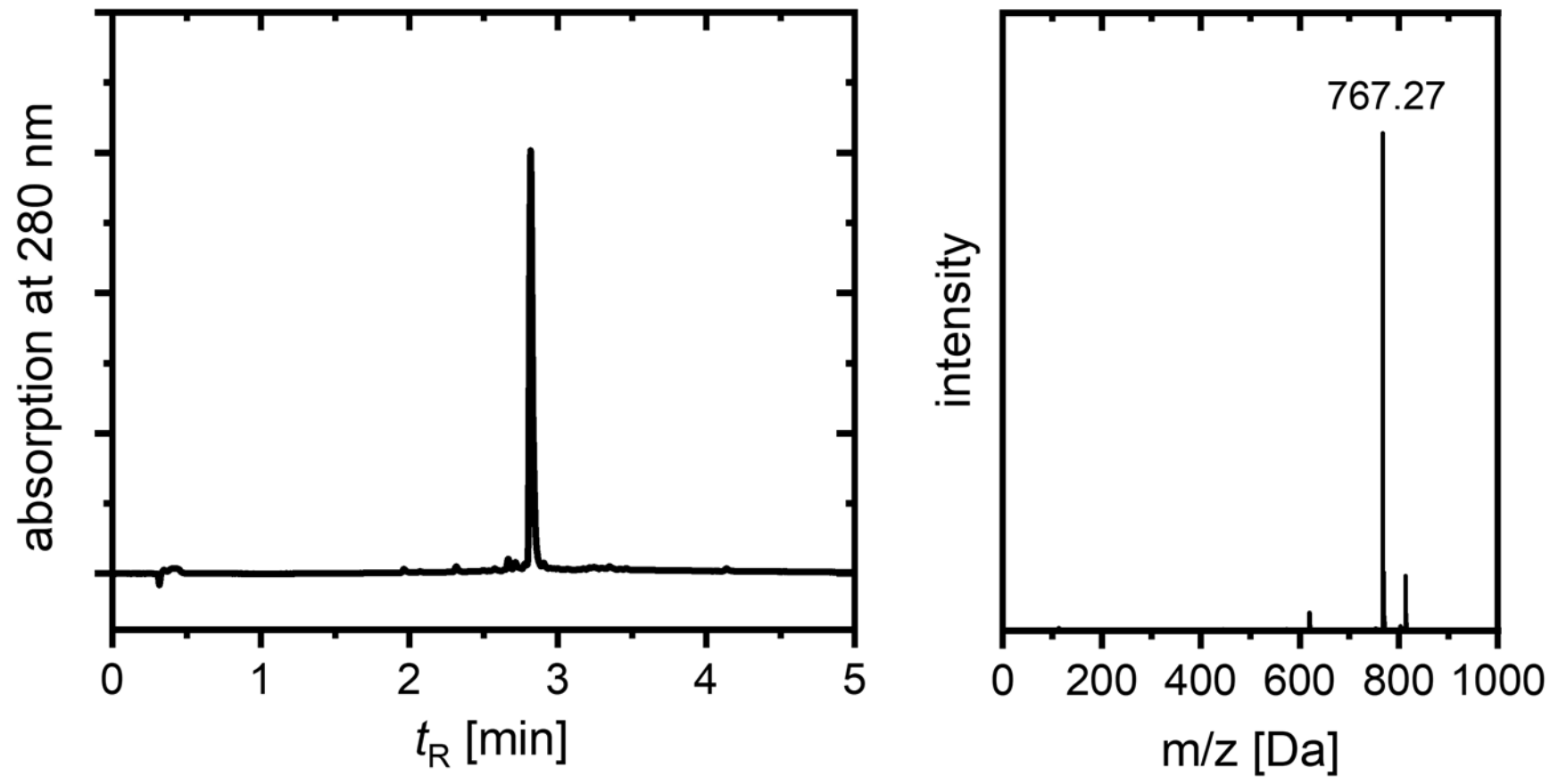

Figure S 20. UHPLC-ESI-MS measurement of the reaction product of tMoQ with mPEG$_3$SH (MoQ/T = 1/1) after 6 h in MeOH (0.1 M) and the corresponding mass spectrum at 2.82 minutes, calculated (m/z): 767.28 [M-H]$^-$.

Since the interesting incomplete MoQ conversion for dMoQ and tMoQ is not caused by decomposition of MoQ groups, several different mechanistic perspectives were explored.

The thiol addition to masked *o*-quinones has been reported to proceed efficiently at the 6-position through a Michael-type addition, followed by rearomatization. This aromatization provides an energetic sink and traps the product. Therefore, the overall MoQ–thiol reaction should not be assumed to be intrinsically reversible once the full addition/rearomatization sequence has occurred. This view is consistent with the mMoQ model compound, which reacted quantitatively with thiols and was not showing evidence for reversibility. This seems to exclude the establishment of an equilibrium as a possible explanation.

The computed activation barriers and reaction free energies for all investigated MoQ variants and reaction steps are summarized in **Table S 4** and **Table S 5** at the DFT and DLPNO-CCSD(T) levels of theory, respectively. The activation barriers for both steps are consistently high across all systems and show only minor variation between mMoQ, dMoQ and tMoQ, while the reaction free energies are similarly comparable across the series. As a consequence, these energetic differences are insufficient to rationalize the experimentally observed variation in product yields between the different MoQ structures. We note that in the present calculations a single explicit methanol molecule was employed to facilitate the proton transfer steps. Given the protic and highly solvating nature of methanol, the use of a single solvent molecule may not adequately capture cooperative hydrogen-bonding effects. Inclusion of an explicit solvent cluster is therefore expected to be necessary to obtain activation barriers that are more representative of the true solution-phase reactivity.

Table S 4. DFT-computed activation barriers E_A [kcal·mol^{-1}] and reaction free energies ΔG [kcal·mol^{-1}] for all investigated MoQ variants. Entries marked x indicate cases where no transition state could be located.

	Subst.	Step	Reac. [E_h]	TS [E_h]	Prod. [E_h]	E_A [kcal·mol^{-1}]	ΔG [kcal·mol^{-1}]
**mMoQ**	1st	1	–1940.9649	–1940.9330	–1940.9613	20.1	2.3
		2	–1940.9646	–1940.9155	–1941.0118	30.8	–29.6
**dMoQ**	1st	1	–2245.1364	–2245.1039	–2245.1271	20.4	5.8
		2	–2245.1358	–2245.0879	–2245.1833	30.1	–29.8
	2nd	1	–2685.9640	–2685.9315	–2685.9581	20.4	3.7
		2	–2685.9629	–2685.9138	–2686.0126	30.8	–31.2
**tMoQ**	1st	1	–2549.3072	–2549.2767	–2549.3095	19.1	–1.5
		2	–2549.3096	–2549.2591	–2549.3546	31.7	–28.2
	2nd	1	–2990.1404	–2990.1054	–2990.1382	21.9	1.4
		2	–2990.1367	–2990.0881	–2990.1818	30.5	–28.3
	3rd	1	x	x	x	x	x
		2	–3430.9632	–3430.9033	–3431.0049	37.6	–26.2

Table S 5. DLPNO-CCSD(T)-computed activation barriers E_A [kcal·mol^{-1}] and reaction free energies ΔG [kcal·mol^{-1}] for all investigated MoQ variants. Entries marked x indicate cases where no transition state could be located.

	Subst.	Step	Reac. [E_h]	TS [E_h]	Prod. [E_h]	E_A [kcal·mol^{-1}]	ΔG [kcal·mol^{-1}]
**mMoQ**	1st	1	–1938.9477	–1938.9151	–1938.9527	20.5	–3.1
		2	–1938.9581	–1938.9013	–1938.9960	35.6	–23.8
**dMoQ**	1st	1	–2242.8535	–2242.8197	–2242.8523	21.2	0.8
		2	–2242.8606	–2242.8057	–2242.8986	34.5	–23.9
	2nd	1	–2683.2174	–2683.1841	–2683.2202	20.8	–1.8
		2	–2683.2263	–2683.1698	–2683.2669	35.5	–25.5
**tMoQ**	1st	1	–2546.7542	–2546.7236	–2546.7651	19.2	–6.8
		2	–2546.7658	–2546.7093	–2546.8026	35.4	–23.1
	2nd	1	–2987.1250	–2987.0901	–2987.1315	21.9	–4.0
		2	–2987.1314	–2987.0764	–2987.1687	34.5	–23.4
	3rd	1	x	x	x	x	x
		2	–3427.4982	–3427.4313	–3427.5324	42.0	–21.4

Taking into account that the thiol addition proceeded at 6-position of the three ring cores constituting the trityl structural element, substantial steric pressure might be expected for the thiol additions. Intriguingly, such steric regulation provides a quite satisfactory first-order explanation of the observed reaction degrees. When considering the central tritylic C-H bond as the "upper" face of the tMoQ molecule, the reactive C_6-H positions of the MoQ groups can point either upward or downward. This results in four orientation classes of three up, two up, one up, or none up, with a statistical distribution of 1:3:3:1, assuming similarly populated isoenergetic conformers. Due to the tetrahedral nature of the central carbon atom, the reactive C_6-H group of the MoQ unit is pointed either slightly inward when downward oriented or pointed slightly outward when upward oriented. This results in full substitution of MoQ groups facing upward, but limits the number of substitutions to one on the lower face due to steric demands of the newly introduced substituent. Thus, conformers with two downward-facing C_6-H positions are expected to reach a maximum substitution degree of two, whereas conformers with three downward-facing C_6-H positions should reach only one. Weighting these limits by the statistical distribution gives an average substitution degree of:

$(1\times3+3\times3+3\times2+1\times1)/8 = 2.375$.

Applying the same model to dMoQ gives an average substitution degree of 1.75, as only the conformer with both C_6-H positions pointing down is limited to a single substitution. Despite the simplicity, this statistical-kinetic model was in surprisingly good agreement with the experimentally observed substitution degrees of 1.7 for dMoQ and 2.3 for the tMoQ. The model therefore suggested that incomplete conversion is not necessarily caused by insufficient intrinsic reactivity of the MoQ units, but may result from conformational orientation and steric self-blocking after the first substitution events.

It should be noted that this minimal model relies on several assumptions. In particular, the relevant conformers of the multi-MoQ systems have to be close enough in energy to be comparably populated

and substitution on the lower face create sufficient steric hindrance to suppress further reactions at neighboring sites at the same face. However, computational calculations of rotational barriers of substituted MoQ units, e.g. tMoQ indicated the ability of remaining MoQ groups to freely rotate at room temperature. Thus, conformational statistics and steric shielding alone may not fully explain the observed incomplete conversion.

To assess whether conformational dynamics could account for the observed incomplete thiol conversion, the rotational barrier of the unthiolated MoQ ring in a tMoQ species bearing two already-attached butanethiol groups was computed. Three conformations were considered, differing in the orientation of the C–H units at the positions bearing the thiol substituents: both pointing downward (0 up), one pointing upward (1 up), and both pointing upward (2 up). For each conformation, the dihedral angle of the remaining unsubstituted MoQ ring was systematically varied and the relative energies with respect to the lowest energy conformer are reported in **Table S 6**. In all three cases, the computed rotational barriers were small, confirming that free rotation of the remaining MoQ group is feasible at room temperature. This result indicates that conformational statistics and steric shielding alone are insufficient to fully account for the incomplete conversion.

Table S 6. Relative energies for the rotation of the unsubstituted MoQ ring in a tMoQ species bearing two butanethiol substituents. Three conformations of the C-H units at the thiolated positions are considered: both pointing downward (0 up), one pointing upward (1 up), and both pointing upward (2 up). For each conformation, the dihedral angle of the remaining MoQ ring was scanned; absolute energies E (in E_h) and relative energies ΔE (in kcal·mol^{-1}) with respect to the lowest-energy conformer are listed.

angle [°]	0 up E [E_h]	ΔE [kcal·mol^{-1}]	angle [°]	1 up E [E_h]	ΔE [kcal·mol^{-1}]	angle [°]	2 up E [E_h]	ΔE [kcal·mol^{-1}]
135	–2527.8979	7.00	141	–2527.9054	3.10	21	–2527.9109	1.10
146	–2527.8975	7.30	152	–2527.9055	3.00	32	–2527.9117	0.60
156	–2527.8958	8.30	162	–2527.9045	3.60	42	–2527.9127	0.00
167	–2527.8959	8.30	173	–2527.9025	4.90	53	–2527.9123	0.20
177	–2527.8947	9.10	183	–2527.9011	5.80	63	–2527.9108	1.20
188	–2527.9016	4.70	194	–2527.8997	6.60	74	–2527.9083	2.70
199	–2527.9015	4.80	205	–2527.9064	2.40	85	–2527.9055	4.50
209	–2527.9019	4.50	215	–2527.9060	2.70	95	–2527.9017	6.80
220	–2527.9014	4.80	226	–2527.9053	3.10	106	–2527.9029	6.10
230	–2527.9001	5.70	236	–2527.9046	3.60	116	–2527.9034	5.80
241	–2527.8982	6.80	247	–2527.9038	4.10	127	–2527.9041	5.40
251	–2527.8963	8.00	257	–2527.9024	4.90	137	–2527.9056	4.40
262	–2527.8997	5.90	268	–2527.9005	6.10	148	–2527.9073	3.30
273	–2527.9033	3.60	279	–2527.8999	6.50	159	–2527.9087	2.50
283	–2527.9062	1.80	289	–2527.8985	7.40	169	–2527.9089	2.40
294	–2527.9082	0.60	300	–2527.9102	0.00	180	–2527.9074	3.30
304	–2527.9091	0.00	310	–2527.9103	0.00	190	–2527.9040	5.40
315	–2527.9089	7.00	321	–2527.9099	3.10	201	–2527.8988	1.10

This observation led us to extend the mechanistic discussion toward the intrinsic reactivity of the Michael acceptor and the possible reversibility of the initial thiol–MoQ Michael-type addition step. This assumption seems to be chemically reasonable, as related quinonoid and Michael-type systems are known in which thiol addition is equilibrium-controlled and not fully quantitative. Thus, incomplete substitution in the present MoQ systems could plausibly arise from the combined effects of conformational statistics, steric shielding, and non-quantitative thia-Michael-type addition. Whereas completion of the full addition/rearomatization sequence prevents reversibility, the first step of the thiol-MoQ reaction corresponds to thiol addition to an enone-type Michael acceptor, i.e. a conjugated ketone. This initial step might be reversible before rearomatization traps the product. Such behavior is well reported for classical thiol-Michael additions. For example, M. F. Greaney *et al.* used reversible thiol-Michael addition of enones to establish a dynamic combinatorial library.[17] S. J. Rowan *et al.* described dynamic thia-Michael benzalcyanoacetate networks, including equilibrium constants that rationalize non-quantitative conversions as low as 24%.[18]

Taking this into account, a significant downregulation of the MoQ activity in dMoQ and tMoQ might occur during the progress of thiol addition. Computational assessments helped to identify changes in the stability of a cationic intermediate that can be considered a reactive species initiating the thiol addition step (**Scheme S 1**). This mechanistic assumption was experimentally supported by the observation that all methoxy groups of the acetal structures are quantitatively exchangeable by methanol-d_4, as confirmed by ^{1}H NMR (**Figure S 21**) and ESI-MS (**Figure S 22**). Interestingly the central C-H proton of the trityl unit was not exchanging under the given conditions, excluding reaction pathways involving anionic trityl-species to interfere with the thiol-Michael-reaction.

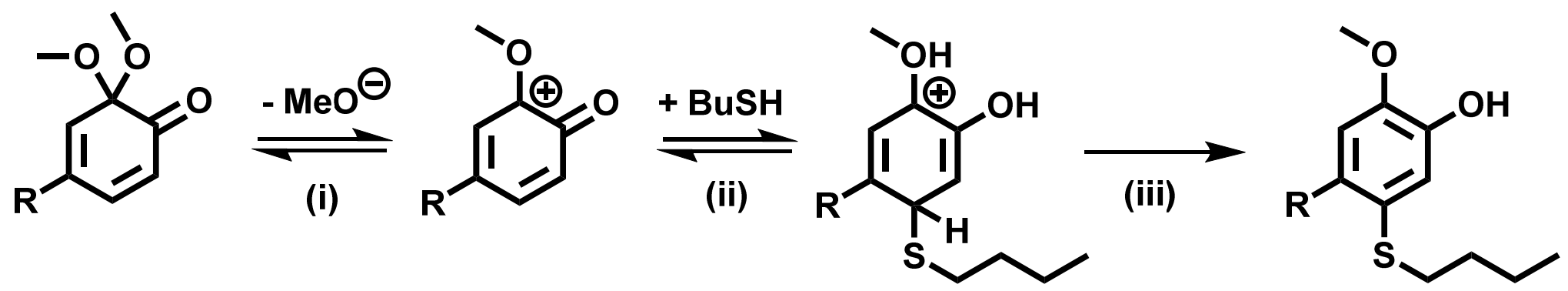

Scheme S 1. Postulated mechanism for thiol addition to MoQ groups via ketal methoxide exchange to form a carbocation intermediate.

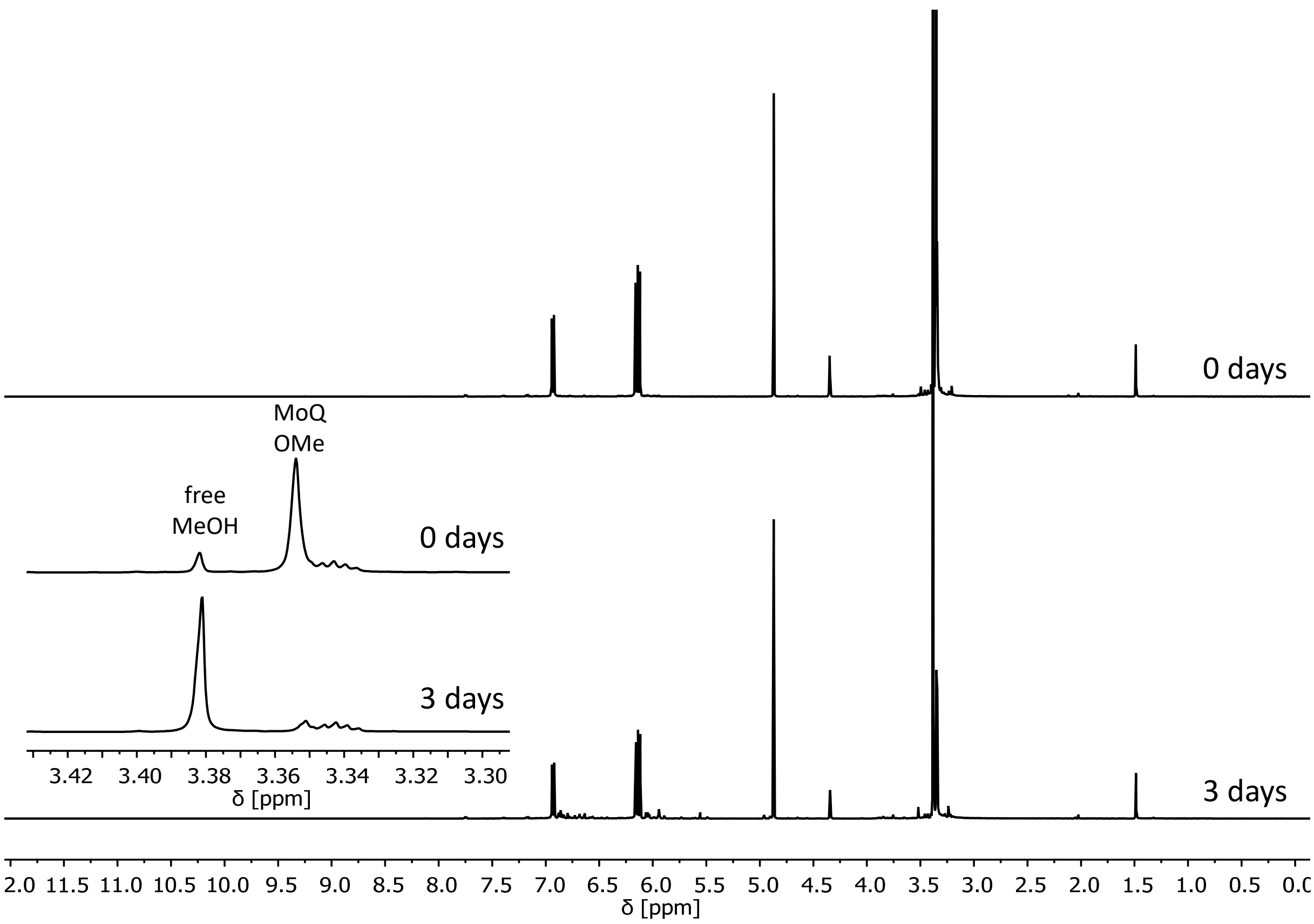

Figure S 21. ^{1}H-NMR of tMoQ in MeOH-d_4 measured directly (top) and after 3 days (bottom). Enlargement of the methoxy region (3.3-3.4 ppm) reveals the changes associated with methoxy exchange.

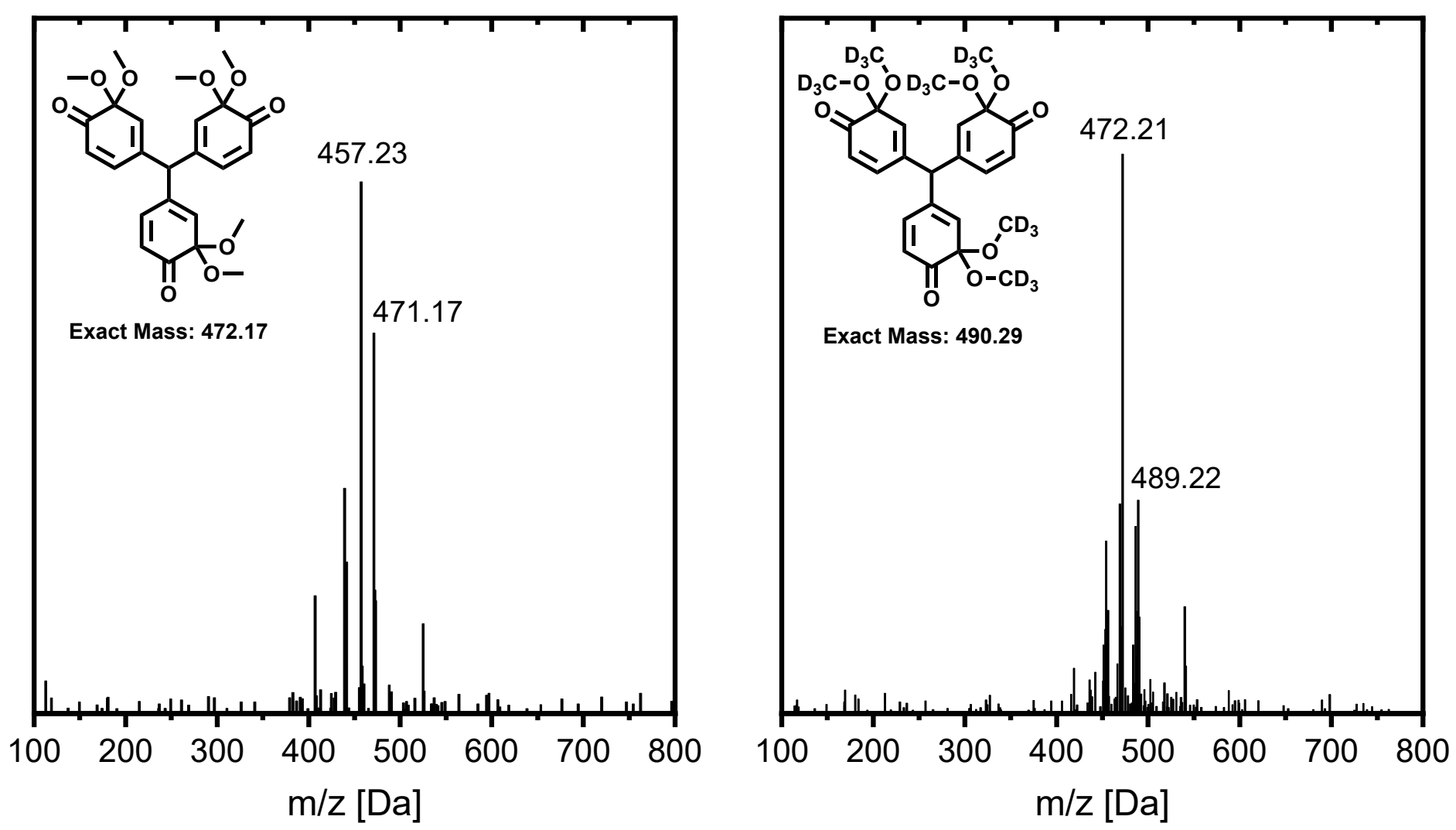

Figure S 22. ESI-MS spectrum with negative ionization of tMoQ before (left) and after 3 days in MeOH-d_4 (after). Full exchange becomes evident from the increase of $[M-H]^-$ mass from m/z=471.17 to m/z=489.22, corresponding to an increase of 18 Da from substitution of all six CH_3O-groups with CD_3O-groups. This is also reflected in fragmentation, as the major fragment ion changes from $[M-CH_3]^-$ with m/z=457.23 to $[M-CD_3]^-$ with m/z=472.21.

Gibbs free energies of all relevant stationary points were computed (**Table S 7**). The pathway consists of three steps: (i) loss of a methoxy group, (ii) nucleophilic attack of butanethiol, and (iii) deprotonation accompanying rearomatization and TGC formation. For Steps 1 and 3, the number of atoms changes between reactant and product due to loss of a methoxy anion and a proton, respectively. An isolated

computation of these small fragments is complicated by the lack of an appropriate reference state in the implicit solvent model, as the solvation of a bare anion or proton is not reliably described by CPCM. Therefore, rather than computing absolute *ΔG* values, the energy trend (*ΔΔG*) was assessed by comparing only the larger molecular fragments, allowing evaluation of the relative thermodynamic stability across the MoQ series. For Step 2, the atom count is conserved, permitting a direct *ΔG* evaluation. The Gibbs free energy of the reactant and the separately computed Gibbs free energy of butanethiol (G = –556.4735 E_h) were summed and taken as the reference, from which the difference to the respective product was calculated. It should be noted that a thermochemical correction of approximately 10-12 kcal mol^{-1} needs to be taken into account due to the reactant and butanethiol being computed in separate calculations, meaning the actual free energy differences are expected to be higher by this amount.

Table S 7. Computed Gibbs free energies [E_h] of reactants and products and corresponding energy trends *ΔΔG* [kcal·mol^{-1}] for steps 1 and 3, and reaction free energies *ΔG* [kcal·mol^{-1}] for step 2 of the methoxy cleavage pathway for all investigated MoQ variants.

		**reactant [E_h]**	**product [E_h]**	***ΔΔG* [kcal·mol^{-1}]**
**$^{-}$OMe cleavage**	mMoQ	–1037.4553	–922.1809	5.3
	dMoQ#1	–1341.6243	–1226.3515	4.3
	dMoQ#2	–1782.4489	–1667.1815	0.9
	tMoQ#1	–1645.7926	–1530.5162	6.6
	tMoQ#2	–2086.6150	–1971.3448	2.7
	tMoQ#3	–2527.4410	–2412.1752	0.0
		**reactant [E_h]**	**product [E_h]**	***ΔG* [kcal·mol^{-1}]**
**R-SH attack**	mMoQ	–922.1809	–1478.6652	–6.7
	dMoQ#1	–1226.3515	–1782.8365	–7.2
	dMoQ#2	–1667.1815	–2223.6627	–4.8
	tMoQ#1	–1530.5162	–2087.0025	–8.0
	tMoQ#2	–1971.3448	–2527.8318	–8.5
	tMoQ#3	–2412.1752	–2968.6493	–0.4
		**reactant [E_h]**	**product [E_h]**	***ΔΔG* [kcal·mol^{-1}]**
**TGC formation**	mMoQ	–1478.6652	–1478.2768	2.0
	dMoQ#1	–1782.8365	–1782.4489	1.5
	dMoQ#2	–2223.6627	–2223.2730	2.8
	tMoQ#1	–2087.0025	–2086.6150	1.4
	tMoQ#2	–2527.8318	–2527.4410	3.5
	tMoQ#3	–2968.6493	–2968.2640	0.0

The data are consistent with a mechanism in which loss of MeO^- generates a cationically activated $MoQ^+(-MeO^-)$-species that is a highly activated acceptor for reaction with thiols. Computational calculations along the tMoQ addition cascade (tMoQ → TGC/MoQ_2 → TGC_2/MoQ → TGC_3) indicate that MeO^- release to form the reactive cation intermediate becomes increasingly facile with increasing

thiol substitution. This trend reverses for the following thiol addition, where the first and second thiol additions are similarly favored, whereas a substantial decrease in reactivity is predicted for the third addition. Consistent behavior was observed for dMoQ, but to a lesser extent, where the first thiol addition is facile and the second addition to the TGC/MoQ intermediate is considerably less favored. Based on this computational evidence, one may speculate that the incomplete conversion of dMoQ and tMoQ was associated with a loss of activation strongly slowing down the final thiol-addition step. This would also be consistent with a change in reaction rate after the first thiol addition to dMoQ and after the second thiol addition to tMoQ.

Overall, thiol-Michael adduct formation in the present multi-MoQ monomers appeared to be more dynamic and more sensitive to the molecular environment than initially assumed. Substituent effects, potentially sterics and conformational dynamics and finally trapping in the aromatic state determine whether conversion is complete. Therefore, incomplete thiol addition in the present masked-o-quinone congeners is chemically plausible, and the model provides a chemically reasonable explanation for the observed conversion, although it should be regarded as a working model where additional mechanistic aspects might get more relevant, if the substitution pattern of the MoQ scaffold is appropriately adapted.

S5.3 Regioselectivity of TGC formation

The regioselectivity of the TGC formation was confirmed by reacting mMoQ with an excess of butane thiol in MeOH until full conversion was observed via UHPLC. Precipitation and drying of the product were directly followed by NMR measurements without any further purification (**Figure S 23**).

The formation of the TGC adduct was confirmed via 2D-NMR spectroscopy. A coupling between the alpha CH_2 group of butane thiol (^{1}H: 2.60 ppm, red) to two different aliphatic carbons at 30.8 ppm and 21.3 ppm (beta and gamma carbon atom of butane thiol, light green) as well as to an aromatic carbon (^{13}C: 125.9 ppm, dark green) can be observed (**Figure S 23 A**). This aromatic carbon atom is quaternary as it does not couple to any proton in the ^{1}H-^{13}C HSQC spectrum, thus confirming the formation an alky aryl sulfide. To confirm the regioselectivity of this thiol addition the multiplicity as well as the coupling of the remaining two aromatic carbons of the TGC group have to be considered. A 2' thiol addition would result in a strong coupling of the protons in 5' and 6' position, therefore showing two doublet signals. This possibility can be excluded, as two singlets are observed. Addition in 5' position would result in a configuration where the protons in 2' and 6' position both couple to the central carbon atom at 52.7 ppm. However only one of the aromatic protons shows this coupling in the ^{1}H-^{13}C-HMBC spectrum (**Figure S 23 B**, dark blue to yellow). Therefore, the addition has to take place in 6' position, resulting in the two aromatic protons of the TGC groups being observed as singlets due to their para position relative to each other. On top of that only the proton in position 2' exhibits a ^{3}J ^{1}H-^{13}C coupling to the central carbon atom at 52.7 ppm in the HMBC spectrum, whereas the ^{4}J coupling starting from the aromatic proton in position 5' is not observed.

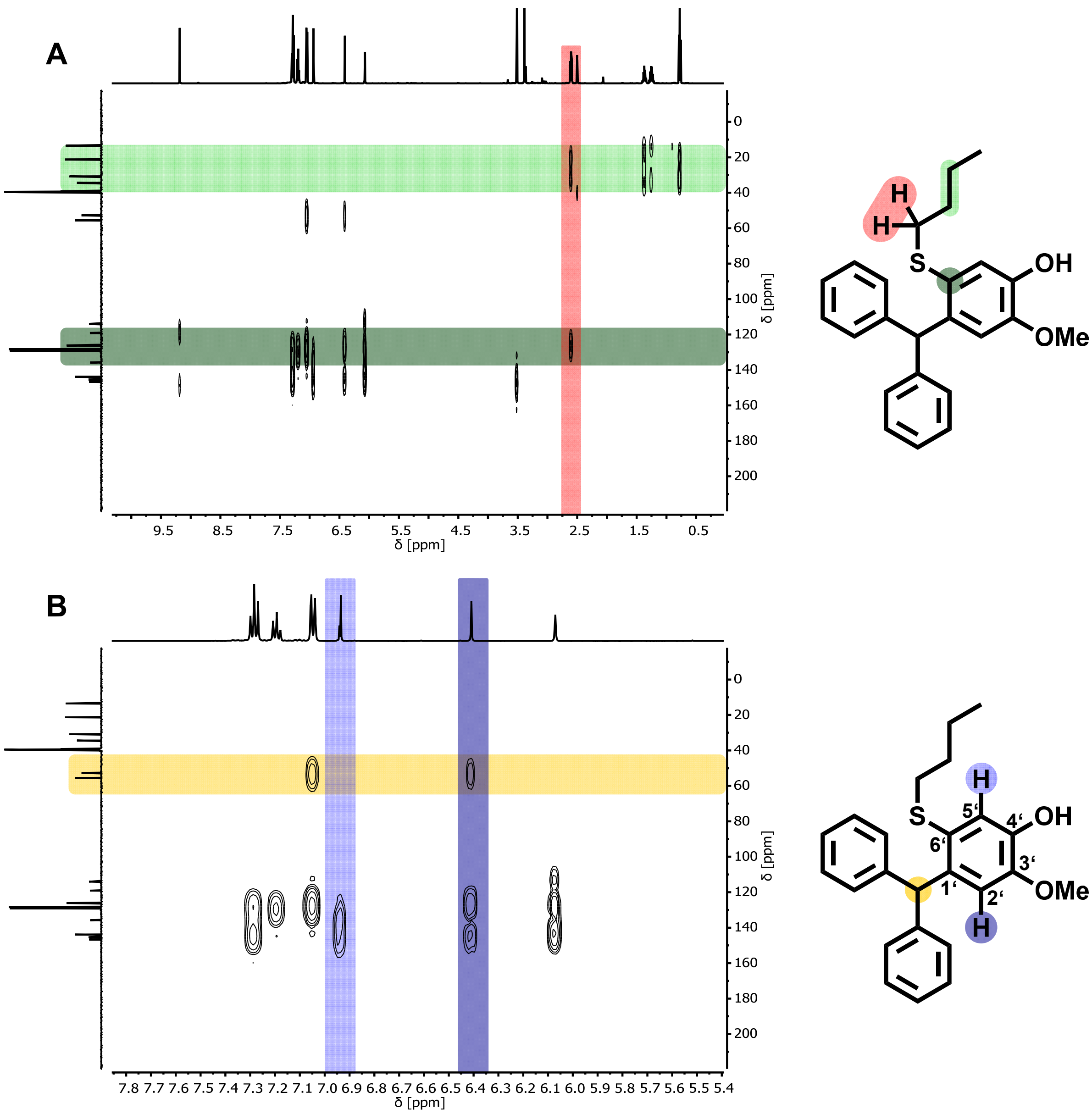

Figure S 23. ^{1}H-^{13}C-HMBC spectrum of the TGC adduct of butane thiol and mMoQ confirming the formation of an alkyl aryl sulfide (A) and zoom into the aromatic region to confirm the 6' addition position (B).

S5.4 TGC polymers

Polymerizations were carried out by reacting tMoQ and EDMP (MoQ/T = 3/2) at different concentrations according to **Table S 8** in methanol for 24 h at room temperature. Work-up of the polymer samples consisted of simple precipitation in MQ water and lyophilization to remove the solvent but retain unreacted tMoQ to get a rough estimate of monomer conversion. The data in **Table S 8** only considers the newly formed polymer fractions while ignoring unreacted tMoQ, as this could be removed by optimized precipitation conditions.

Table S 8. Polymerization of tMoQ with EDMP (MoQ/T = 3/2) at different functional group concentrations in MeOH and the resulting number average molar mass M_n, peak molar mass M_p, mass average molar mass M_w and polydispersity index *Đ* as confirmed by THF GPC analysis.

concentration	M_n [g/mol]	M_p [g/mol]	M_w [g/mol]	*Đ*
0.1 M	1500	1300	1800	1.21
0.25 M	1600	1300	2000	1.28
0.5 M	1700	1300	2400	1.43
1.0 M	2000	1300	3300	1.63
2.0 M	2300	1300	4000	1.72
bulk	1900	1300	3400	1.77

When comparing the ^{1}H-NMR spectra of these polymers to a structurally closely related monomer (tMoQ, twice substituted with $mPEG_3SH$ while leaving one MoQ group unreacted, compare **Figure S 19**), the presence of unreacted MoQ groups becomes evident (**Figure S 24**). A quantification of MoQ- to TGC-group conversion for this polymerization can be done by comparing the integrals of the central proton of tMoQ (5.1-5.4 ppm for this polymer) to the integral of the phenolic OH-groups of the formed TGC-units (9.0-9.5 ppm for this polymer). This results in a ratio of 1 to 2.1, corresponding to on average 2.1 TGC-units present per trimer unit, or 70% conversion. This is higher than the theoretically possible 2 TGC-units per tMoQ molecule for the here employed monomer ratio of MoQ/T = 3/2, as the precipitation of this polymer sample likely preferentially removes the smaller fragments (unreacted tMoQ or dimers), thus increasing the measured conversion.

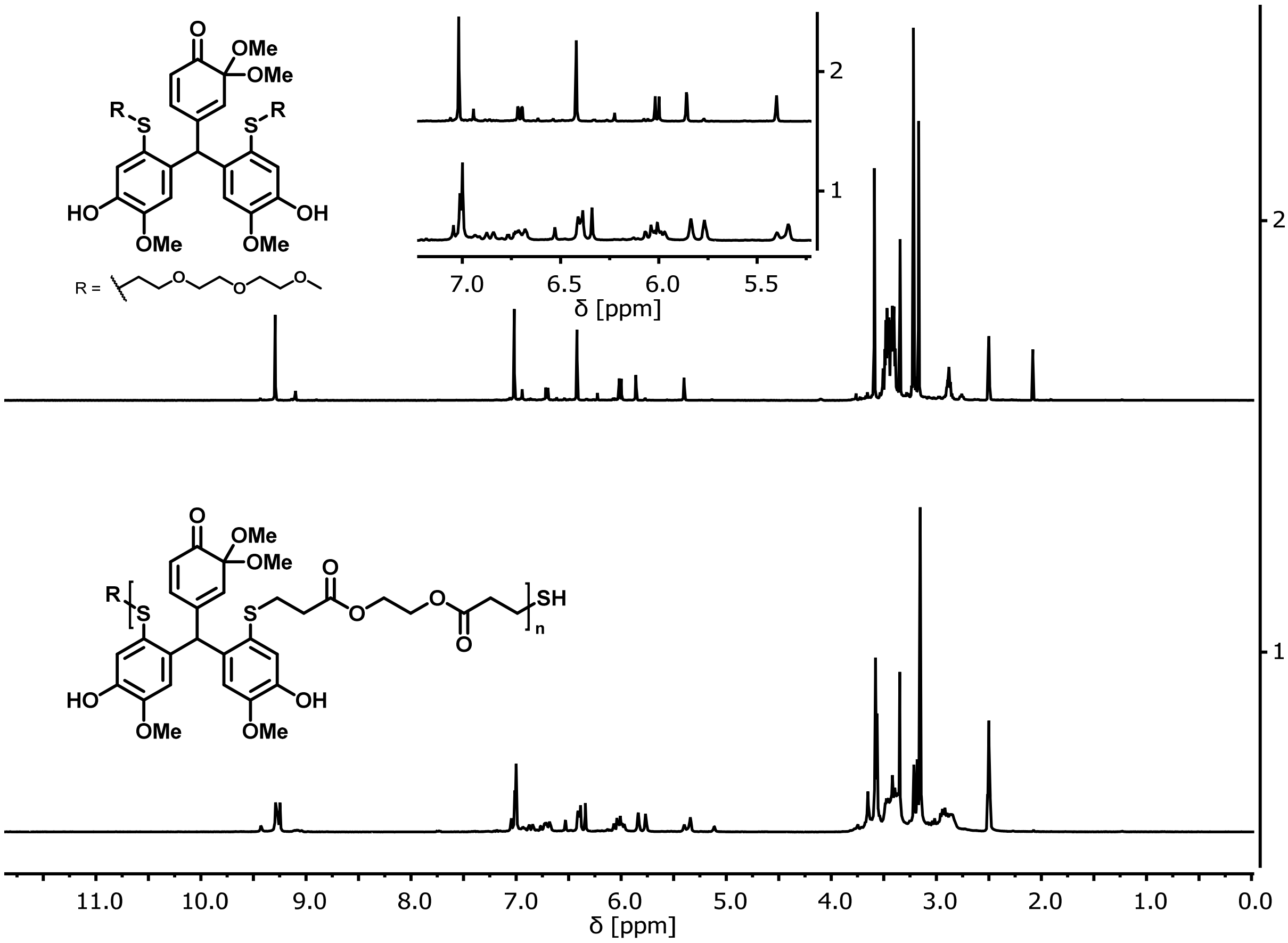

Figure S 24. Comparison of the ^{1}H-NMR spectra of the reaction product of tMoQ with $mPEG_3SH$ (MoQ/T = 1/1) after a reaction time of 6 h in MeOH (0.1 M) (top) and the reaction product of tMoQ with EDMP (MoQ/T = 3/2) after a reaction time of 24 h in MeOH (0.1 M) (bottom) and precipitation in MQ water (twice), measured in DMSO-d_6.

S5.5 tMoQ thermal stability

The thermal stability of tMoQ was investigated by TGA under argon (**Figure S 25 A**) as well as isothermal stability tests under atmospheric conditions (**Figure S 25 B, C, D, E**). TGA analysis revealed that no degradation takes place until 190 °C ($T_{99\%}$). A maximum degradation rate at 240 °C is observed, before a continuous degradation until full combustion occurs. Storing samples of tMoQ at different temperatures under atmospheric conditions for 16 hours is a better indicator for the stability in 2K adhesive mixtures. ^{1}H-NMR analysis (**Figure S 25 B**). revealed that tMoQ is stable up to a temperature of 100 °C with the only noticeable difference being the removal of residual solvents such as cyclohexane. Thermal degradation started at 110 °C, where tMoQ still remains stable for at least 8 hours (**Figure S 25 C**), and drastically increased at 120 °C. GPC measurements (**Figure S 25 E**) show a high molecular weight shoulder with increasing intensity alongside an increase in temperature, likely caused by *Diels-Alder* crosslinking. This was confirmed via FTIR measurements (**Figure S 25 D**), where the characteristic carbonyl vibration band of the alpha dimethoxy ketone in the DAC groups at 1737 cm^{-1} increases in intensity at higher temperatures with a gradual decline of the initially present MoQ carbonyl vibration at 1678 cm^{-1}. However, even elongated storage of tMoQ at 120 °C under atmospheric conditions only resulted in a partially crosslinked but still fully soluble product. This is likely caused by the drastically increased steric demand after DA dimerization, limiting further reactivity.

In addition, tMoQ also exhibits excellent stability under ordinary laboratory storage conditions. According to ^{1}H NMR spectroscopy tMoQ remains unchanged for at least 5 months when stored under ambient conditions (23 °C, air, dark storage), as no differences are detectable (**Figure S 26**).

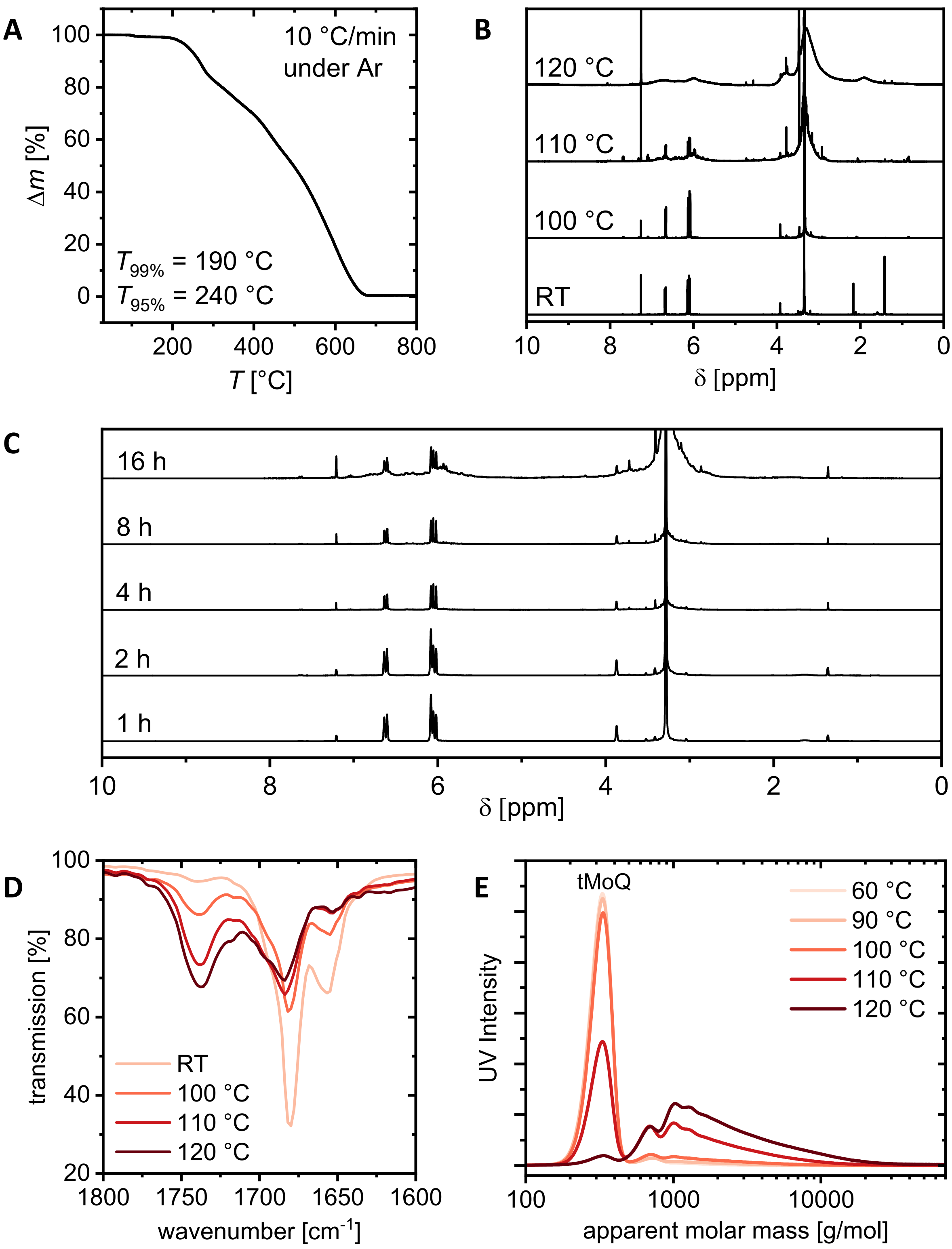

Figure S 25. TGA measurement of tMoQ under an argon atmosphere with a heating rate of 10 °C/min (A), as well as ^{1}H-NMR-spectra in $CDCl_3$ after storing tMoQ under atmospheric conditions for 16 h at different temperatures (B) or at 110 °C for different times (C), FT-IR spectroscopy (D) and THF-GPC measurements (E) of tMoQ after 16 h at different temperatures under atmospheric conditions.

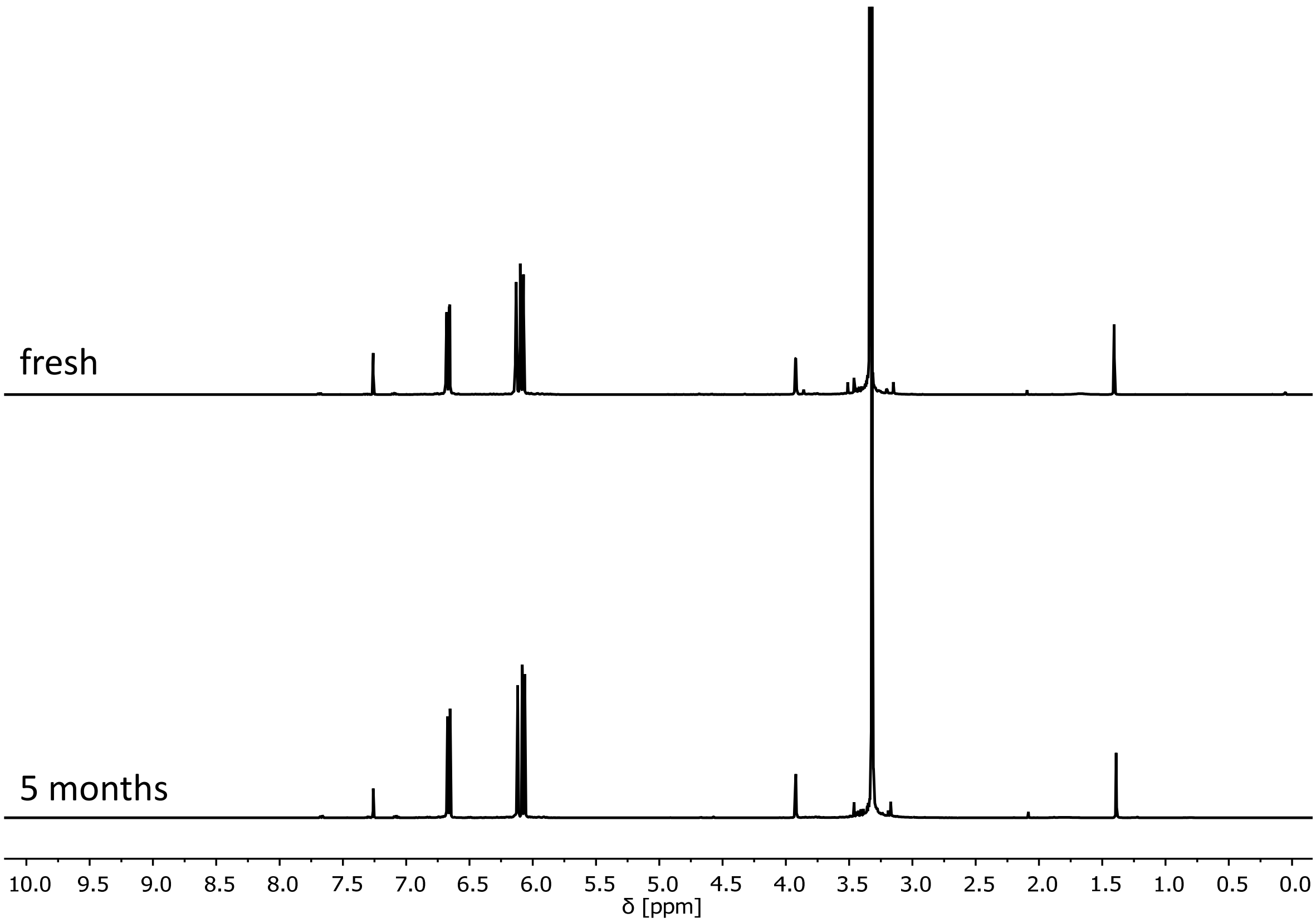

Figure S 26. *^{1}H-NMR spectrum of tMoQ in $CDCl_3$ before (top) and after (bottom) storing for 5 months at ambient conditions (23 °C, air, dark storage).*

S5.6 mMoQ *Diels-Alder* dimerization

The formation of the mMoQ Diels-Alder dimer was confirmed by 2D-NMR spectroscopy (^{1}H-^{13}C-HSQC and ^{1}H-^{13}C-HMBC) and the peaks of the compound were assigned accordingly (**Table S 9**).

Table S 9. ^{1}H and ^{13}C Chemical shift of the mMoQ Diels-Alder dimer.

Atom	^{1}H shift [ppm]	^{13}C shift [ppm]
1	/	192.6
2	6.06	129.5
3	6.90	148.8
4	/	53.0
5	4.24	61.4
6	3.27	45.5
7	/	97.1
8/9	3.23/2.72	49.1/48.9
10	3.06	44.4
11	/	150.1
12	4.62	56.7
13	5.33	125.7
14	3.07	57.5
15	/	200.9
16	/	94.3
17/18	3.49/3.52	50.5/48.5

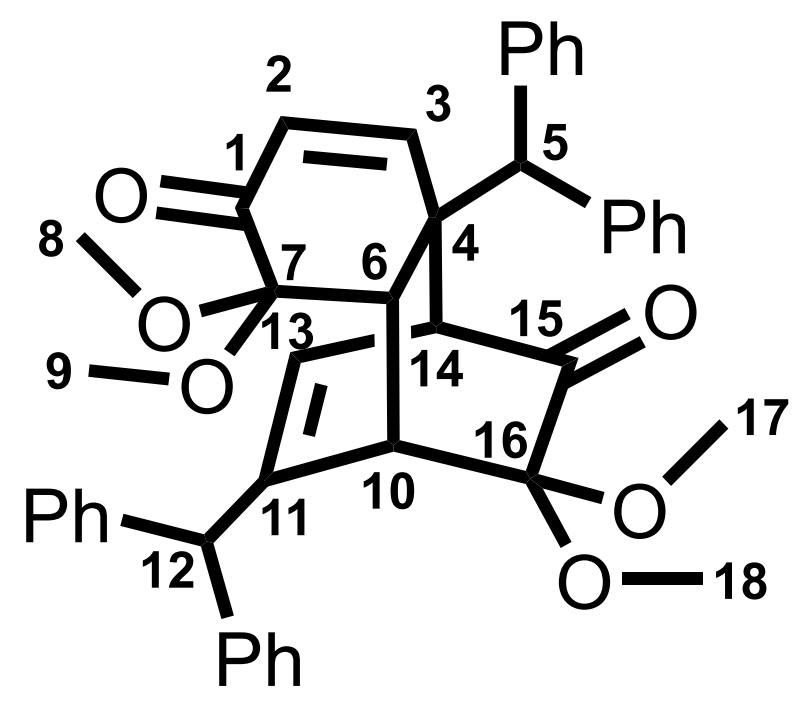

This dimer was also investigated in regards to its reactivity with a monothiol (ethane thiol). Even with the addition of 10 mol% triethyl amine as a base catalyst, no conversion could be observed after 24 h in solution (**Figure S 27**). It was therefore concluded that DAC groups in general are unreactive to thiols, excluding their participation in network formation for the bulk reaction of tMoQ with thiols.

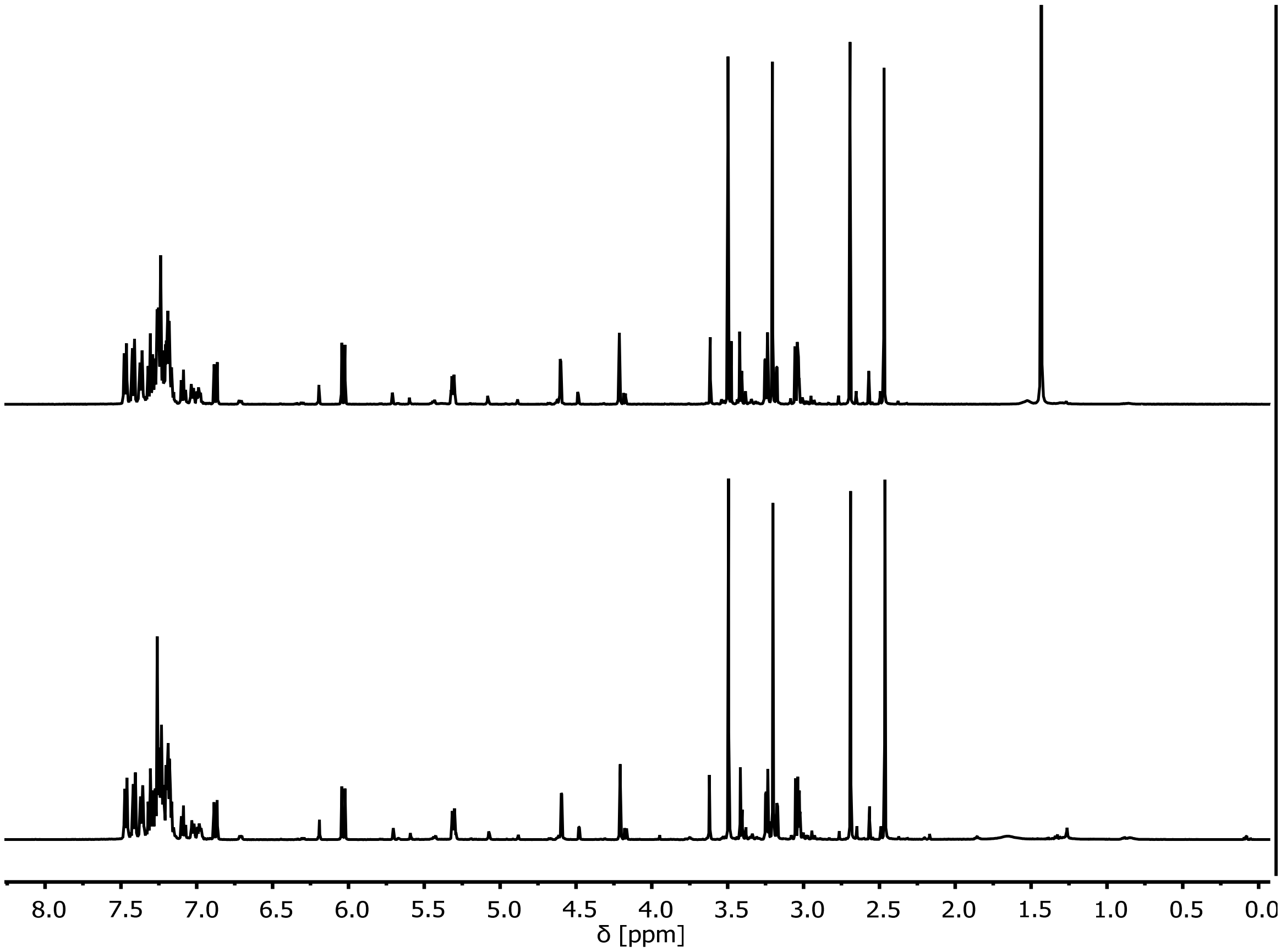

Figure S 27. ^{1}H-NMR spectrum of mMoQ-DAC before and after reaction with ethane thiol (10 eq) and triethyl amine (10 mol%) in NMP, measured in $CDCl_3$.

S5.7 *Diels-Alder* vs TGC in bulk (mMoQ)

For preparation of bulk mixtures see **Section S5.9**. When reacting mMoQ with $mPEG_3SH$ (MoQ/T = 1/1) under bulk conditions the trends of increasing MoQ conversion as well as DAC formation with an increase in temperature was observed. UHPLC-ESI-MS measurements were used to quantify the amount of DAC product in the reaction mixture (**Figure S 28**). As mMoQ and the mMoQ-$mPEG_3SH$ TGC adduct could not be chromatographically separated, additional ^{1}H-NMR analysis of the reaction mixtures was carried out to confirm mMoQ conversion (**Figure S 29**). The amount of unreacted mMoQ in relation to TGC product was determined to be 48% at 30 °C and 35% at 60 °C. At 90 °C only traces of mMoQ could be observed, whereas 120 °C resulted in a full mMoQ conversion. Representative data for the reaction at 30 °C are shown for both UHPLC and ^{1}H-NMR analysis.

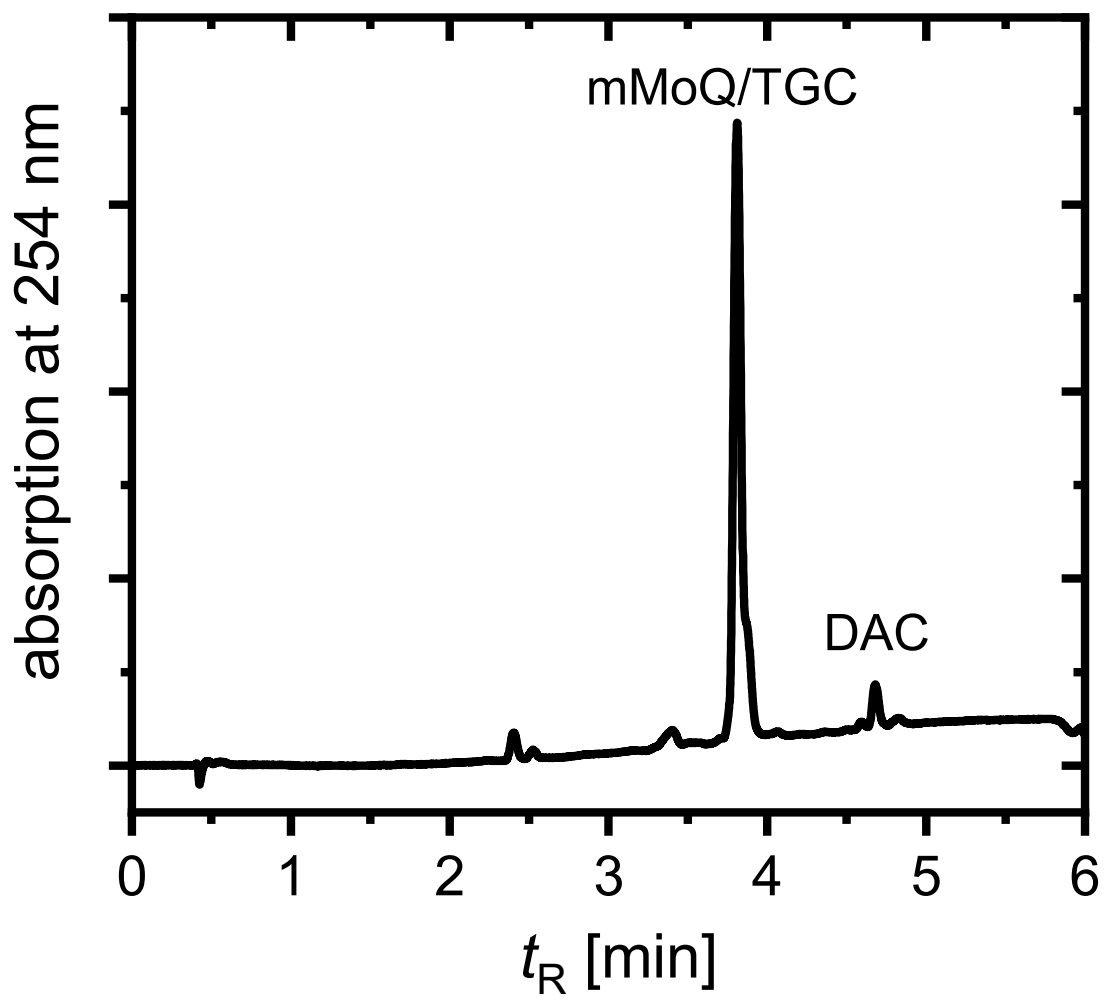

Temperature [°C]	Amount of DAC
30	3%
60	8%
90	14%
120	28%

Figure S 28. UHPLC-ESI-MS measurement (λ = 254 nm, gradient 10-90% B with A: H2O + 0.1% FA and B: ACN + 0.1% FA) of the reaction product of mMoQ and $mPEG_3SH$ (MoQ/T = 1/1) after 16 h at 30 °C as well as the resulting amount of DAC as a percentage of all reaction products when carrying out the reaction at different temperatures.

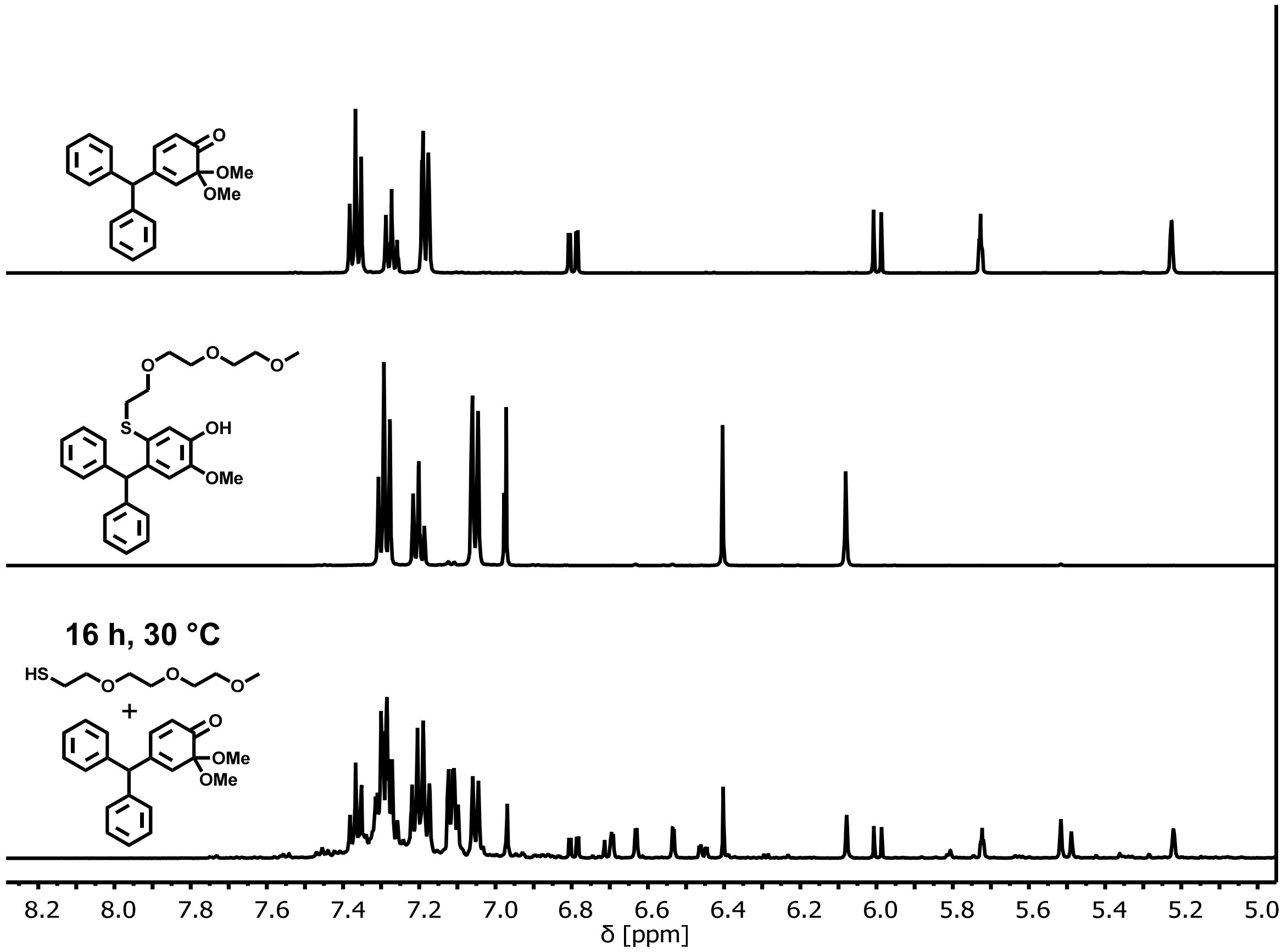

Figure S 29. Stacked ^{1}H-NMR spectra of mMoQ (top), mMoQ-mPEG$_3$SH TGC adduct (middle) and the reaction product of mMoQ and mPEG$_3$SH (MoQ/T = 1/1) after 16 h at 30 °C (bottom) in DMSO-d$_6$.

S5.8 Bulk reactions of tMoQ

The reaction of tMoQ with mPEG$_3$SH (MoQ/T = 1/1) under bulk conditions could be verified using UHPLC-ESI-MS analysis (**Figure S 30**). Representative data of the reaction at 30 °C is shown, where a mixture of mono and di addition products is observed. GPC analysis of these bulk reactions indicated the di addition product as the main product, with peak molecular weights M_p between 720 and 800 g/mol being observed for all temperatures (**Figure S 31**, 2-TGC: 767 g/mol). Additionally, a higher molecular weight shoulder is observed, increasing in intensity at higher temperatures. This shows an increasing degree of Diels-Alder crosslinking, achieving apparent molecular weights of up to 10^4 g/mol when the reaction is carried out at 120 °C.

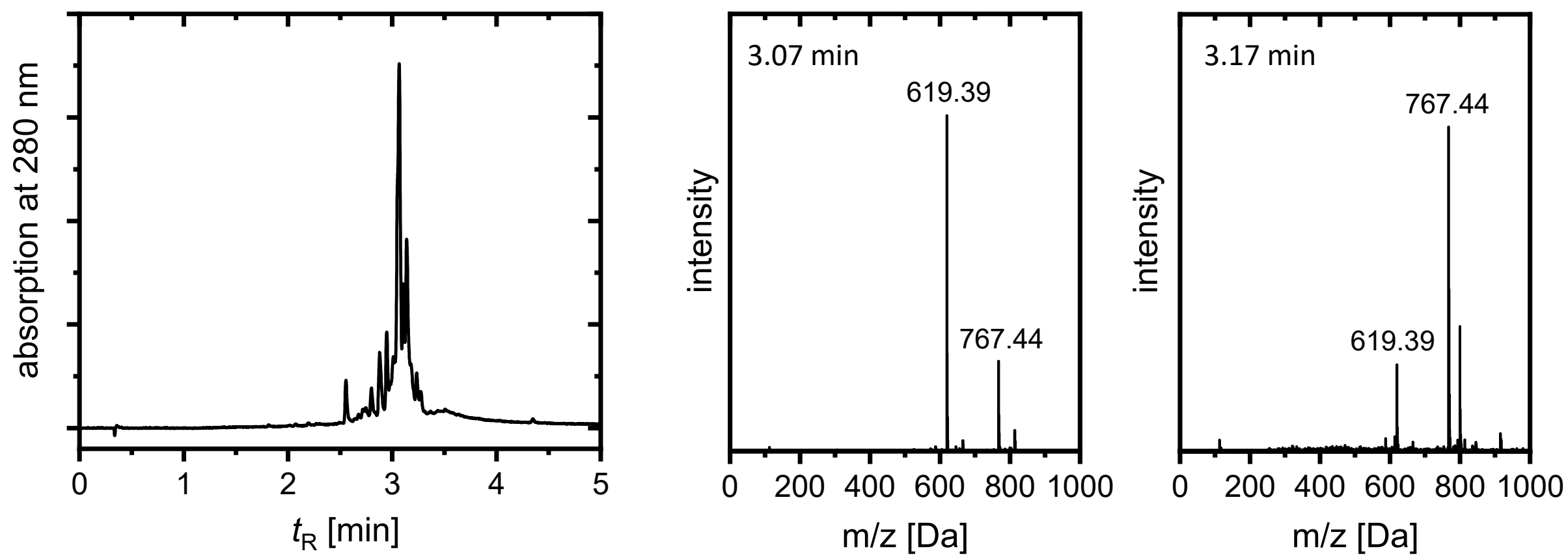

Figure S 30. UHPLC-ESI-MS analysis of the bulk reaction between tMoQ and mPEG$_3$SH (MoQ/T = 1/1) at 30 °C after 24 h (left) and the mass spectra at 3.07 min (middle) and 3.17 min (right). 1-TGC: calculated (m/z): 619.23 $[M-H]^-$, found (m/z): 619.39 $[M-H]^-$; 2-TGC: calculated (m/z): 767.28 $[M-H]^-$, found (m/z): 767.44 $[M-H]^-$.

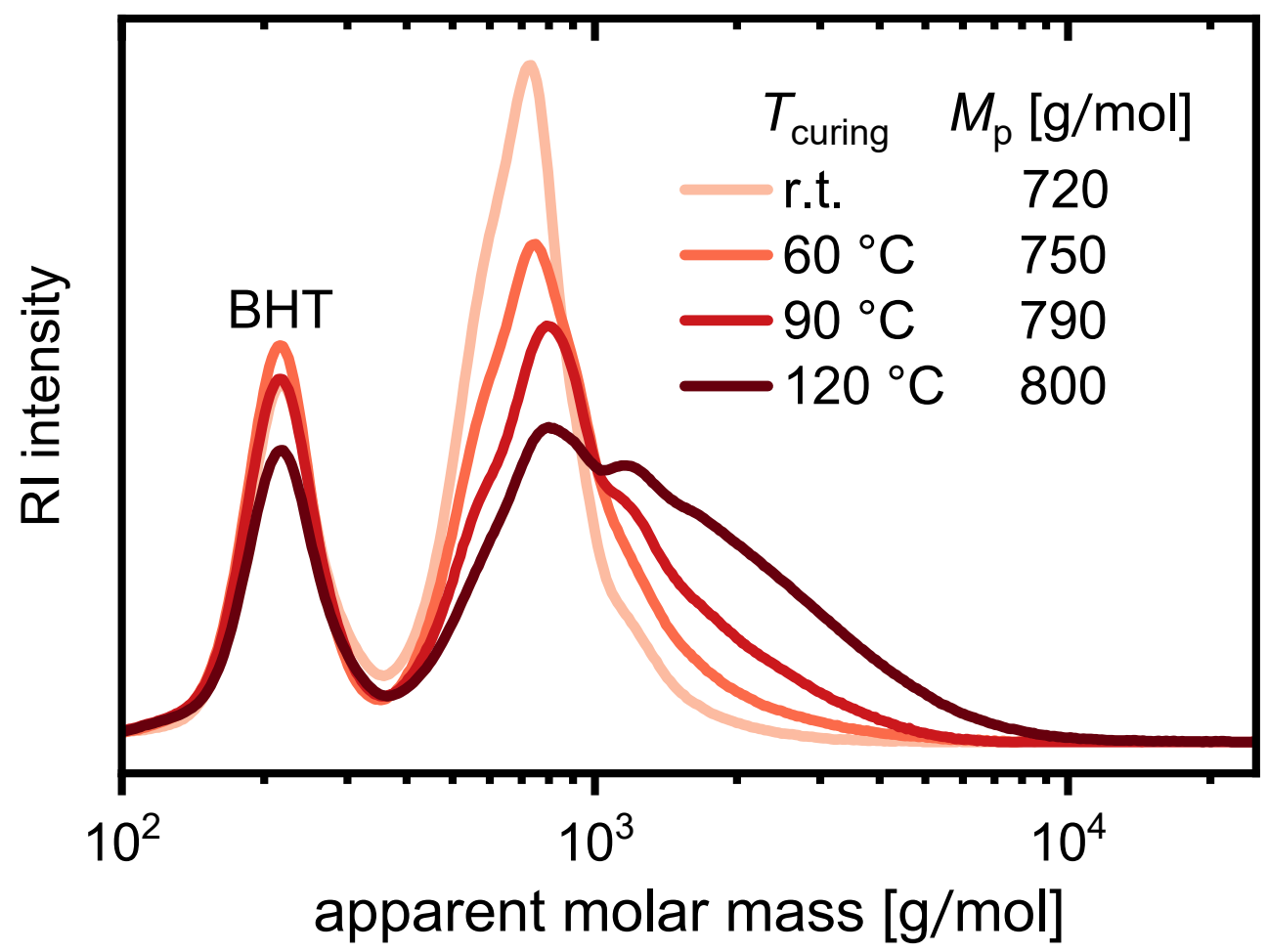

Figure S 31. Bulk reaction of tMoQ (470 g/mol) with mPEG$_3$SH (180 g/mol) for 24 h at different temperatures analyzed by THF-GPC with BHT as internal standard.

S5.9 Preparation of 2K mixtures

The 2K mixtures were prepared by adding the solid component (mMoQ, dMoQ, tMoQ or oxidized lignin) and the corresponding liquid thiol ($mPEG_3SH$, EDMP, TTMP, PTMP, DHMP, GPM800 or $ETTMP_{1300}$) to a 10 mL polypropylene container in the corresponding ratio. The container was then placed in a mixer for bladeless shear mixing (SpeedMixer) and the compounds were blended using a mixing ramp from 1000 rpm to 3500 rpm for a duration of 2 minutes and 40 seconds (1500 rpm for 20 s, 2000 rpm for 20 s, 2500 rpm for 20 s, 3000 rpm for 40 s and 3500 rpm for 60 s), resulting in a homogeneous viscous paste. In case of lap shear strength testing the 2K mixtures were applied to an aluminum substrate (cleaned with acetone prior to use) with roughly 10 mg being used on a 60 mm² (3 mm x 20 mm) surface area. A second substrate was then added on top of the applied adhesive, creating an overlap between the two substrates. Both substrates were fixed together, and the expelled glue was removed prior to curing in an oven at a constant temperature for 16 h. Afterwards, the samples were equilibrated at room temperature for 1 h prior to testing. The shear strength was calculated as the maximum force recorded before failure divided by the adhesive contact area between the two aluminum substrates and is reported in $N \cdot mm^{-2}$ (equivalent to Pa).

S5.10 DAC formation during 2K adhesive curing

The formation of DAC during the curing process was investigated through the use of GPM800 (**Figure S 32**), which does not contain any ester bonds, which would overlap with the newly formed DAC carbonyl bands. Even curing temperatures as high as 150 °C do not seem to cause the formation of any DAC, shown by the absence of the characteristic DAC IR bands (**Figure S 33**). It is therefore likely that DAC does not occur in any significant amount in any of the other 2K mixtures that were employed here.

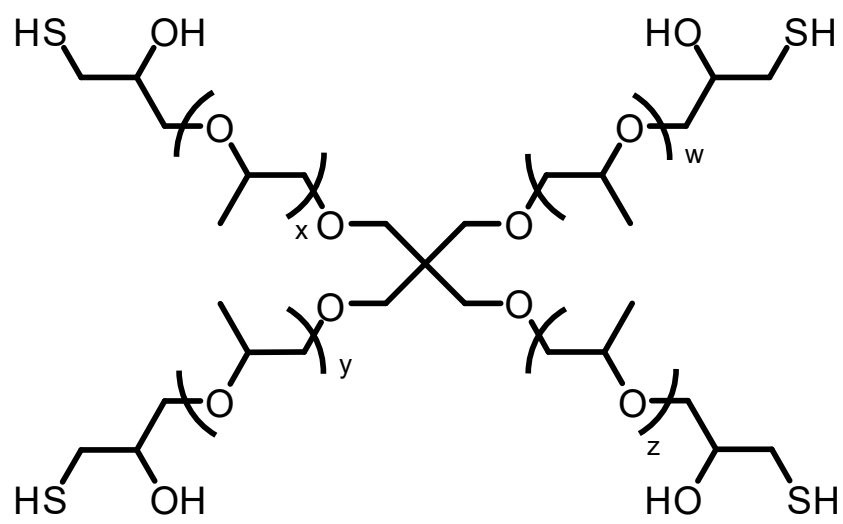

Figure S 32. Chemical structure of the tetrathiol GPM800, with an average molecular weight of M_n = 800 g/mol (w+x+y+z = 5).

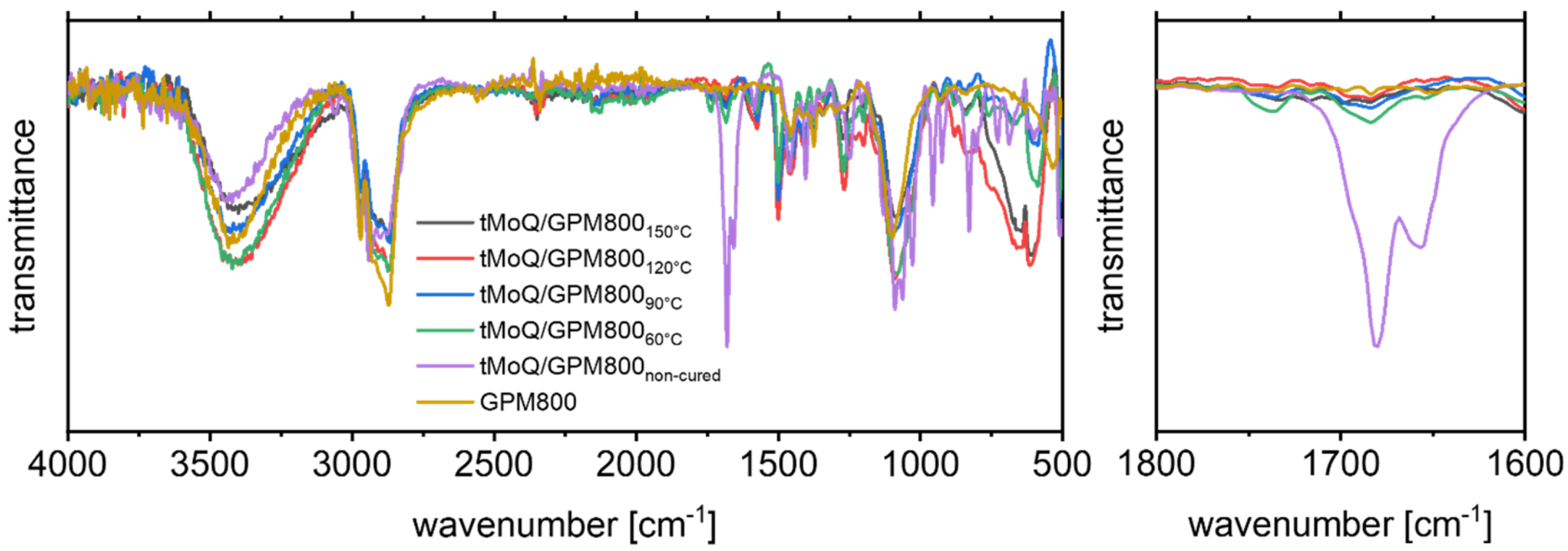

Figure S 33. FT-IR spectrum of 2K mixtures of tMoQ and GPM800 (MoQ/T = 1/0.6), non-cured as well as cured for 16 h at 60 °C, 90 °C, 120 °C and 150 °C.

S5.11 Characterization of thiol crosslinkers for 2K application

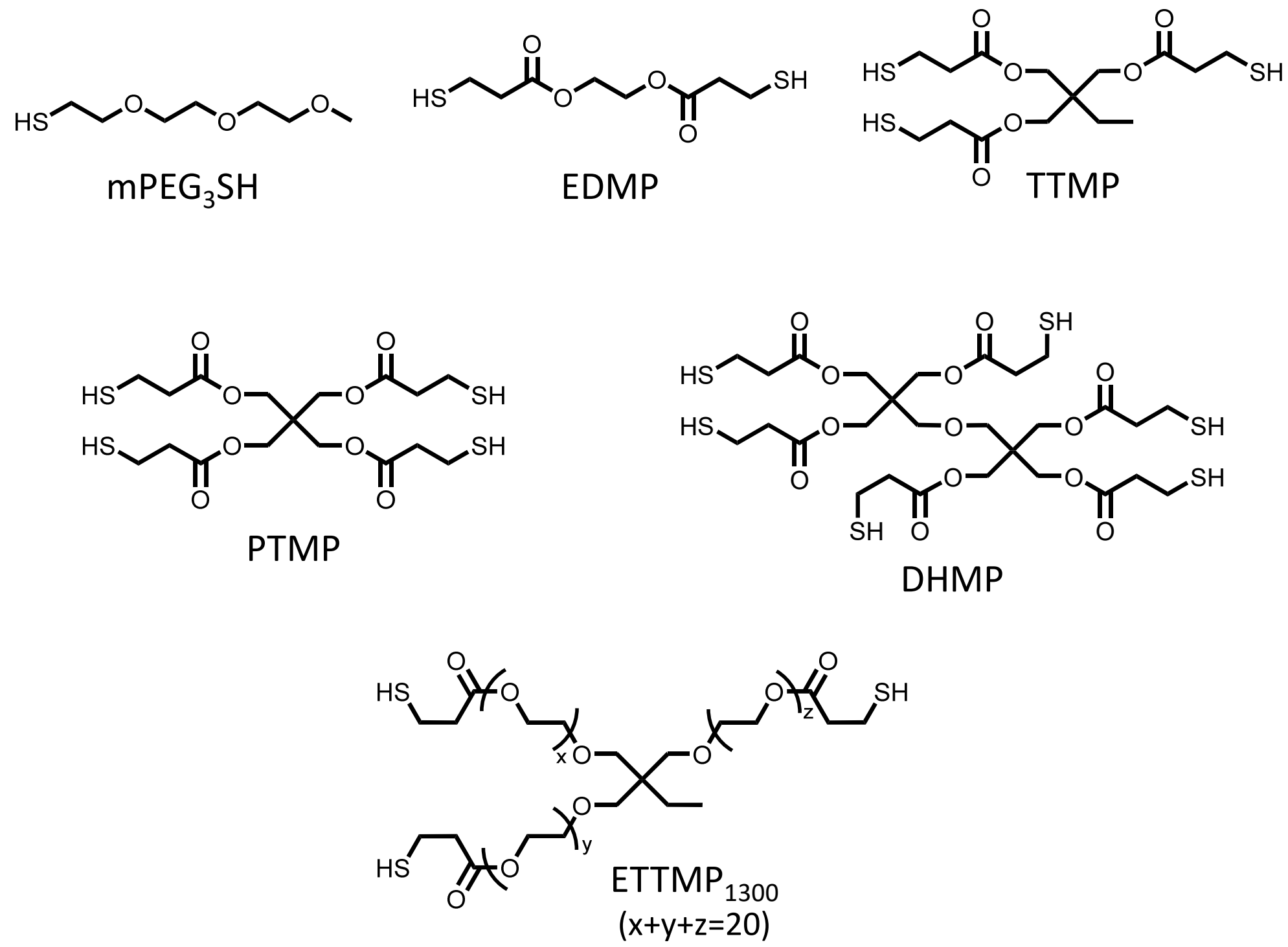

Figure S 34. Chemical structure of the thiols $mPEG_3SH$, EDMP, TTMP, PTMP, DHMP and $ETTMP_{1300}$.

To determine the viscosities, flow curves were recorded in rotational tests on the rheometer at 25 °C (**Table S 10**). Shear thinning was observed for all thiols.

Table S 10. Characterization of the different liquid thiols used in this study.

Thiol	SH-groups per molecule	Molecular weight [g/mol]	Thiol density [mmol/g]	Viscosity [mPa·s] at 10 s^{-1} shear rate
$mPEG_3SH$	1	180.26	5.55	5
EDMP	2	238.32	8.39	21
TTMP	3	398.55	7.53	164
PTMP	4	488.64	8.19	448
DHMP	6	783.02	7.66	2354
$ETTMP_{1300}$	3	1300 (M_n)	2.31	425

S5.12 Rheology measurements

The curing behavior of the different 2K mixtures of tMoQ and the thiols EDMP, TTMP, PTMP and DHMP with a MoQ/T ratio of 1/1 was investigated via rheological analysis at 60 °C (**Figure S 35**). Additionally, the curing of tMoQ with DHMP was investigated at temperatures of 20 °C, 30 °C, 40 °C and 50 °C with all mixtures showing a sol-gel transition (**Figure S 36**).

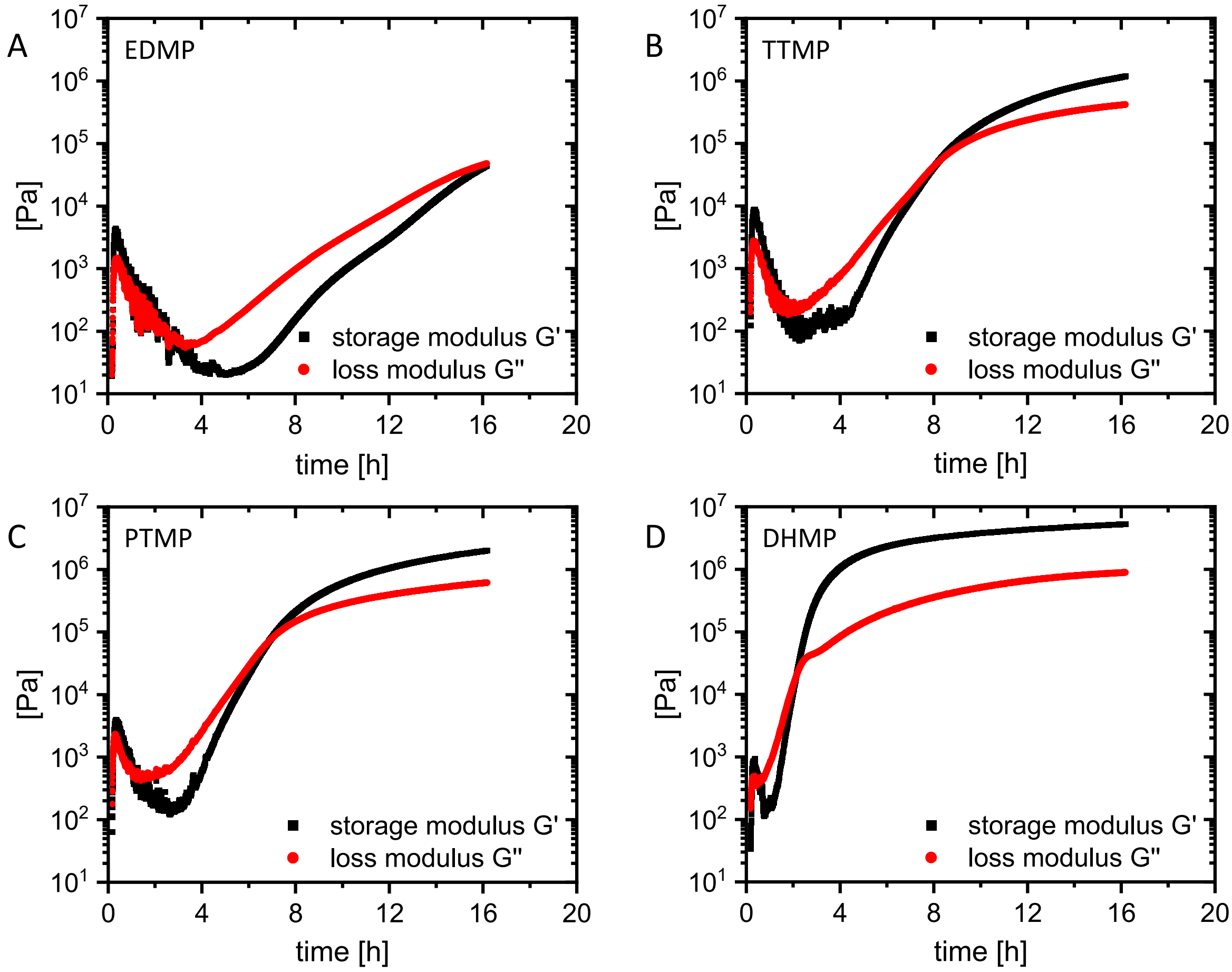

Figure S 35. Isothermal rheological analysis (60 °C, γ = 0.1%, ω = 10 rad·s^{-1}) of the different 2K mixtures consisting of tMoQ and multithiol (A: EDMP, B: TTMP, C: PTMP, D: DHMP) with a MoQ/T ratio of 1/1.

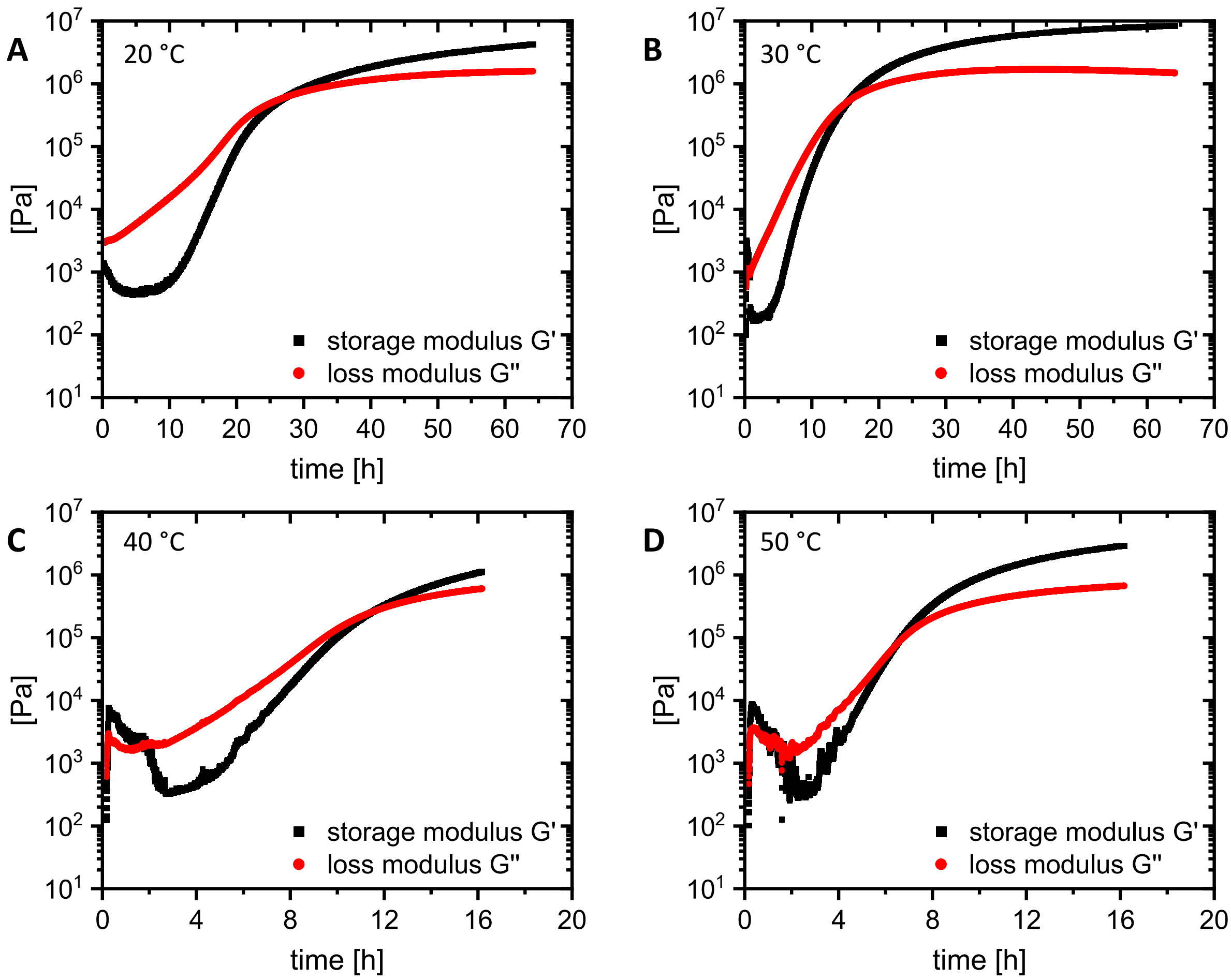

Figure S 36. Isothermal rheological analysis (γ = 0.1%, ω = 10 rad·s^{-1}) of the 2K mixtures consisting of tMoQ and DHMP with a MoQ/T ratio of 1/1 cured at 20 °C (A), 30 °C (B), 40 °C (C) and 50 °C (D).

The trend of tan δ (ratio of loss modulus G'' and the storage modulus G') during the isothermal curing gives an insight into the underlying mechanisms. An ideal elastic solid would exhibit a tan δ value of 0, whereas an ideal viscous liquid would exhibit a tan δ value approaching infinity. The tMoQ-multithiol mixtures all initially behave as viscoelastic solids with tan δ < 1 (**Table S 11**). During the heating process tan δ rapidly increases as the 2K mixtures melt, as shown exemplarily for PTMP in **Figure S 37 B**. After that a continuous decrease in tan δ can be observed, followed by the first sol-gel transition. This is likely caused by the increased solubility of tMoQ in the multithiols at increased temperatures, causing more tMoQ to dissolve over time. The TGC formation during the curing process causes methanol to be released into the 2K mixture, causing a gel-sol transition and turning the material increasingly more liquid. At a certain point the network formation takes over and the mixture begins to harden, as confirmed by the maximum of the tan δ curves. Eventually all 2K mixtures except for EDMP exhibit a second sol-gel transition. Multithiols with more thiol groups per molecule cause this network formation to be faster, as fewer coupling steps are necessary to reach the same crosslinking density. Therefore, the more thiol groups per crosslinker the faster the curing takes places, as seen by the earlier tan δ peaks and earlier second sol-gel transitions (**Table S 11**).

Table S 11. sol-gel and gel-sol transitions as well as selected tan δ and G' values for the isothermal rheological analysis (60 °C, 16 h, γ = 0.1%, ω = 10 rad·s^{-1}) of the different 2K mixtures consisting of tMoQ and EDMP, TTMP, PTMP or DHMP with a MoQ/T ratio of 1/1.

Thiol	initial tan δ	sol-gel 1	gel-sol	tan δ peak	sol-gel 2	final tan δ	final G' (20 °C)
EDMP	0.734	13 min	123 min	6.5 h	/	0,128	7.7 • 10^6
TTMP	0.271	12 min	92 min	4.5 h	8.1 h	0,046	1.0 • 10^7
PTMP	0.285	14 min	69 min	3.4 h	6.9 h	0,017	1.1 • 10^7
DHMP	0.404	14 min	32 min	1.1 h	2.1 h	0,010	1.1 • 10^7

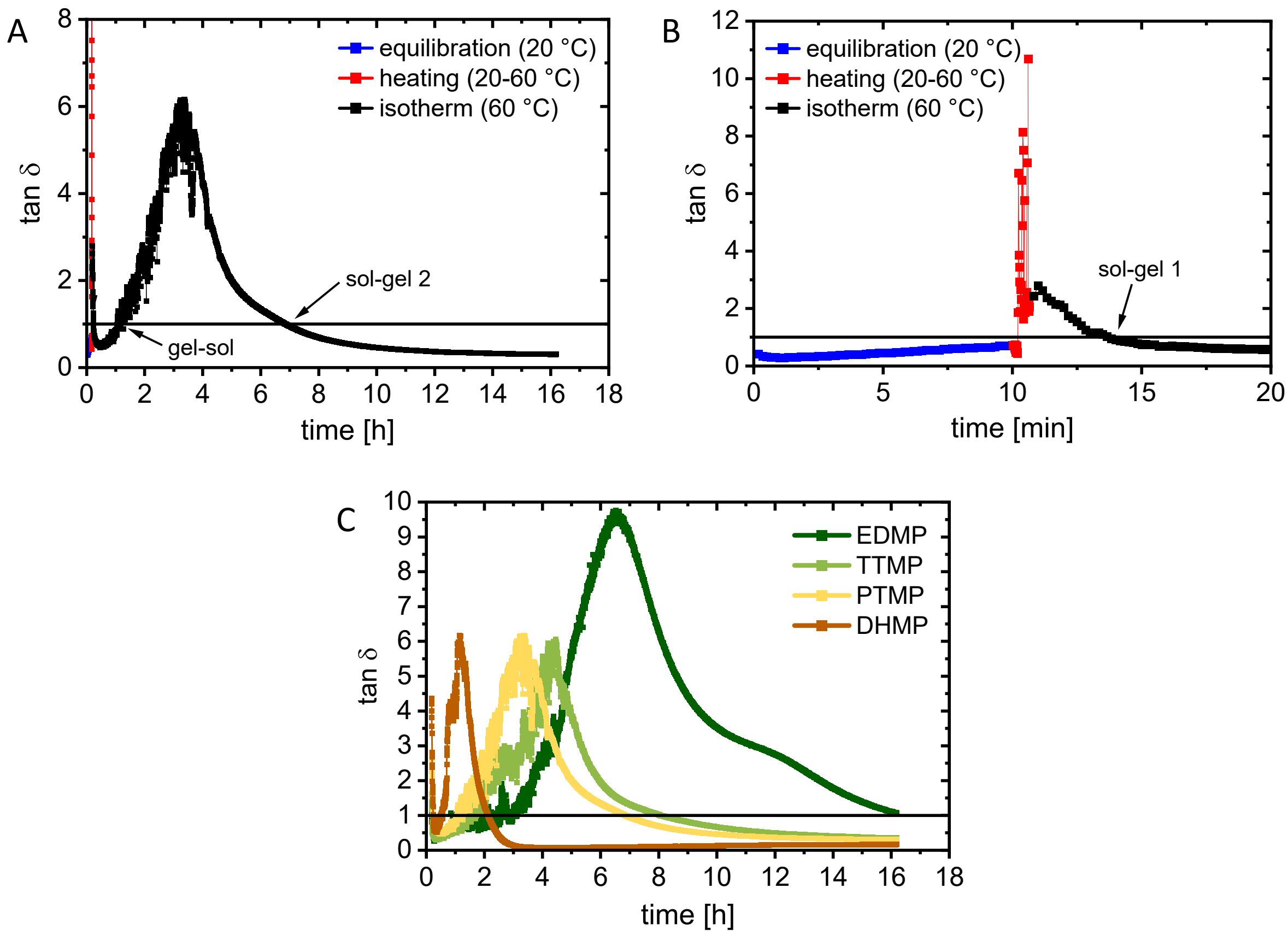

Figure S 37. Exemplary trend of tan δ during isothermal rheological analysis (60 °C, γ = 0.1%, ω = 10 rad·s^{-1}) of the tMoQ/PTMP 2K mixture (MoQ/T = 1/1) over a period of 16 hours (A) as well as the first 20 minutes (B) and the different 2K mixtures consisting of tMoQ and EDMP, TTMP, PTMP or DHMP with a MoQ/T ratio of 1/1 (C) with the black line showing the sol-gel crossover points at tan δ = 1.

S5.13 Analysis of cured 2K adhesives

Generally, three bonded specimens were prepared for each test series, and their adhesive strengths were determined using lap shear testing. Based on the individual measured values, the mean and standard deviation were calculated. Results are reported as mean ± standard deviation. To assess the significance of differences between various measurements, statistical significance was determined using two-sample Student's t-tests. Significance levels are indicated as follows: $p \geq 0.05$ (not significant, n.s.), $p < 0.05$ (*), $p < 0.01$ (**), $p < 0.001$ (***). All statistical analyses were performed using OriginPro 2024 (Version 10.1.0.178).

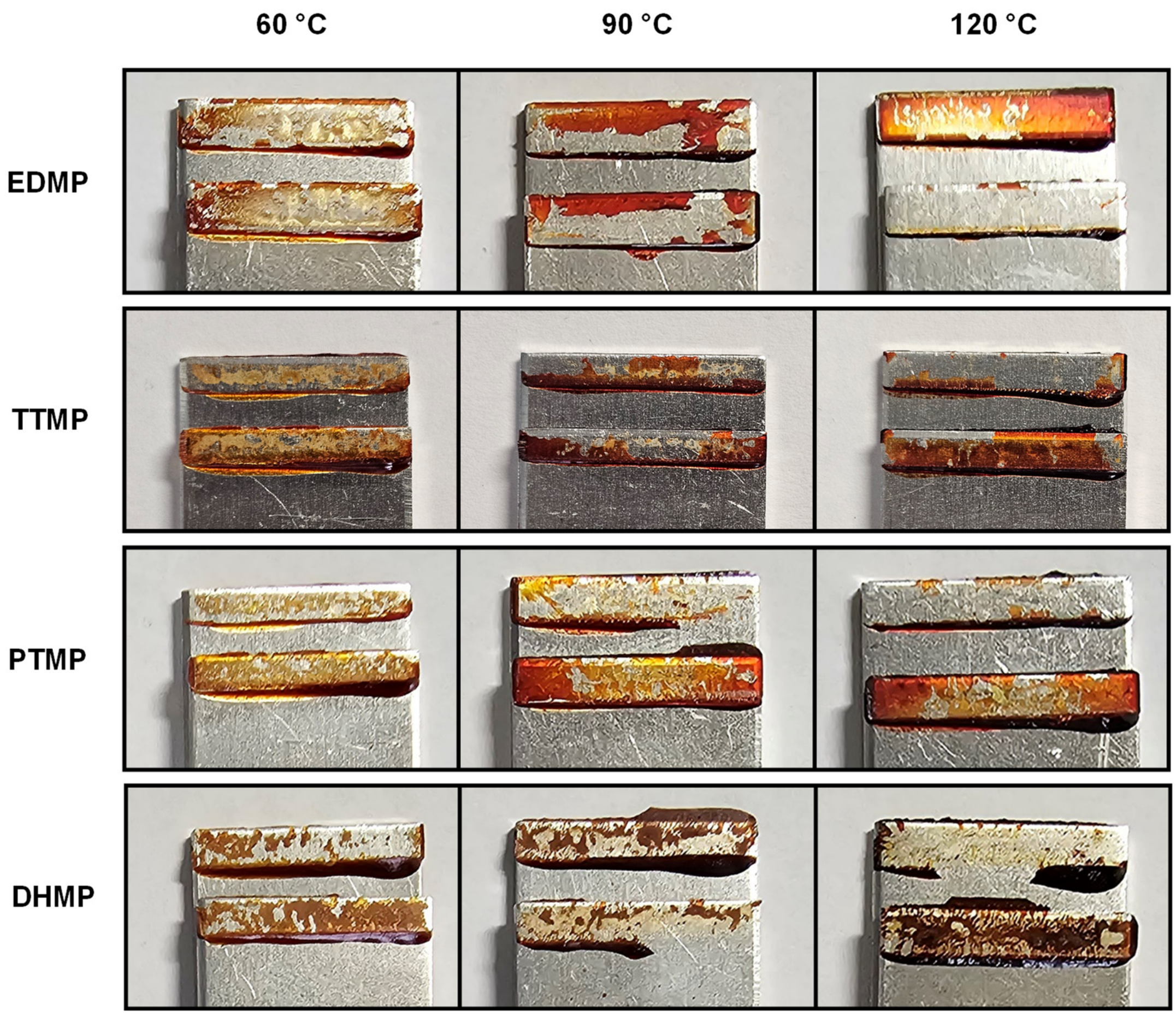

Figure S 38. Fracture patterns of the different 2K adhesives consisting von tMoQ and EDMP, TTMP, PTMP and DHMP (MoQ/T = 1/1), cured for 16 h at 60 °C, 90 °C or 120 °C.

Table S 12. Glass transition temperatures and fracture patterns of 2K adhesives based on tMoQ and the multithiol crosslinkers EDMP, TTMP, PTMP, and DHMP. Formulations were prepared at MoQ/T = 1/1 and cured for 16 h at 60, 90, or 120 °C. Glass transition temperatures were determined by DSC at a heating rate of 10 °C min^{-1}.

Thiol crosslinker	Curing temperature (for 16 h)	Glass transition temperature T_g	Fracture pattern
EDMP	60 °C	20 °C	cohesive
	90 °C	48 °C	adhesive
	120 °C	49 °C	adhesive
TTMP	60 °C	72 °C	cohesive
	90 °C	72 °C	mixed
	120 °C	82°C	adhesive
PTMP	60 °C	83 °C	cohesive
	90 °C	109 °C	mixed
	120 °C	120 °C	adhesive
DHMP	60 °C	27 °C	mixed
	90 °C	96 °C	mixed
	120 °C	151 °C	adhesive

Table S 13. Statistical analysis of lap-shear strengths for 2K adhesives based on tMoQ and the multithiol crosslinkers EDMP, TTMP, PTMP, and DHMP (MoQ/T = 1/1, cured for 16 h at 60, 90, or 120 °C), comparing different formulations cured at the same temperature. Significance levels are indicated as follows: $p \geq 0.05$ not significant (n.s.), $p < 0.05$ (*), $p < 0.01$ (**), $p < 0.001$ (***).

comparison	curing temperature		
	60 °C	90 °C	120 °C
EDMP - TTMP	*	***	**
EDMP - PTMP	**	**	*
EDMP - DHMP	***	**	n.s.
TTMP - PTMP	*	n.s.	n.s.
TTMP - DHMP	*	**	*
PTMP - DHMP	n.s.	*	n.s.

Table S 14. Statistical analysis of lap-shear strengths for 2K adhesives based on tMoQ and the multithiol crosslinkers EDMP, TTMP, PTMP, and DHMP (MoQ/T = 1/1, cured for 16 h at 60, 90, or 120 °C), comparing the same formulation cured at different temperatures. Significance levels are indicated as follows: $p \geq 0.05$ not significant (n.s.), $p < 0.05$ (*), $p < 0.01$ (**), $p < 0.001$ (***).

	curing temperature		
	60 °C vs 90 °C	60 °C vs 120 °C	90 °C vs 120 °C
EDMP	***	*	n.s.
TTMP	**	*	n.s.
PTMP	n.s.	n.s.	n.s.
DHMP	*	**	n.s.

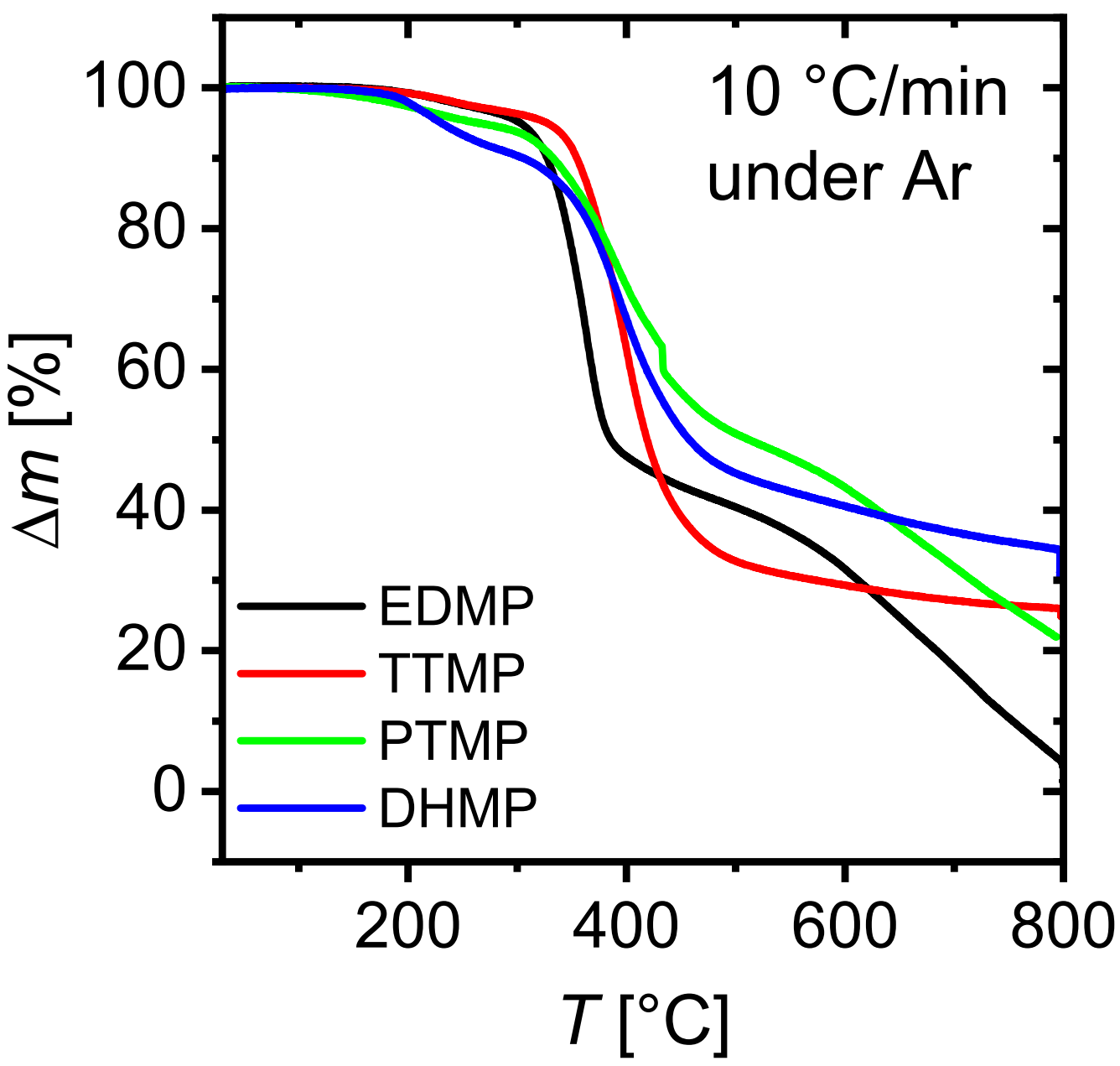

Figure S 39. Exemplary TGA analysis with a heating rate of 20 °C/min of the 2K systems from tMoQ with the given multi-thiols with a MoQ/T ratio of 1/1 after the mixtures were cured at 90 °C for 16 h.

To contextualize the performance of the tMoQ based adhesives, we performed additional lap-shear tests on aluminum, using commercially available adhesives and compared them with the best-performing MoQ-based formulation, tMoQ/TTMP (MoQ/T = 1/1, cured at 90 °C for 16 h). The commercial adhesives were applied and cured according to the manufacturers' instructions and tested under identical conditions. The tMoQ/TTMP adhesive reached a lap-shear strength of 16.9 MPa, closely matching a commercial 2K epoxy adhesive (UHU 2-K-Epoxidkleber, 16.0 MPa). It clearly outperformed a commercial poly(cyanoacrylate) adhesive (Pattex Sekundenkleber Mini Trio, 9.0 MPa) and a siloxane-based adhesive (Pattex Repair Extreme, 2.5 MPa). These results directly support the classification of the MoQ-based system as a high-performance adhesive.

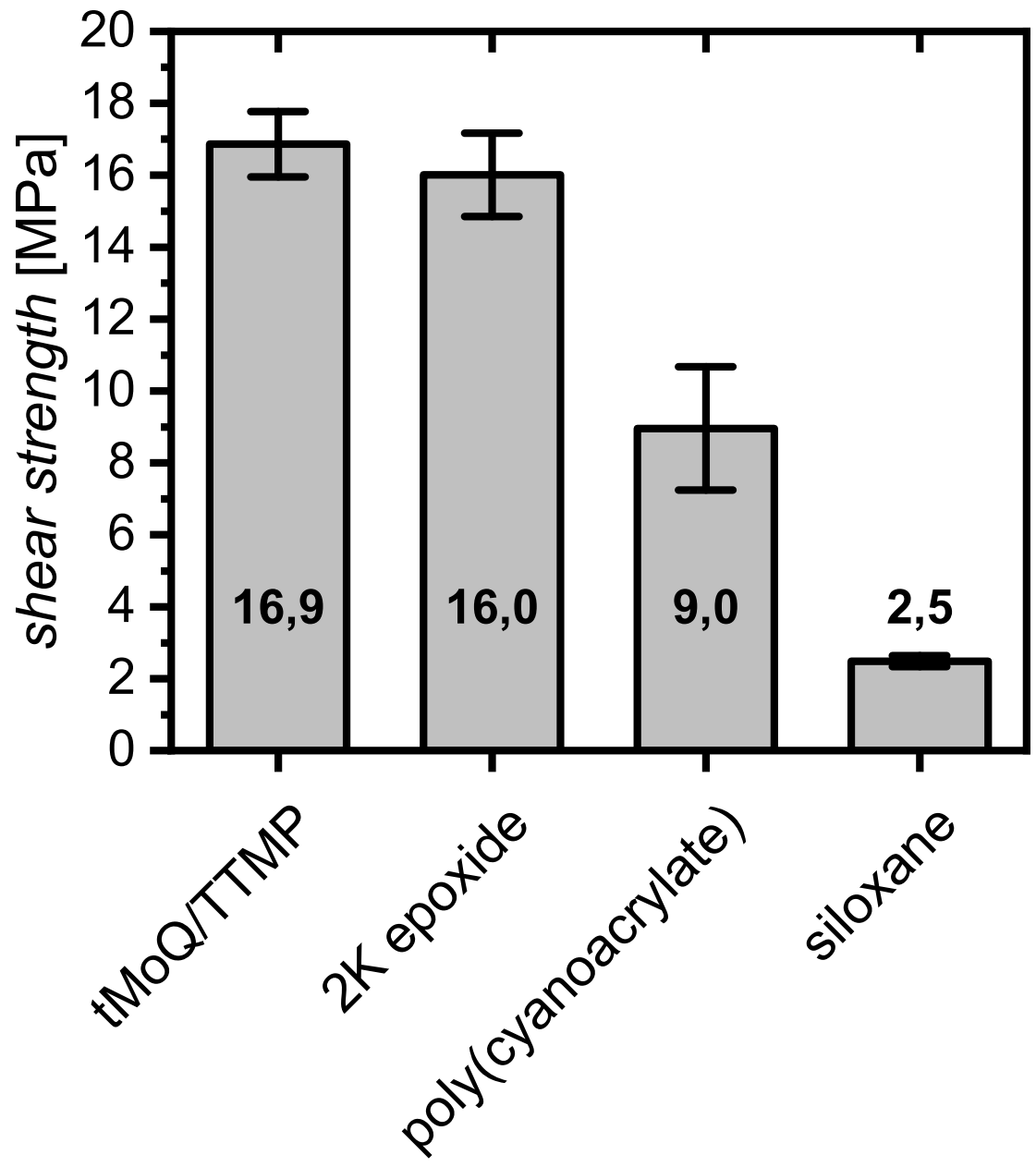

Figure S 40. Lap-shear strengths of aluminum joints bonded with tMoQ/TTMP (MoQ/T=1/1) cured at 90 °C compared to different commercial adhesives cured according to manufacturer instructions. A 2K epoxide ("UHU 2-K-Epoxidkleber", cured at RT for 24 h), a poly(cyanoacrylate) ("Pattex Sekundenkleber Mini Trio", cured at RT for 24 h) and a siloxane-based glue ("Pattex Repair Extreme", cured at RT for 24 h) were used. Error bars represent standard deviation, n=3.

To analyze the time dependency of curing, the tMoQ/PTMP formulation at MoQ/thiol ratio of 1/1 was used as a representative high-performing small-molecule adhesive to bond aluminum substrates and cured at 60 °C for different times (**Figure S 41**). Adhesive strength develops measurably after approximately 8 h and reached the maximum plateau strength after 12 h. Curing for 16 h gave comparable values with reduced scatter and was therefore retained as a robust full-cure condition for the structure–property study. The slight decrease in shear strength observed for 24 h curing might indicate excessive brittleness at elongated curing times limiting performance.

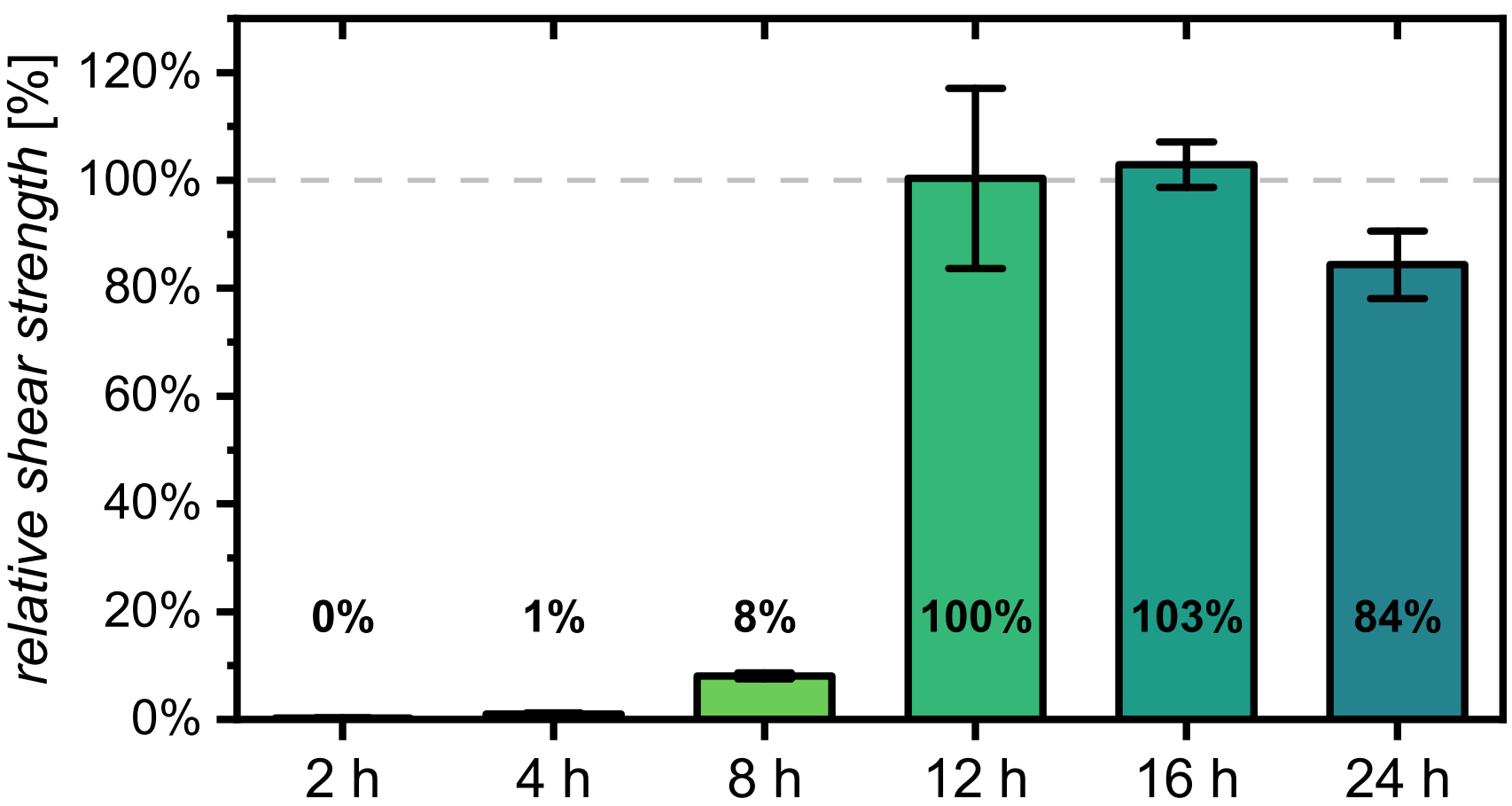

Figure S 41. Lap-shear strengths of aluminum joints bonded with tMoQ/PTMP (MoQ/T=1/1) cured at 60 °C for different times. Scaling of the lap-shear strengths was done with 100% being set to the 16.4 MPa value previously obtained for this adhesive formulation, shown in Figure 3b of the main manuscript. Error bars represent standard deviation, n=3.

Curing of the tMoQ based adhesives can also be carried out at lower temperatures and longer curing times, which might be more practical regarding application in industrial scenarios. For instance, the tMoQ/DHMP adhesive (MoQ/T = 1/1) shows a continuous rise in shear strength when curing at 30 °C for multiple days, until it even exceeds the reference shear strength for the standardized curing condition of 60 °C for 16 h after 6 days at 30 °C (**Figure S 42**). The final shear strength plateau is reached after 14 days at 30 °C, reaching a value of over 140% the performance compared to curing at 60 °C for 16 h. This could suggest brittleness for this highly crosslinked adhesive, even when curing at 60 °C, which can be prevented by curing at lower temperatures for longer periods of time.

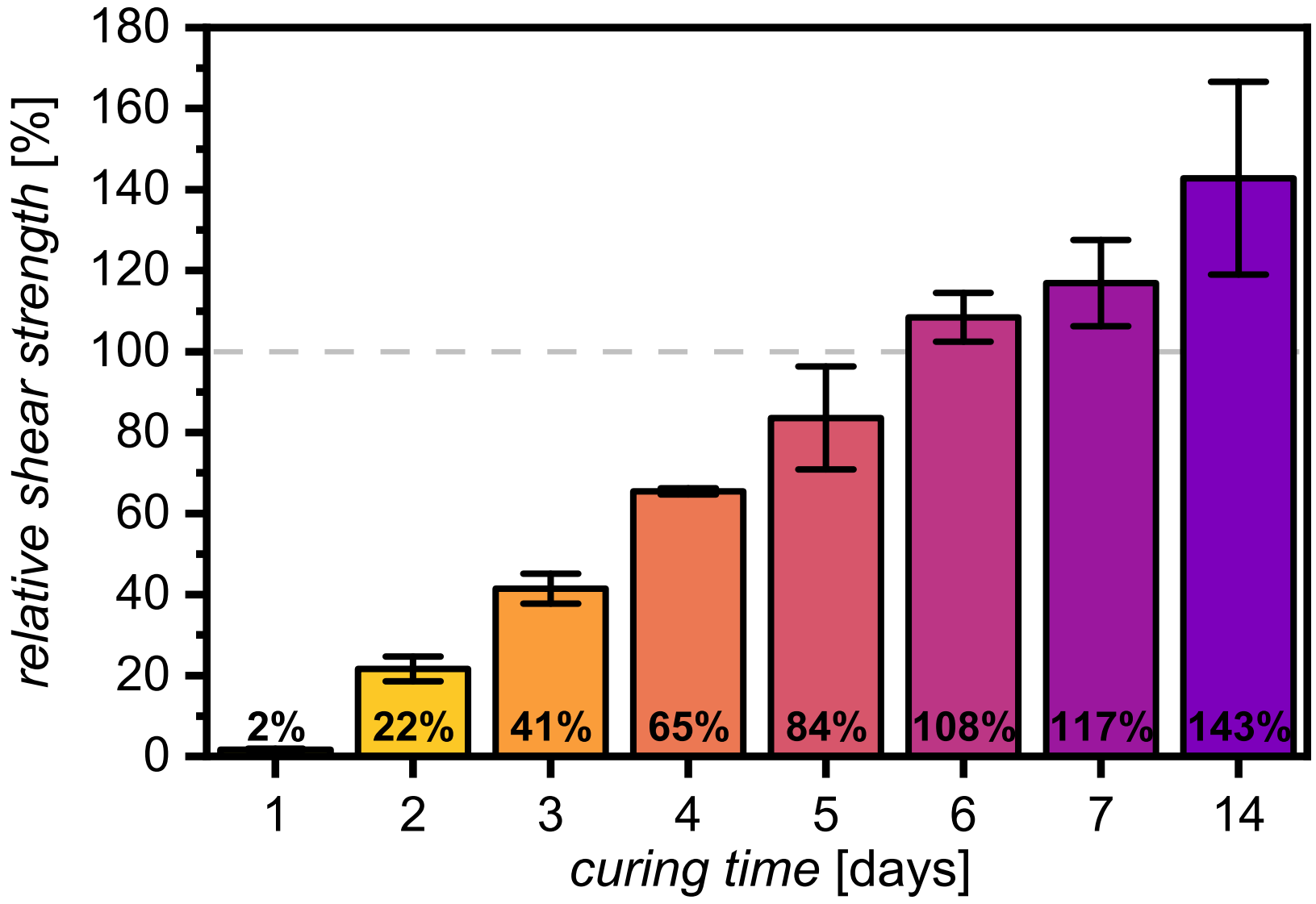

Figure S 42. Lap-shear strengths of aluminum joints bonded with tMoQ/DHMP (MoQ/T=1/1) cured at 30 °C for different times. Scaling of the lap-shear strengths was done with 100% being set to the 15.0 MPa value previously obtained for curing this adhesive formulation for 16 h at 60 °C, shown in Figure 3 of the main manuscript. Error bars represent standard deviation, n=3.

To assess substrate versatility, additional lap-shear experiments were performed, using the best-performing formulation of tMoQ/PTMP cured at 60 °C and evaluated the bonding performance on a broader set of substrates, including steel, various polymeric materials, and wood (**Figure S 43**). As expected, steel substrates showed adhesion performance comparable to aluminum. Polymeric substrates generally gave lower lap-shear strengths ranging from 3.0–4.5 MPa, which are still in the commodity adhesive regime. Interestingly, PS and PET showed the highest values of ~4.5MPa among the tested polymers. In contrast, PP exhibited poor adhesion, consistent with a low surface energy. For wood substrates, strong bonding was observed for both softwood and hardwood, with failures occurring within the substrate rather than at the adhesive bondlines. These additional experiments demonstrate that the TGC adhesive formulation is not limited to aluminum but can bond across different substrate classes, including metals, polymers, and wood.

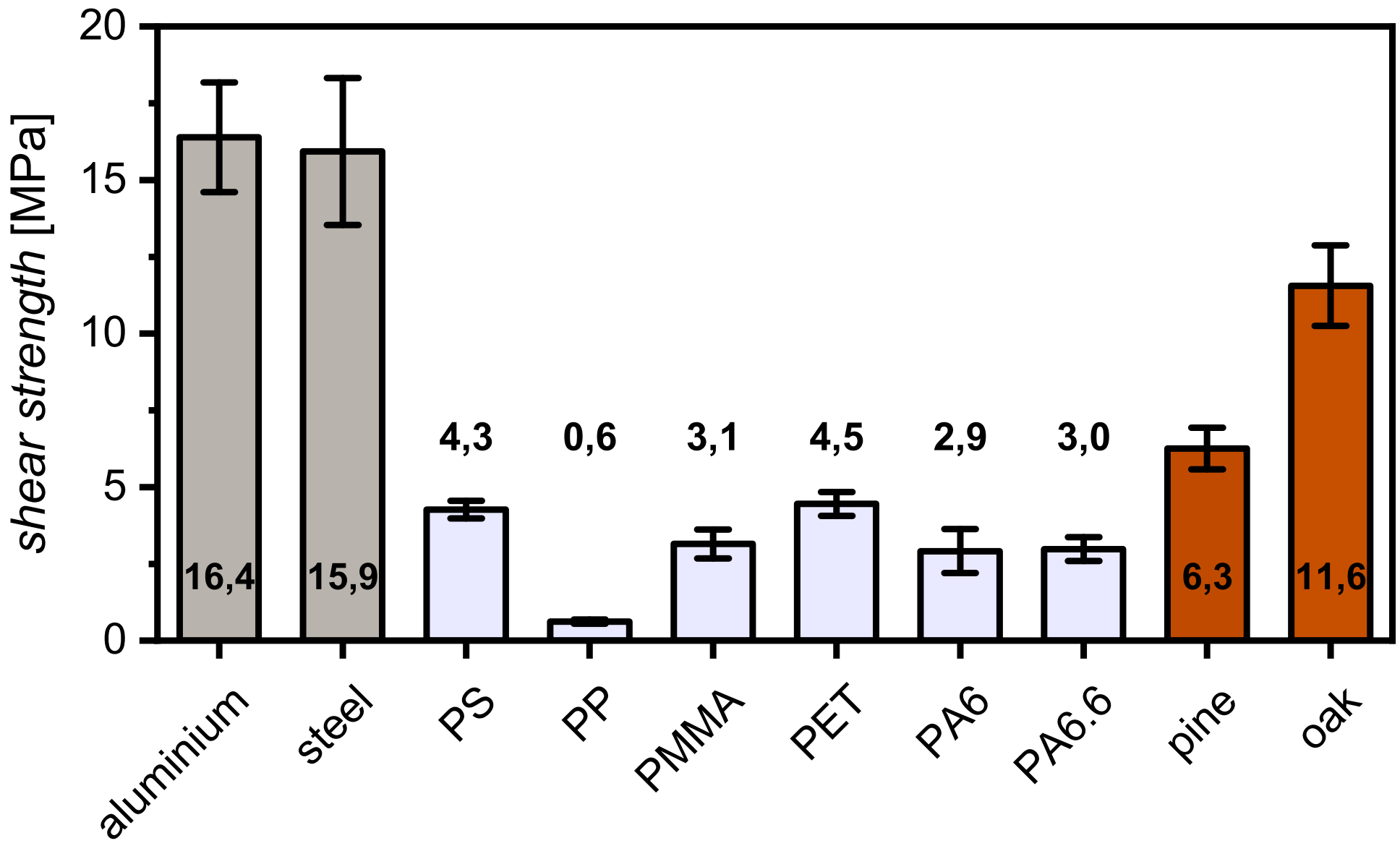

Figure S 43. Lap-shear strengths of different materials bonded with tMoQ/PTMP (MoQ/T=1/1) cured at 60 °C. Error bars represent standard deviation, n=3.

S5.14 Analysis of lignin

To quantify the functional groups present in the softwood lignin feedstock, quantitative ^{31}P NMR was carried out according to the following procedure:

Lignin (roughly 40 mg, accurately weighed) was dissolved in 0.5 mL of a pyridine/$CDCl_3$ mixture (1.6:1, v/v). To this solution, 100 µL of the internal standard solution (triphenylphosphine oxide, 0.01 mmol, 27.8 mg/mL in pyridine/$CDCl_3$, 1.6:1, v/v) and 50 µL of the relaxation agent solution (chromium(III) acetylacetonate, 11.4 mg/mL in pyridine/$CDCl_3$, 1.6:1, v/v) were added. Subsequently, 75 µL of 2-Chloro-4,4,5,5-tetramethyl-1,3,2-dioxaphospholane (TMDP) were added, the mixture was briefly shaken, and promptly transferred into an NMR tube for acquisition of the ^{31}P NMR spectrum according to the parameters in **Table S 15**.

The hydrolysis product of TMDP at 132.2 ppm was used as a reference, while the internal standard peak is visible at ~27 ppm. Integration was carried out between 150.0 ppm and 144.9 ppm for aliphatic OH-groups and between 144.9 ppm and 137.0 ppm for phenolic OH-groups. For the acetone soluble lignin this measurement was carried out in triplicate to obtain an exact functional group content as a baseline before PIDA oxidation. Values of 3.58 ±0.05 mmol/g phenolic OH and 2.06 ± 0.02 mmol/g aliphatic OH were obtained.

Table S 15. NMR parameters used for quantitative ^{31}P-NMR spectroscopy of lignin.

Pulse program	Inverse gated decoupling (zgig30)
Acquisition time (AQ)	0.8 s
Relaxation delay (D1)	10 s
Number of scans	128
Spectral width (SW)	250 ppm
Center of spectrum (O1P)	125 ppm

S5.15 PIDA oxidized lignin

When oxidizing softwood kraft lignin with PIDA the characteristic MoQ carbonyl band at roughly 1680 cm^{-1} becomes visible (**Figure S 44**), indicating formation of MoQ groups inside the lignin structure. Increasing the amount of PIDA results in a continuous increase of this carbonyl band alongside a decrease in OH vibrations, as a larger number of G-units are oxidized to MoQs.

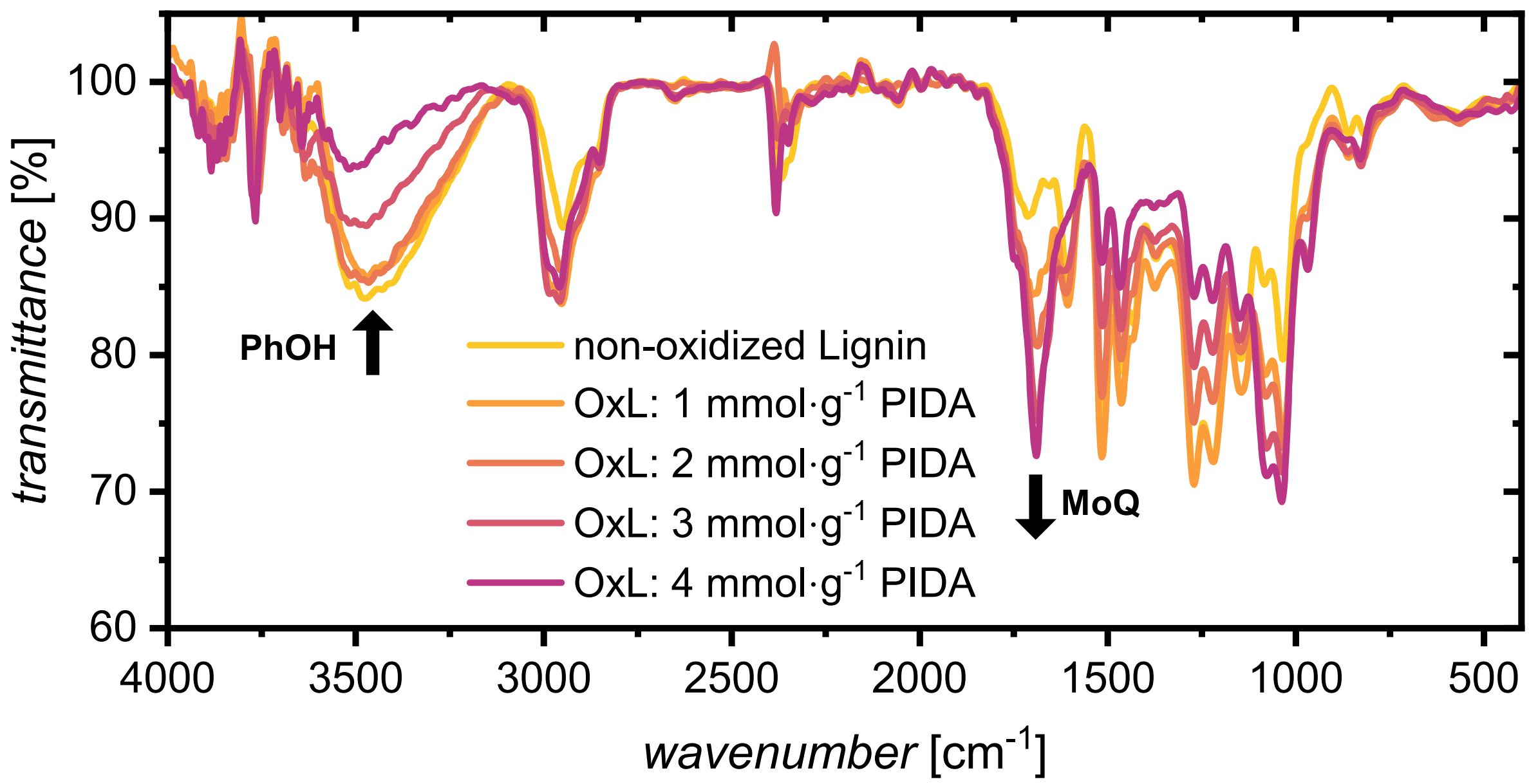

Figure S 44. FT-IR spectrum of non-oxidized as well as oxidized lignin utilizing different amounts of PIDA.

The amount of MoQ groups in the lignin was quantified by reaction with an excess of the monothiol 3-(trifluoromethyl)-benzyl mercaptan according to the following procedure:

Oxidized lignin (100 mg) was dissolved in 2.0 mL of NMP and 3-(trifluoromethyl)-benzyl mercaptan (153 µL, 1.0 mmol, 10 mmol/g) was added. After 24 h, the reaction mixture was precipitated in 50 mL diethyl ether and centrifuged. The residue was dissolved in 3 mL THF, precipitated twice more in 50 mL diethyl ether and once in 50 mL water. After freeze-dyring, the obtained TGC-lignin (roughly 20 mg, accurately weighed) was dissolved in 600 µL DMSO-d_6 and 50 µL of internal standard solution (pyridinium trifluoroacetate, 12.87 mg/mL in DMSO-d_6, 0.01 mmol fluorine) was added. A ^{19}F-NMR spectrum was recorded, where the pyridinium trifluoroacetate is observed at -74.0 ppm and the lignin bound thiol CF_3-groups at -60.0 to -61.5 ppm.

The one-to-one conversion of MoQ groups to TGC groups then allowed a quantification through the introduced CF_3-group, which is visible in the ^{19}F-NMR spectrum without any lignin background signal (**Figure S 45**).

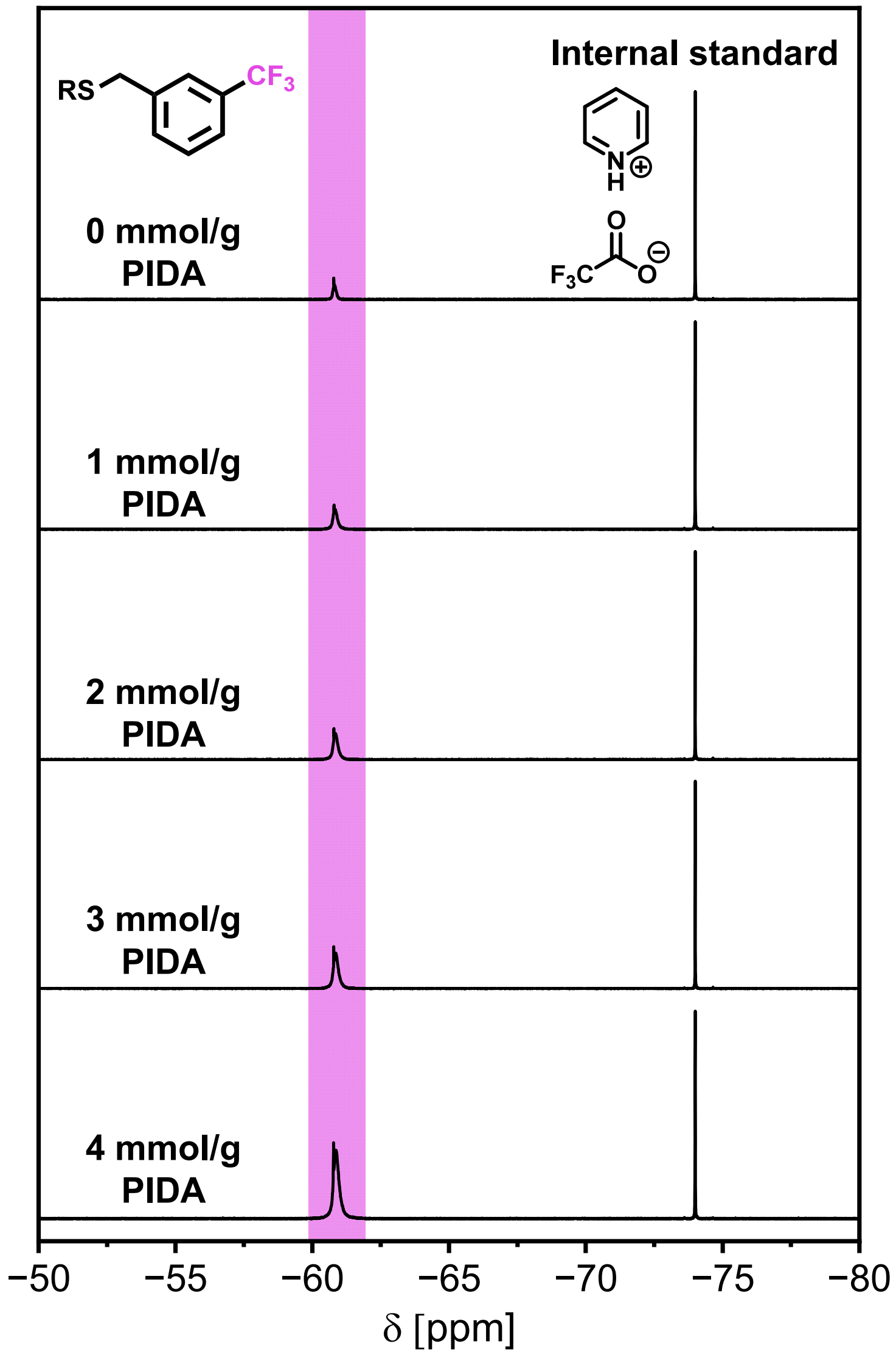

Figure S 45. ^{19}F NMR spectra of the different lignin samples (non-oxidized or oxidized with 1/2/3/4 mmol·g^{-1} PIDA) after reaction with an excess of 3-(trifluoromethyl)-benzyl mercaptan and precipitation in diethyl ether (three times), measured in DMSO-d_6.

This ^{19}F NMR method measures the TGC group content of the reaction product, which has to be converted to the reactive MoQ group content of the oxidized lignin. The change in molecular weight during TGC formation is known (loss of MeOH, addition of thiol), resulting in a gain of 160.2 g/mol per TGC group. For a known TGC content the underlying MoQ content can be calculated using the following formula:

$$MoQ\ content = \frac{\text{TGC content}}{1 - (\text{TGC content } \times 0.1602)}$$

The MoQ content of oxidized lignin is therefore always higher than the TGC content of that lignin after reaction with thiol, as the same number of functional groups are equal (one-to-one conversion of MoQ to TGC), while the total mass increases due to thiol addition, leading to an increased mass and thus decreased mmol/g value for the TGC lignin. All values for the oxidized lignins are summarized in **Table S 16**.

Table S 16. TGC and MoQ content measured via quantitative ^{19}F NMR of the different lignin samples (non-oxidized or oxidized with 1/2/3/4 mmol·g^{-1} PIDA) after reaction with an excess of 3-(trifluoromethyl)-benzyl mercaptan and precipitation in diethyl ether (three times).

	Amount of PIDA [mmol/g]	TGC content [mmol/g]	MoQ content [mmol/g]
**Lignin**	0	-	-
**OxL 1**	1	0.30	0.31
**OxL 2**	2	0.55	0.61
**OxL 3**	3	0.69	0.78
**OxL 4**	4	0.97	1.16

To show that this observed ^{19}F signal is caused by covalently bonded 3-(trifluoromethyl)-benzyl mercaptan and not just free thiol within the lignin matrix, ^{31}P NMR was measured according to the procedure in section **S5.14**. This allows for the quantification of free thiols, as they give a distinct signal in the 210-220 ppm range after phosphitylation.[19] To show that this method works to quantify free thiols in a lignin containing solution, a sample of non-oxidized lignin was phosphitylated and 0.69 µL 3-(trifluoromethyl)-benzyl mercaptan were added. The resulting ^{31}P NMR spectrum (**Figure S 46**) shows signals for both the lignin OH-groups (3.61 mmol/g phenolic OH and 2.08 mmol/g aliphatic OH) and thiol SH-groups (95% of theoretical amount). This method therefore allows for the distinction of free thiols vs thiols covalently bound to the lignin, with the results summarized in **Table S 17**. In general, a minimal amount of free thiol of less than 0.02 mmol/g is detected, which corresponds to at most 3% of TGC groups. Therefore, the detected ^{19}F-NMR signal can be attributed to covalent TGC adducts of the fluorinated thiol on the lignin structure, as free thiol accounts for less than 3%.

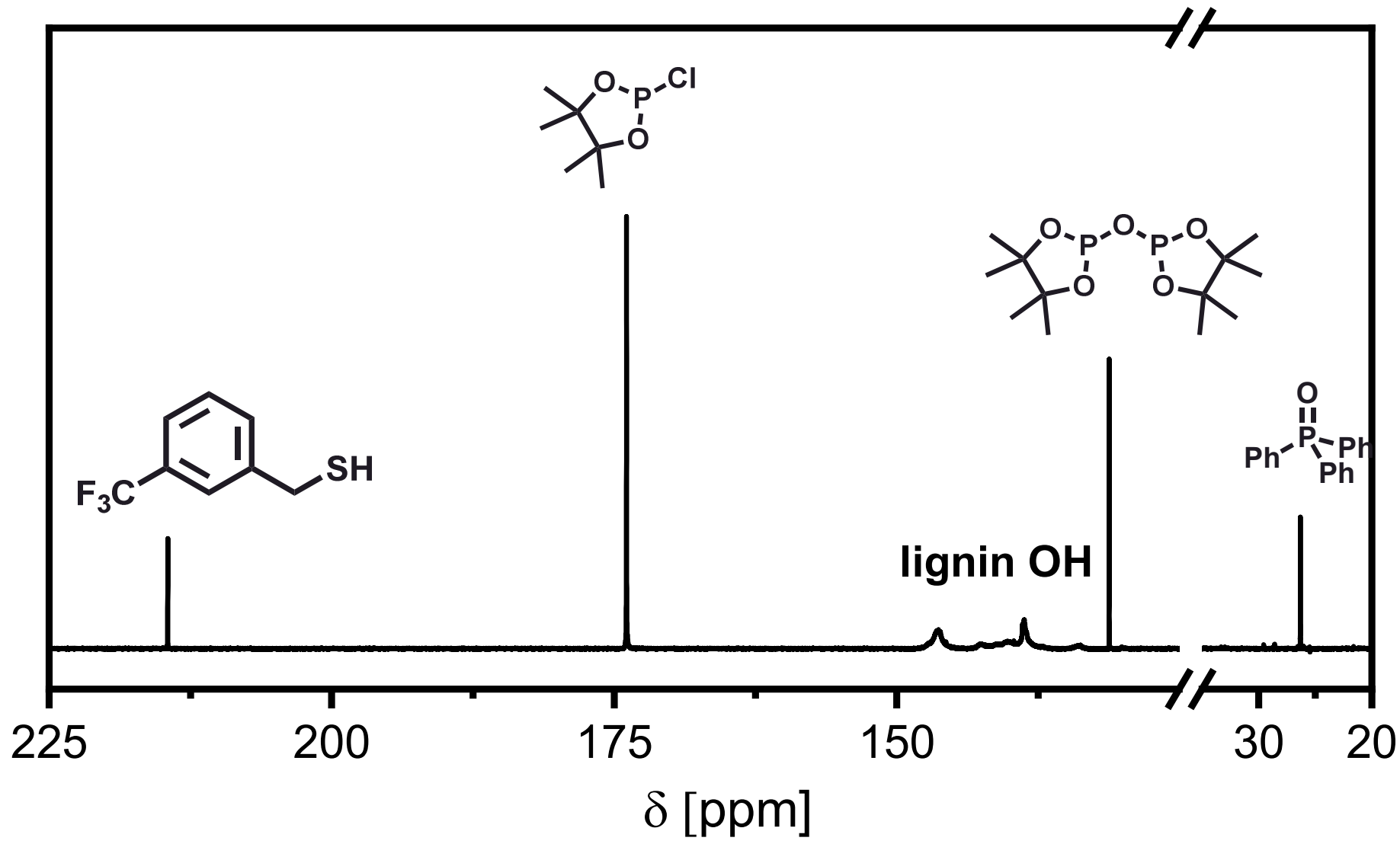

Figure S 46. ^{31}P NMR spectrum of non-oxidized lignin in pyridine/$CDCl_3$ (1.6/1) after phosphitylation with TMPD and addition of 0.15 mmol/g 3-(trifluoromethyl)-benzyl mercaptan with triphenylphosphine as internal standard.

Table S 17. Characterization of the oxidized lignin derivatives. Functional group analysis of the different lignin samples (oxidized with different PIDA equivalents and reacted with 3-(trifluoromethyl)-benzyl mercaptan to TGC-lignin). ^{31}P NMR was carried out for the TGC-lignins due to poor solubility of oxidized lignins in the necessary pyridine/$CDCl_3$ mixtures. ^{1}H NMR was performed for the oxidized MoQ- lignins, which were readily soluble in DMSO except for OxL 4.

	Amount of PIDA [mmol/g]	Phenolic OH [mmol/g][a]	Aliphatic OH [mmol/g][a]	Methoxy [mmol/g][b]	MoQ [mmol/g][c]	TGC [mmol/g][c]	Free thiol [mmol/g][a]
**Lignin**	0	3.61	2.08	8.64	-	-	0.00 (0%)
**OxL 1**	1	3.08	2.03	8.93	0.31	0.30	0.01 (3%)
**OxL 2**	2	2.28	1.83	10.77	0.61	0.55	0.01 (2%)
**OxL 3**	3	1.70	1.74	11.26	0.78	0.69	0.01 (1%)
**OxL 4**	4	1.41	1.69	-	1.16	0.97	0.02 (2%)

[a)] quantitative ^{31}P NMR, [b)] ^{1}H-NMR and [c)] quantitative ^{19}F NMR. MoQ-group content is calculated based on TGC content.

A clear trend of decreasing phenolic OH-content for the TGC lignins with increasing degree of oxidation is observed. This could be caused by the presence condensed guaiacols or other phenolic units in the lignin structure. These groups can still be oxidized by PIDA, but probably don't react efficiently with thiols and thus don't regenerate their phenolic OH-groups, unlike MoQ-groups being converted to TGC-groups. Alternatively, fractions of the MoQ sites in the activated lignin could remained unreacted, probably due to accessibility reasons. This is consistent with the persistence of MoQ carbonyl vibration bands in IR spectra of TGC-lignins after thiol reaction (**Figure S 47**) and agrees well with the incomplete MoQ-to-TGC conversions observed for dMoQ or tMoQ monomers. Aliphatic OH-content also shows a continuous decline. Since PIDA is not capable of oxidizing aliphatic alcohols under the employed conditions, this decrease is likely caused by additional thiol residues introduced through TGC-linkages. For the highest value of 0.97 mmol/g TGC-groups and a thiol molecular weight of 192.2 g/mol roughly 19 wt% of this lignin sample is made up of the covalently added thiol. Therefore, the initially present 2.08 mmol/g aliphatic OH groups for the non-oxidized lignin are also reduced by 19%, as aliphatic alcohols are only present in the lignin but not the TGC-groups. The measured value of 1.69 mmol/g is in good agreement with the expected value of 1.68 mmol/g for this calculation, as is the case for all other samples.

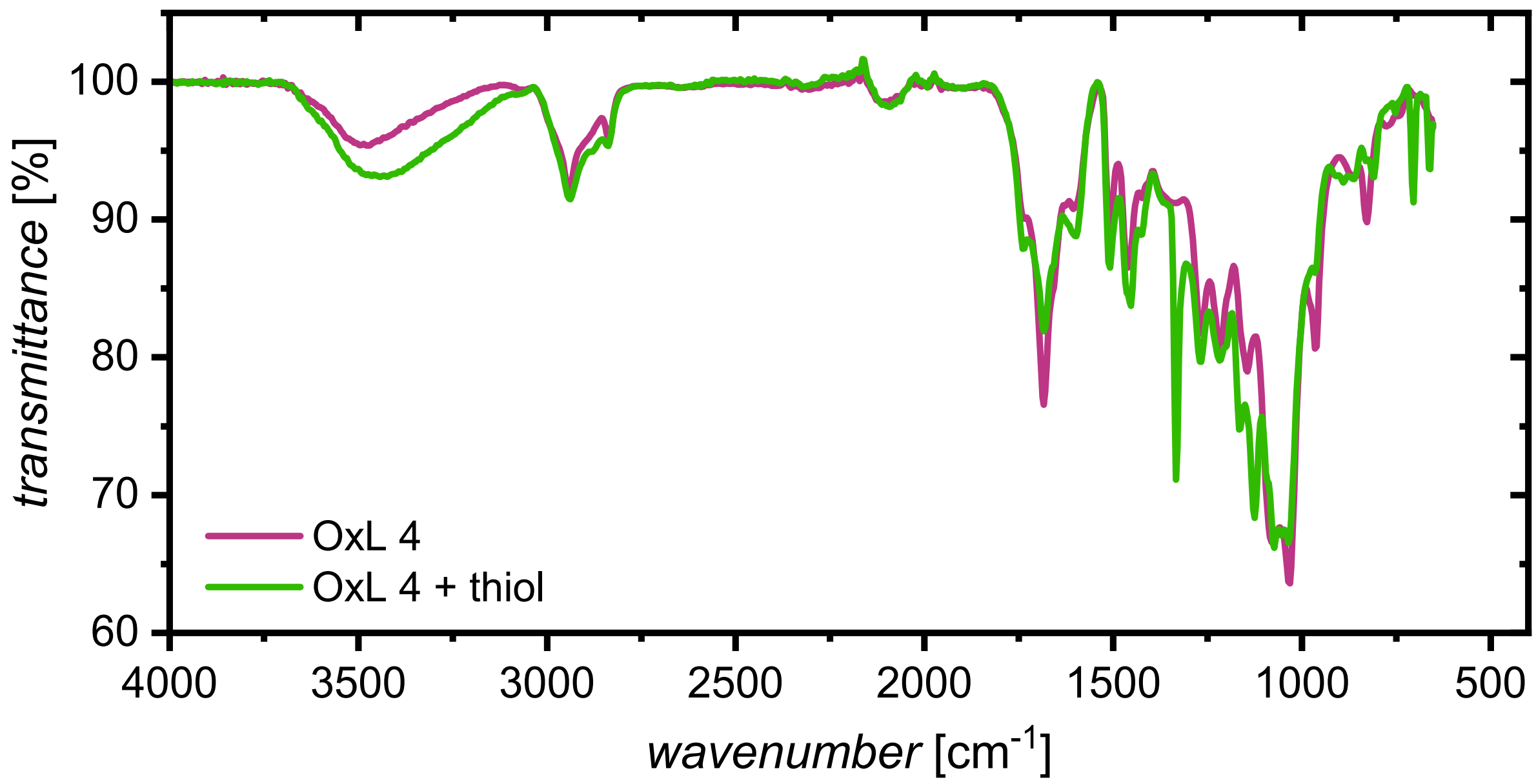

Figure S 47. FT-IR spectrum of OxL 4 before and after reaction with the thiol 3-(trifluoromethyl)-benzyl mercaptan.

GPC analysis revealed a mostly constant number average molar mass M_n of around 5000 g/mol and a strong increase of mass average molar mass M_w from 9900 g/mol up to 39000 g/mol with increasing amounts of oxidant (**Figure S 48**). This is caused by an increasingly bimodal distribution with a high molecular weight shoulder. A likely cause for this is the DA dimerization of the MoQ-groups, as shown by the characteristic IR carbonyl band for the highly oxidized lignin samples at 1737 cm^{-1} (**Figure S 47**). Additionally, reacting the oxidized lignin with a monothiol directly in solution before isolation greatly reduced the magnitude of the high molecular weight shoulder (**Figure S 49**). This further confirms the DA dimerization during precipitation of the oxidized lignins as the main reason for this observed partial crosslinking.

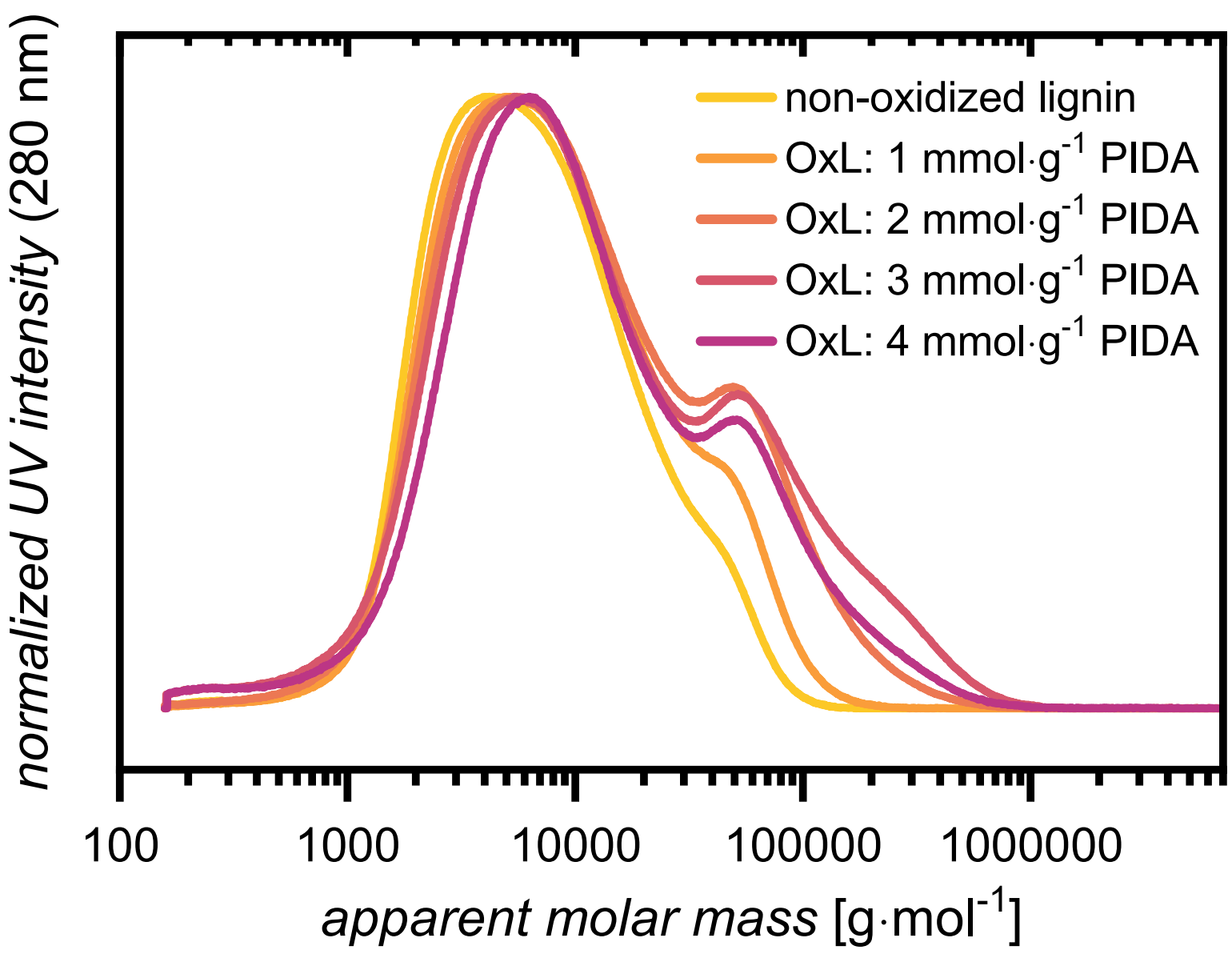

Figure S 48. GPC analysis of the different lignin samples (non-oxidized or oxidized with 1/2/3/4 mmol·g^{-1} PIDA and reacted with 3-(trifluoromethyl)-benzyl mercaptan to form TGC-lignin). Conditions: DMSO containing 0.075 M $NaNO_3$, flow rate of 0.5 mL/min and sample concentration of 2 mg/mL.

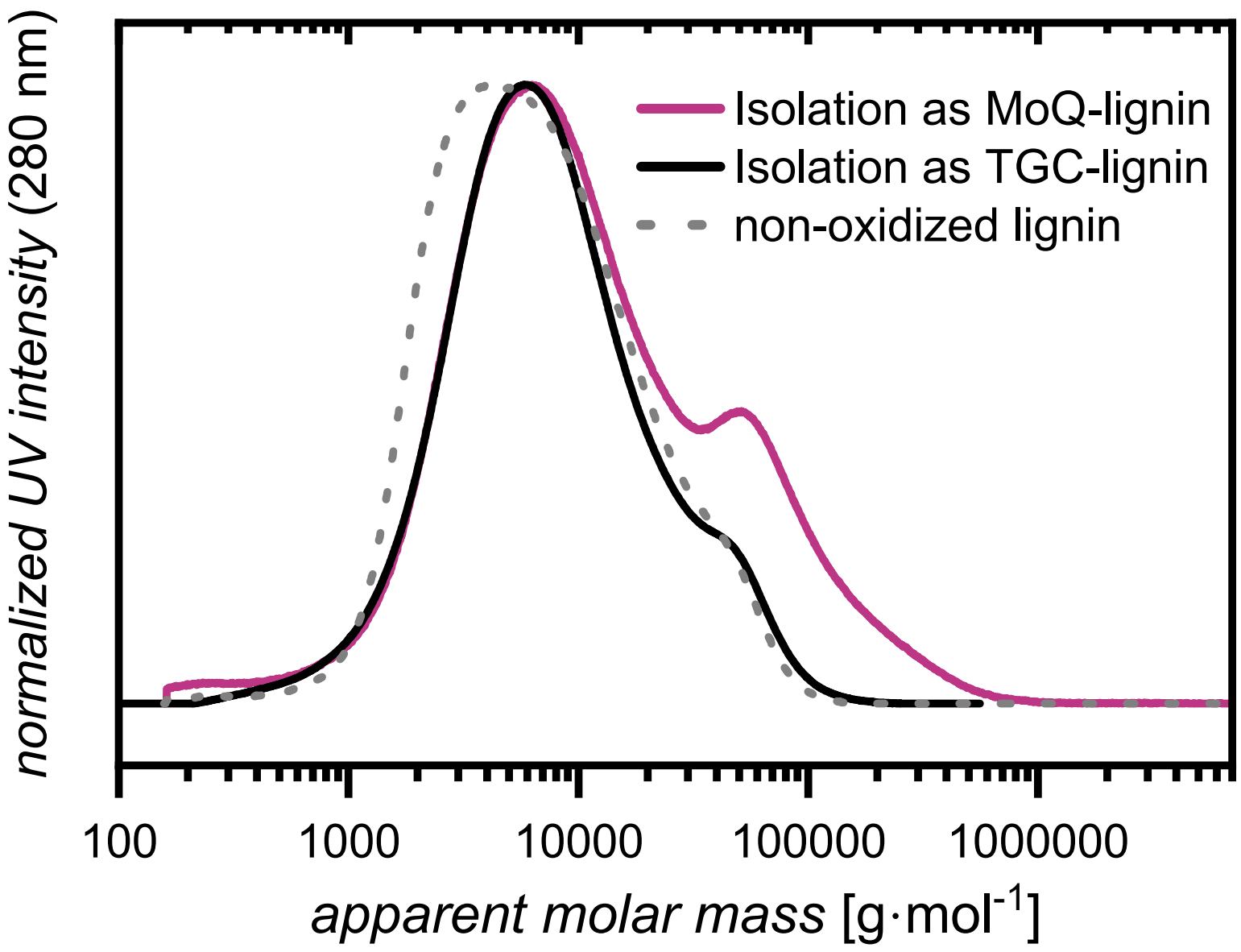

Figure S 49. GPC analysis of the oxidized lignin (4 mmol·g^{-1} PIDA) reacted with 3-(trifluoromethyl)-benzyl mercaptan to form TGC-lignin. Isolation either took place as MoQ-lignin or the isolation took place as TGC-lignin when adding the thiol in situ directly after oxidation. Conditions: DMSO containing 0.075 M $NaNO_3$, flow rate of 0.5 mL/min and sample concentration of 2 mg/mL.

Table S 18. DMSO-GPC analysis of the different lignin samples (non-oxidized or oxidized with 1/2/3/4 mmol·g^{-1} PIDA and reacted with 3-(trifluoromethyl)-benzyl mercaptan to form TGC-lignin) and calculation of TGC groups per lignin molecule based on quantitative ^{19}F NMR measurement of TGC groups.

	Amount of PIDA [mmol/g]	M_n [g/mol]	M_w [g/mol]	TGC [mmol/g]	TGC-groups per molecule based on M_n	TGC-groups per molecule based on M_w
**Lignin**	0	4300	9900	-	-	-
**OxL 1**	1	4800	12000	0.30	1.4	3.6
**OxL 2**	2	5600	24000	0.55	3.1	13
**OxL 3**	3	4800	39000	0.69	3.3	27
**OxL 4**	4	4900	30000	0.97	4.8	29
**OxL 4 (in situ)***	4	4400	12000	1.00	4.4	12

*obtained by directly quenching the oxidized lignin with 3-(trifluoromethyl)-benzyl mercaptan before precipitation

S5.16 Lignin based 2K mixtures

The viscosity of the different lignin-based 2K mixtures was measured using a rotational viscosity test at 20 °C (**Table S 19**). The Viscosity of the OxL 4 and $ETTMP_{1300}$ mixture (50/50 wt%) could not be measured, as the maximal torque of the device (230 mN·m) was reached before a shear rate of 0.01 s^{-1} was achieved. Additionally, the dynamic modulus of the mixtures was measured at 0 °C and 20 °C to better characterize these highly viscous samples (**Table S 20**).

Table S 19. Rotational viscosity of the different 2K adhesives consisting of lignin (non-oxidized or oxidized with 1/2/3/4 mmol·g^{-1} PIDA) and $ETTMP_{1300}$ (50/50 wt%) at 20 °C.

	**Amount of PIDA [mmol/g]**	**Viscosity [mPa·s] at 0.01 s^{-1} shear rate**	**Viscosity [mPa·s] at 0.1 s^{-1} shear rate**	**Viscosity [mPa·s] at 1 s^{-1} shear rate**
**Lignin**	0	$2.3 \cdot 10^6$	$2.0 \cdot 10^7$	$1.1 \cdot 10^7$
**OxL 1**	1	$4.8 \cdot 10^6$	$6.5 \cdot 10^7$	$2.2 \cdot 10^7$
**OxL 2**	2	$2.7 \cdot 10^7$	$8.6 \cdot 10^7$	$2.4 \cdot 10^7$
**OxL 3**	3	$5.2 \cdot 10^8$	$3.3 \cdot 10^8$	$5.0 \cdot 10^7$
**OxL 4**	4	not measurable	not measurable	not measurable

Table S 20. Dynamic modulus of the different 2K adhesives consisting of lignin (non-oxidized or oxidized with 1/2/3/4 mmol·g^{-1} PIDA) and $ETTMP_{1300}$ (50/50 wt%) at different temperatures measured via oscillatory rheological analysis (γ = 0.1%, ω = 10 rad·s^{-1}).

	**Amount of PIDA [mmol/g]**	**Dynamic modulus [Pa] at 0 °C**	**Dynamic modulus [Pa] at 20 °C**
**Lignin**	0	$6.0 \cdot 10^5$	$1.7 \cdot 10^4$
**OxL 1**	1	$5.6 \cdot 10^5$	$2.2 \cdot 10^4$
**OxL 2**	2	$5.9 \cdot 10^5$	$2.9 \cdot 10^4$
**OxL 3**	3	$7.3 \cdot 10^5$	$6.6 \cdot 10^4$
**OxL 4**	4	$2.8 \cdot 10^6$	$5.5 \cdot 10^5$

The curing behavior of these lignin based 2K mixtures was investigated by oscillatory rheological analysis across a temperature range of 0-120 °C (**Figure S 50**) as well as differential scanning calorimetry (DSC, **Figure S 52** and **Figure S 52**). The rheological analysis showed earlier sol-gel-transitions for oxidized lignins with higher MoQ-contents: While the non-oxidized lignin did not show a transition and remained liquid over the entire measurement, the sol-gel temperature T_{sg} decreased from 85 °C to 67 °C to 37 °C, when oxidizing with 1, 2 or 3 mmol/g PIDA, respectively. On the other hand, the lignin oxidized with 4 mmol/g PIDA showed no sol-gel transition, as it remained as a gel over the entire measurement range.

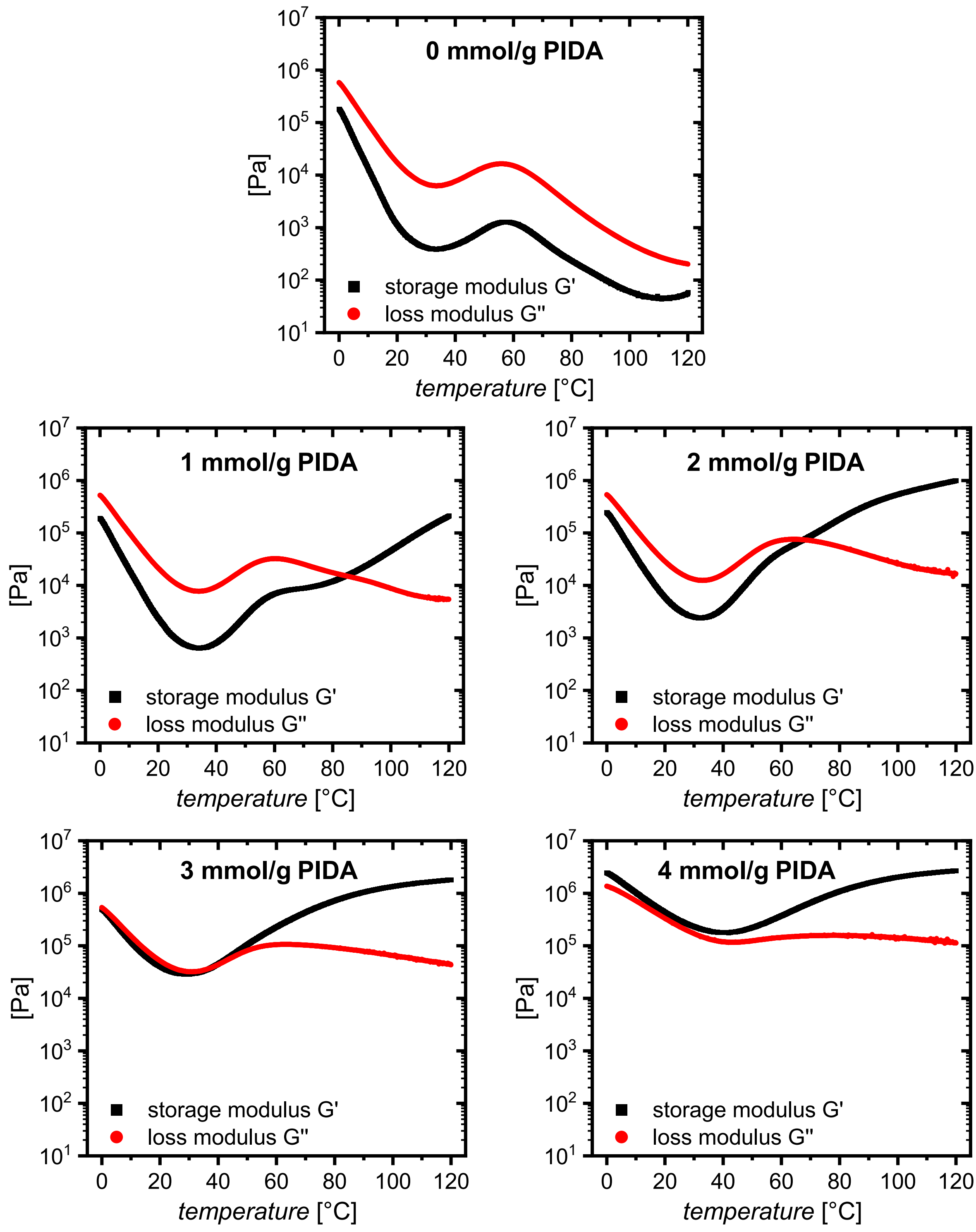

Figure S 50. Rheological analysis (γ = 0.1%, ω = 10 rad·s^{-1}) of the 2K mixtures consisting of lignin (non-oxidized or oxidized with 1/2/3/4 mmol·g^{-1} PIDA) and $ETTMP_{1300}$ (50/50 wt%) with a temperature range of 0 °C to 120 °C (1 °C·min^{-1}).

The DSC measurements showed similar onsets for all samples, ranging from 35 °C to 40 °C (**Figure S 51**). Interestingly, a bimodal exothermic peak at 65 °C and 95 °C is observed for all oxidized lignins. This first peak seems to originate from the naturally present electrophilic groups in the lignin, as it is already present in the non-oxidized lignin. Increasing MoQ-contents lead to increased intensity of the exothermic peak at 95 °C, which is in agreement with the DSC measurements for tMoQ based mixtures.

Specific heat release for the lignin 2K mixtures follows a linear trend up to a degree of oxidation of 3 mmol/g PIDA, where a plateau and a maximum value of 83 J/g is reached (**Figure S 52**). This indicates incomplete curing for the mixture utilizing lignin oxidized with 4 mmol/g PIDA, likely caused by the high viscosity and degree of crosslinking for that system.

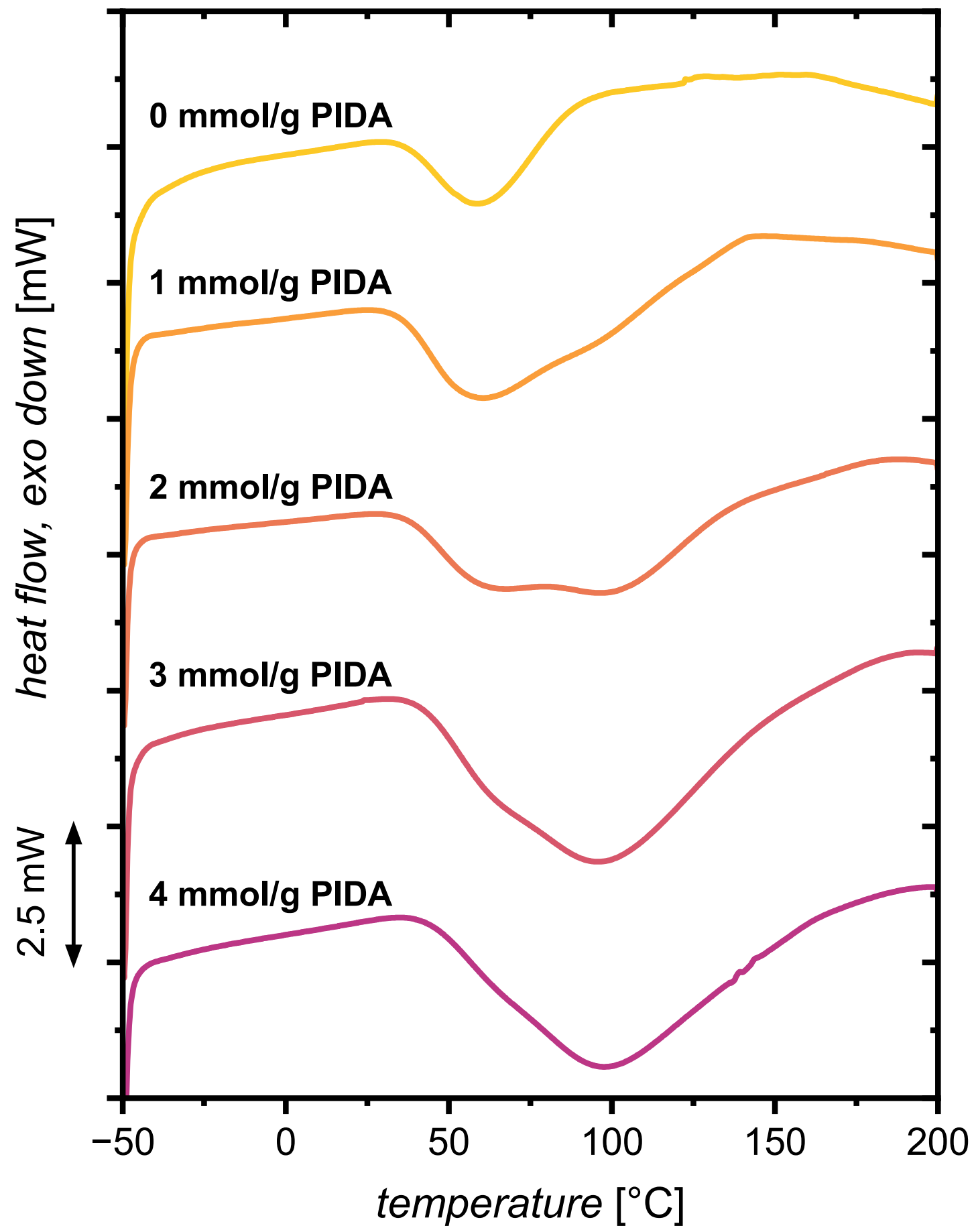

Figure S 51. Differential scanning calorimetry analysis of the curing of the 2K mixtures consisting of lignin (non-oxidized or oxidized with 1/2/3/4 mmol·g^{-1} PIDA) and ETTMP$_{1300}$ (50/50 wt%) with a heating rate of 10 °C/min under argon.

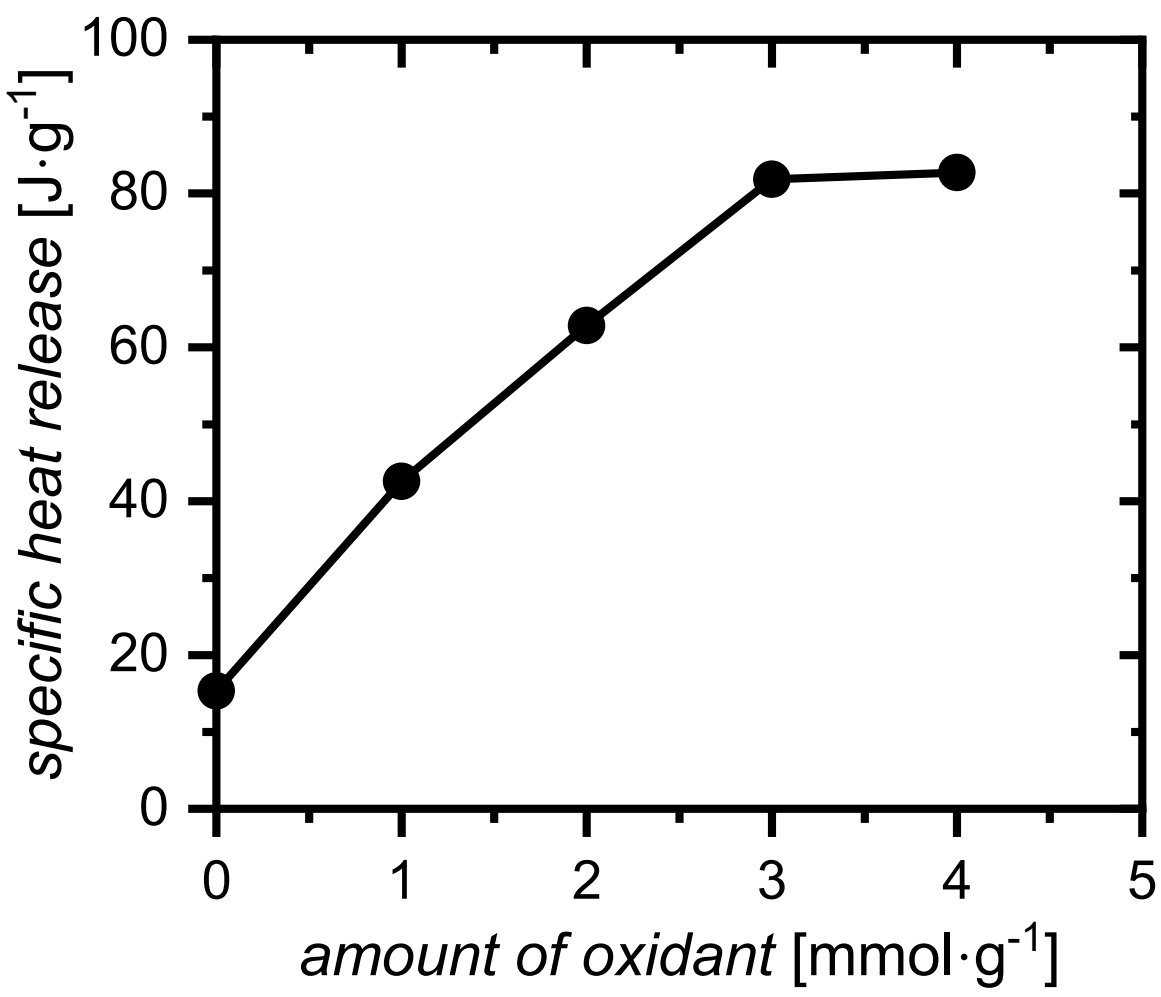

Figure S 52. Specific heat release upon curing of the 2K mixtures consisting of lignin (non-oxidized or oxidized with 1/2/3/4 mmol·g^{-1} PIDA) and ETTMP$_{1300}$ (50/50 wt%) obtained by integration of the heat flow singal.

S5.17 Lignin based 2K adhesives

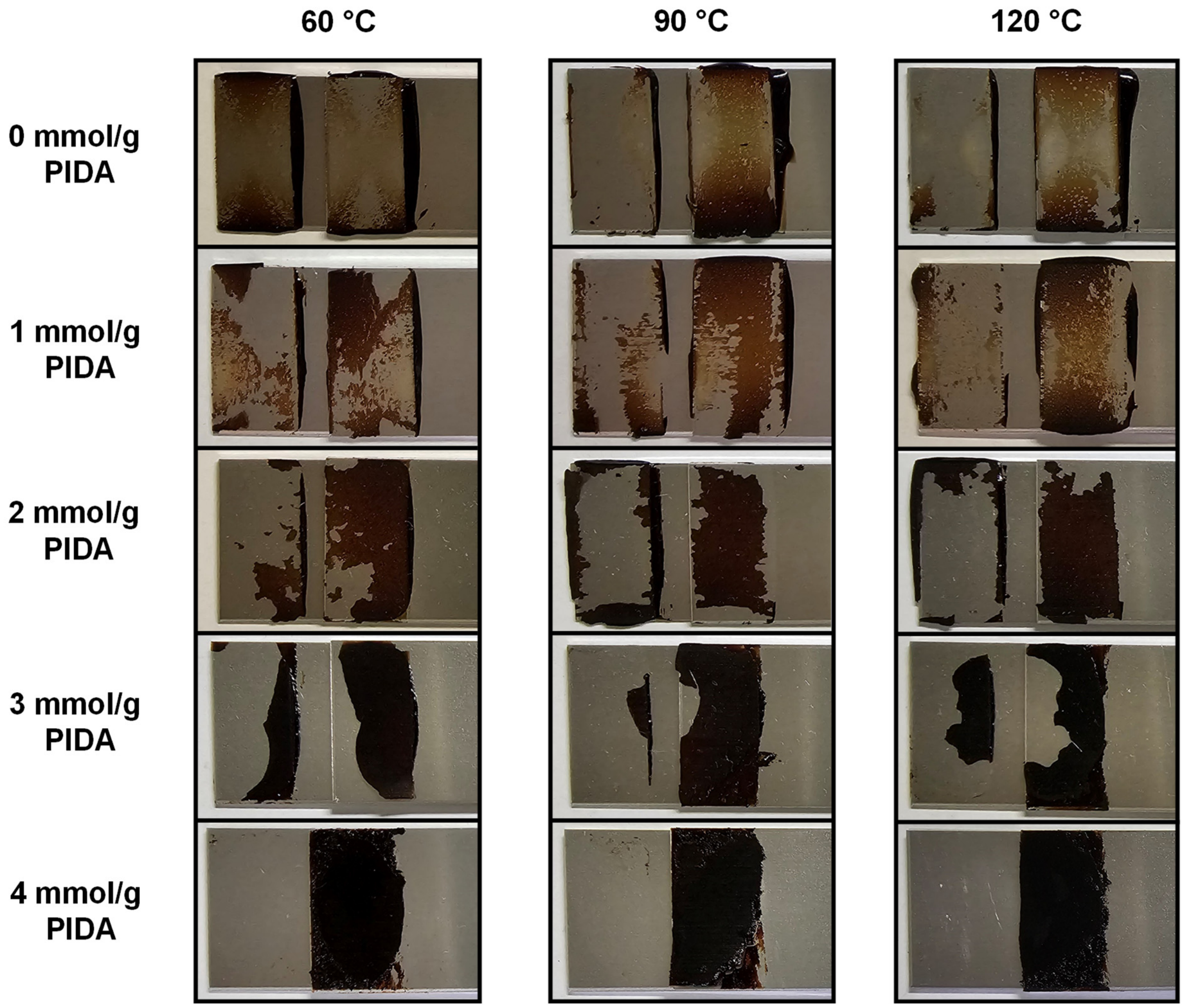

Figure S 53. Fracture patterns of the different 2K adhesives consisting of lignin (non-oxidized or oxidized with 1/2/3/4 mmol·g^{-1} PIDA) and ETTMP$_{1300}$ (50/50 wt%), cured for 16 h at 60 °C, 90 °C or 120 °C.

Table S 21. Glass transition temperatures and fracture patterns of 2K adhesives consisting of lignin (non-oxidized or oxidized with 1/2/3/4 mmol·g^{-1} PIDA) and $ETTMP_{1300}$ (50/50 wt%). Formulations were cured for 16 h at 60, 90, or 120 °C. Glass transition temperatures were determined by DSC at a heating rate of 10 °C min^{-1}.

Amount of PIDA	Curing temperature (for 16 h)	Glass transition temperature T_g	Fracture pattern
0 mmol/g	60 °C	-16 °C	cohesive
	90 °C	-10 °C	mixed
	120 °C	10 °C	mixed
1 mmol/g	60 °C	-2 °C	mixed
	90 °C	8 °C	mixed
	120 °C	20°C	mixed
2 mmol/g	60 °C	1 °C	adhesive
	90 °C	10 °C	adhesive
	120 °C	16 °C	adhesive
3 mmol/g	60 °C	-3 °C	adhesive
	90 °C	13 °C	adhesive
	120 °C	11 °C	adhesive
4 mmol/g	60 °C	-13 °C	adhesive
	90 °C	12 °C	adhesive
	120 °C	8 °C	adhesive

Table S 22. Statistical analysis of lap-shear strengths for 2K adhesives consisting of lignin (non-oxidized or oxidized with 1/2/3/4 mmol·g^{-1} PIDA) and $ETTMP_{1300}$ (50/50 wt%), comparing different formulations cured at the same temperature. Significance levels are indicated as follows: $p \geq 0.05$ not significant (n.s.), $p < 0.05$ (*), $p < 0.01$ (**), $p < 0.001$ (***).

comparison	curing temperature		
	60 °C	90 °C	120 °C
Lignin - OxL 1	**	**	**
Lignin - OxL 2	**	*	n.s.
Lignin - OxL 3	*	n.s.	n.s.
Lignin - OxL 4	n.s.	n.s.	*
OxL 1 - OxL 2	n.s.	n.s.	n.s.
OxL 1 - OxL 3	*	**	*
OxL 1 - OxL 4	n.s.	**	**
OxL 2 - OxL 3	*	*	n.s.
OxL 2 - OxL 4	n.s.	**	*
OxL 3 - OxL 4	n.s.	*	n.s.

Table S 23. Statistical analysis of lap-shear strengths for 2K adhesives consisting of lignin (non-oxidized or oxidized with 1/2/3/4 mmol·g^{-1} PIDA) and ETTMP$_{1300}$ (50/50 wt%), comparing the same formulation cured at different temperatures. Significance levels are indicated as follows: $p \geq 0.05$ not significant (n.s.), $p < 0.05$ (*), $p < 0.01$ (**), $p < 0.001$ (***).

	curing temperature		
	**60 °C vs 90 °C**	**60 °C vs 120 °C**	**90 °C vs 120 °C**
**Lignin**	**	***	*
**OxL 1**	***	**	n.s.
**OxL 2**	*	n.s.	n.s.
**OxL 3**	n.s.	n.s.	n.s.
**OxL 4**	n.s.	n.s.	n.s.

To analyze the time dependency of curing, the lignin oxidized with 1 mmol·g^{-1} PIDA in combination with ETTMP$_{1300}$ (50/50 wt%) was used as a representative high-performing adhesive to bond aluminum substrates and cured at 120 °C for different times (**Figure S 54**). The adhesive already reached 67% of the final adhesive strength after 2 h and 95% after 8 h when comparing to a curing time of 16 h. This faster strength build-up is consistent with the multifunctional character of oxidized lignin and the macromolecular trithiol, where a small amount of TGC formation can already generate an effective network. After the initial network build-up, further curing proceeds more slowly likely due to reduced mobility and increased steric constrains within the developing resin. Therefore, good lap-shear strengths are already reached after 8 h and even slightly better values can be obtained when curing for 24 h, the curing time of 16 h, chosen for both tMoQ and lignin-based formulations, represents a standardized full-cure condition for comparative evaluation, not the shortest technically accessible curing time.

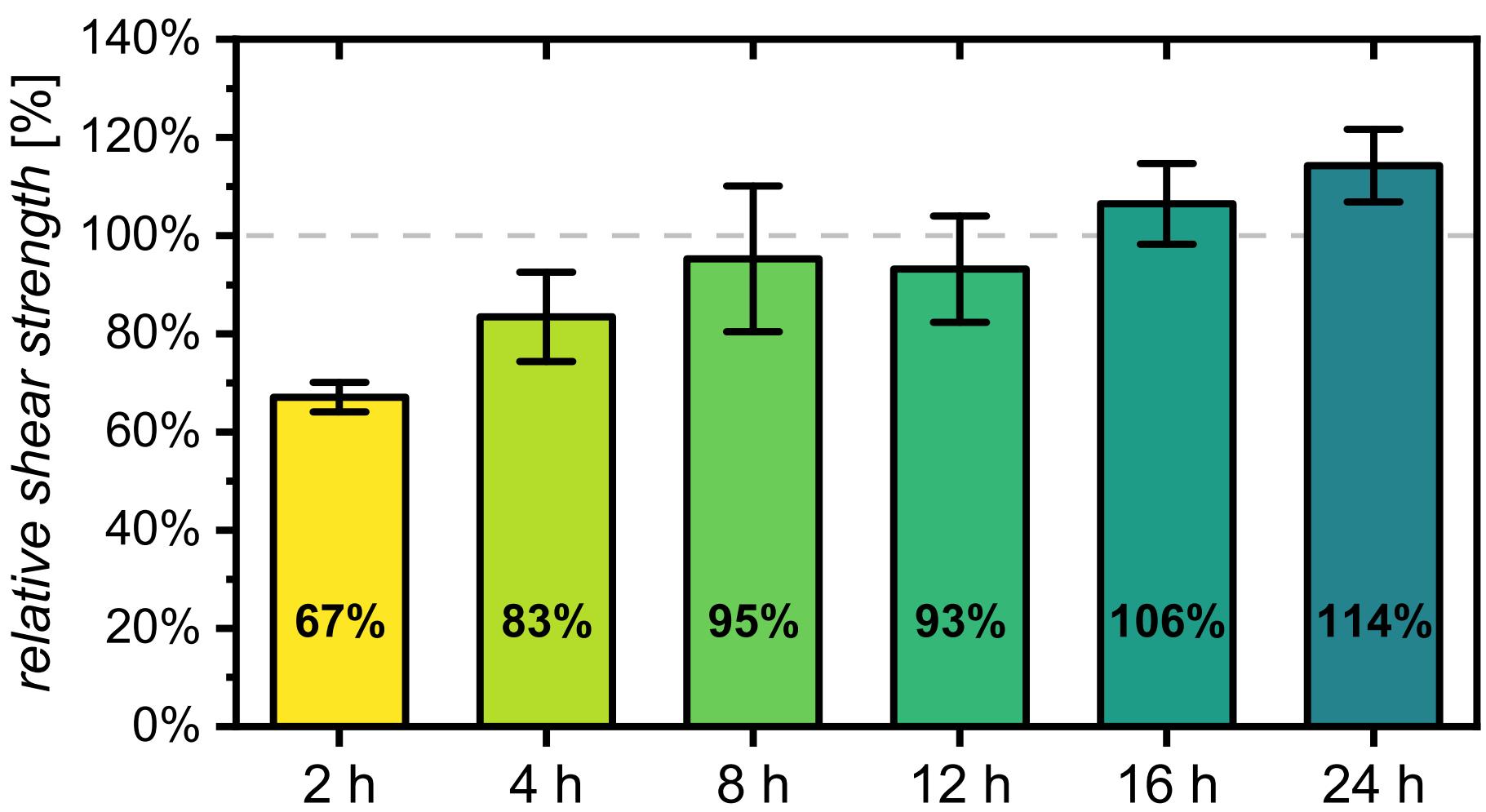

Figure S 54. Lap-shear strengths of aluminum joints bonded with lignin TGC adhesive (1 mmol/g PIDA, lignin/ETTMP1300 50/50 wt%) cured at 120 °C for different times. Scaling of the lap-shear strengths was done with 100% being set to the 9.1 MPa value previously obtained for this adhesive formulation, shown in Figure 4c of the main manuscript. Error bars represent standard deviation, n=3.

To assess the moisture resistance of the lignin-based adhesives the best performing activated lignin oxidized with 1 mmol/g PIDA was chosen as a representative sample. A focused moisture durability screen on the lignin adhesive using aluminum substrates, benchmarked against reference curing and conditioning at 25% relative humidity (RH) standard laboratory environment was performed. The bonded specimens were stored at 25 °C and 100% RH for 24 h, then tested immediately after conditioning. This addresses humid interior or protected exterior conditions as tested for engineered wood products. Additionally, more harsh accelerated exposure tests used a wet cycle with water immersion for 1 h after 23°C/25% RH for 23 h, followed by direct testing in wet state. Both conditions of 100% RH at 25 °C as well as full submersion in water for 1 hour were tested to cover a wide range of simulated environmental conditions.

When curing the adhesive at 60 °C already an acceptable moisture resistance is observed, with performance declining by roughly 20% for both exposure to 100% RH and full submersion (**Figure S 55**). When curing at 120 °C the moisture resistance increased, likely due to the more developed network decreasing the ability of the adhesive to swell in water. In this case, storing the adhesive joints at 100% RH for 24 h even slightly improved the lap-shear strength, which is known e.g. for polyamides, where water conditioning reduces brittleness. However, student's t-test indicate a not statistically significant difference from the reference. For full submersion of the samples only a 13% decrease in lap-shear strength is observed, showing the overall great moisture stability of the lignin-based adhesives, even under harsh conditions.

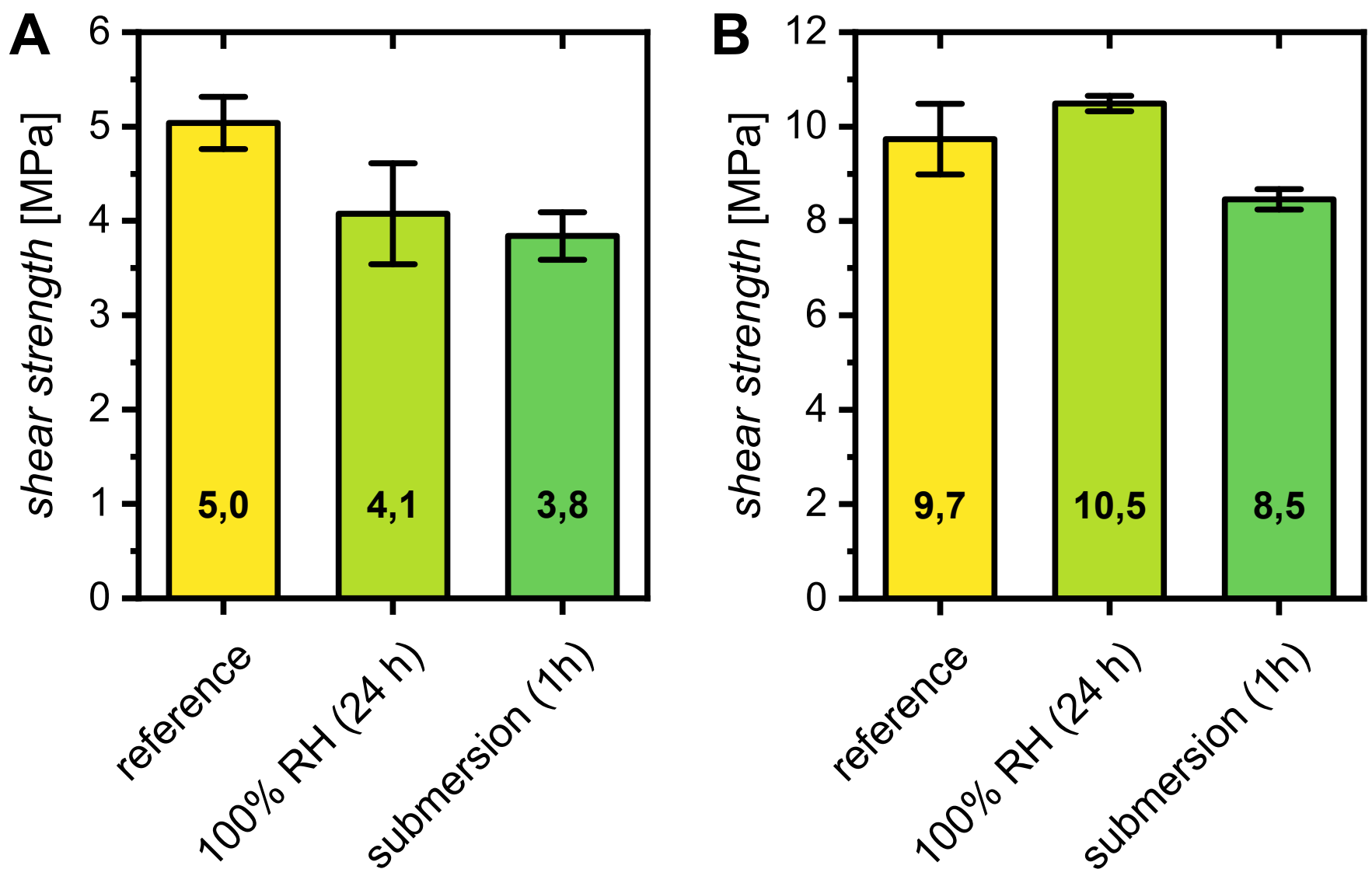

Figure S 55. Lap-shear strengths of aluminum joints bonded with lignin TGC adhesive (1 mmol/g PIDA, lignin/ETTMP1300 50/50 wt%) cured at 60 °C (A) or 120 °C (B) and subjected to different simulated environmental condition at 25 °C. Samples were stored for 24 h at 25% relative humidity (RH) (reference), 24 h at 100% RH or 23 h at 25% RH and then fully submerged for 1 h prior to testing. Error bars represent standard deviation, n=3.

To quantify the gel content of the different lignin-based adhesive resins, leaching experiments in THF were carried out (**Figure S 56**). To assess the amount of disulfide links present in the network, each resin was additionally leached under the same conditions but with tributlyphosphine added to the

leaching solution. In every case only a non-significant difference could be observed for samples leached in the presence of tributlyphosphine compared to the control without reducing agent, showing that disulfides likely are not present in the cured resins. In general, gel content increases with both curing temperature and degree of oxidation of the lignin, as both these parameters increase crosslinking density. The resin utilizing non-oxidized lignin cured at 60 °C was the only sample with a gel content of 0%, meaning it fully dissolved in THF. Even oxidizing the lignin with just 2 mmol/g PIDA results in greatly increased gel content with values of ≥90%, regardless of curing temperature.

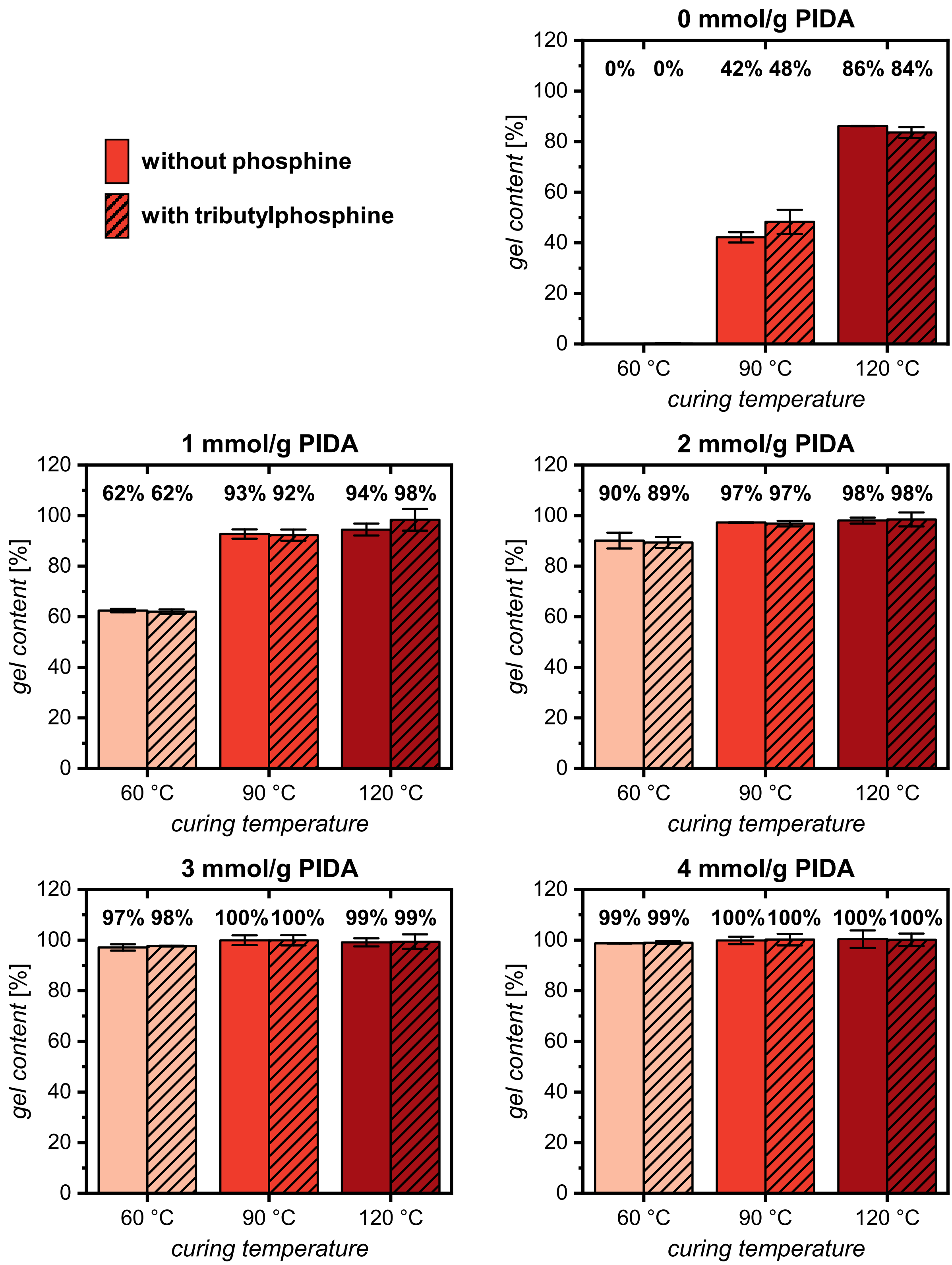

Figure S 56. Gel content of the 2K adhesives consisting of lignin (non-oxidized or oxidized with 1/2/3/4 mmol·g^{-1} PIDA) and ETTMP$_{1300}$ (50/50 wt%), cured for 16 h at 60 °C, 90 °C or 120 °C measured by leaching the cured samples in THF for 5 days and analyzing the residue gravimetrically and the solution via UV/vis spectroscopy. Results are given as the average of these methods with error bars representing standard deviation.

S6 References

[1] G. M. Sheldrick, *Acta Crystallographica Section C Structural Chemistry* **2015**, *71*, 3-8.

[2] O. V. Dolomanov, L. J. Bourhis, R. J. Gildea, J. A. K. Howard, H. Puschmann, *J. Appl. Crystallogr.* **2009**, *42*, 339-341.

[3] F. Neese, *WIREs Computational Molecular Science* **2025**, *15*.

[4] A. D. Becke, *Phys. Rev. A* **1988**, *38*, 3098-3100.

[5] E. Caldeweyher, S. Ehlert, A. Hansen, H. Neugebauer, S. Spicher, C. Bannwarth, S. Grimme, *The Journal of Chemical Physics* **2019**, *150*.

[6] F. Weigend, R. Ahlrichs, *Physical Chemistry Chemical Physics* **2005**, *7*, 3297.

[7] G. L. Stoychev, A. A. Auer, F. Neese, *J. Chem. Theory Comput.* **2017**, *13*, 554-562.

[8] F. Neese, F. Wennmohs, A. Hansen, U. Becker, *Chem. Phys.* **2009**, *356*, 98-109.

[9] V. Barone, M. Cossi, *J. Phys. Chem.* **1998**, *102*, 1995-2001.

[10] F. Weigend, *Physical Chemistry Chemical Physics* **2006**, *8*, 1057.

[11] K. Ishida, K. Morokuma, A. Komornicki, *The Journal of Chemical Physics* **1977**, *66*, 2153-2156.

[12] Y. Guo, C. Riplinger, U. Becker, D. G. Liakos, Y. Minenkov, L. Cavallo, F. Neese, *The Journal of Chemical Physics* **2018**, *148*.

[13] T. H. Dunning, Jr., *The Journal of Chemical Physics* **1989**, *90*, 1007-1023.

[14] T. Liu, C. Hao, S. Zhang, X. Yang, L. Wang, J. Han, Y. Li, J. Xin, J. Zhang, *Macromolecules* **2018**, *51*, 5577-5585.

[15] Y. Zhang, Q. Zhao, H. Shao, S. Zhang, X. Han, *Adv. Mater. Res.* **2014**, *2014*, 1-6.

[16] R. Mahou, C. Wandrey, *Polymers* **2012**, *4*, 561-589.

[17] B. Shi, M. F. Greaney, *Chem. Commun.* **2005**, 886.

[18] K. M. Herbert, P. T. Getty, N. D. Dolinski, J. E. Hertzog, D. De Jong, J. H. Lettow, J. Romulus, J. W. Onorato, E. M. Foster, S. J. Rowan, *Chemical Science* **2020**, *11*, 5028-5036.

[19] K. Walter, D. P. Hoch, E. C. Heyl, A.-C. Ranieri, J. Hansen, A. Dallmann, H. G. Börner, *ACS Macro Lett.* **2026**, *15*, 208-214.
